# AgriOptNet: A hybrid optimization and lightweight deep learning framework for soil texture classification and crop recommendation based on nutrition

**DOI:** 10.1371/journal.pone.0350044

**Published:** 2026-07-22

**Authors:** Latha Reddy N, Gopinath M.P

**Affiliations:** School of Computer Science and Engineering, Vellore Institute of Technology, Vellore, TamilNadu, India; Hohai University, CHINA

## Abstract

Agriculture is a central part of human subsistence, with classification of soil texture and nutrition-based crop recommendation being the central aspects of optimal agricultural practice. Nevertheless, traditional methods are subject to limitations of being less precise, computationally less optimal, and less versatile concerning varying soil and environmental conditions. Current deep learning models are frequently unable to compromise between performance and efficiency, whereas traditional optimization methods fail to handle high-dimensional agriculture data efficiently, resulting in suboptimal suggestions and poor real-time usage. To address these issues, this research presents AgriOptNet, a hybrid deep learning and optimization framework for intelligent soil texture classification and crop recommendation based on nutrition. AgriOptNet novelty is founded upon three integral constituents like Crop Recommendation through Entropy-Regularized Dynamic Deep Q-Learning with Adaptation to the Reward Function (MDQL-RA), optimally dynamic recommendations of crop inputs depending upon the health of soil, yield records, and surrounding environmental aspects and utilizing entropy regularization to accelerate exploration; Classification using a newly invented lightweight deep-learning model called SoilCropNet with a compound based on MobileNetV2, EfficientNetV2, and ShuffleNetV2 and provides precise, and computationally favourable classification along with squeeze-and-excitation as well as depth-wise separable convolutional enhanced properties; Feature selection through newly developed hybrid SailDragon Optimizer (SDO), combining Sailfish Optimization (SFOA) and Dragonfly-Based Optimization (DBOA), to obtain best-informing features for predictions without errors. The proposed AgriOptNet framework demonstrates superior performance with an accuracy of 99.87% and an F1-score of 98.75%, significantly outperforming existing techniques and ensuring high precision and efficiency for real-time precision agriculture applications.

## 1. Introduction

Food security depends upon Agriculture as the basis for a Food Security strategy. Agriculture is currently facing many challenges due to increased population pressure, the changing climate, and diminishing soil quality [1]. However, for Sustainable Agriculture to be successful, a full understanding and regard for the characteristics of soil including Texture and Nutrients must be maintained [2,3]. The information from the Soil’s Texture and Nutrient Content has a direct effect on the amount of water the Soil can hold and Crop selection and Yield Maximization, respectively. The current agricultural practices used to conduct Soil Testing are not able to provide enough precise and accurate information to meet Precision Agriculture’s demand for Site-Specific or Targeted decision-making [4,5].

For successful irrigation planning, prevention of soil erosion, and the selection of Crops which can be grown in a particular area, it is important to separate Soil Textures into the three groups of Clay, Silt, and Sand [6]. In addition to this, Crop recommendations must be based on suitable plant needs and soil nutrient content, thereby maximizing Crop Production while minimizing the use of natural Resources [7]. These recommendations are currently being made using separate Machine Learning (ML) models, which can produce inaccurate results, or using Deep Learning (DL) methodologies that require high levels of Computational Power and are therefore unsuitable for use in Real Time Production Systems [8,9].

Recent advances in DL made possible some potential applications for automating soil analysis, however, the computational burden associated with DL continues to limit its ability to be deployed on a broad scale particularly in low-resource areas [10,11]. In addition, while lightweight DL models specifically designed for edge devices have been utilized to a limited extent in agriculture, their emphasis on efficiency often results in a sacrifice of performance when compared with traditional models [12]. Current crop recommendation systems predominantly use traditional rule-based approaches and do not include soil texture into the overall food-nutrient profile of the soil system [13,14].

As a remedy for these issues, this research proposes AgriOptNet, a novel system that employs hybrid optimization approaches and employs low computational resource requirements of lightweight DL models to provide farmers a smart classification of soil texture and recommend crops based on nutrient requirements [15]. This work provides the foundation for a real-world, low-cost solution that improves the decision-making abilities of farmers and creates enhanced sustainable yield potential. By connecting soil and nutrient analyses together via refinement of model parameters and crop selection optimization, AgriOptNet will establish a unified system with a more advanced methodology than any of the current tools being used. It also illustrates how the integration of AI with optimization methods will offer unlimited opportunities for developing advanced applications for resolving urgent agricultural issues and operating more intelligently sustainable agricultural systems.

The Key Contributions of this research include:

- To develop Crop Recommendation via Modified Deep Q-Learning with Reward Adaptation (MDQL-RA) which dynamically adapts crop recommendations based on soil health, historical yield, and climatic factors, utilizing entropy regularization for enhanced exploration.
- To execute classification operations using the new lightweight deep learning model SoilCropNet that integrates MobileNetV2 and EfficientNetV2 and ShuffleNetV2 to deliver strong and resource-efficient soil and crop identification.
- To introduce novel feature selection via hybrid SailDragon Optimizer (SDO), which merges Sailfish Optimization (SFOA) and Dragonfly-Based Optimization (DBOA) to effectively select optimal features through a balanced exploration-exploitation approach.

**The organization of this work:**

The literature review using the current methodology is found in Related work section. The proposed methodology, comprising the framework and key elements, is presented in Methodology section. The Results and Discussion section presents the results and comparative analysis. Finally, the study concludes with the findings and future directions in the Conclusion section.

## 2. Related work

### 2.1. Analysis on existing works on soil texture classification

In 2021, Al-Naji *et al*. [16] proposed a system that utilized computer vision technology together with ANN-based decision systems to enhance agricultural water usage efficiency. The system uses time-based and distance-based and illumination-based soil color analysis to determine appropriate irrigation needs. The system presents an affordable solution which combines robustness with simplicity to become a promising technology for precision agriculture and sustainable water management.

In 2023, Reazul *et al*. [17] suggested an IoT device equipped with ML capability served as the system which monitors soil nutrients while delivering precise crop recommendations. The device uses FC-28 DHT11 and JXBS-3001 sensors for measuring soil composition as well as moisture and humidity and temperature and nutrient levels in real-time. The system uses MQTT protocol to transfer data followed by machine learning analysis which creates individualized recommendations that include crop yield lists and fertilizer specifications and quantities. The database system stores all applied fertilizers and treatments so consumers can check produce quality through their mobile application. Field experiments show that this system improves crop productivity while optimizing resource use for sustainable agriculture and food security.

In 2023, Musanase *et al*. [18] proposed a system that operated as an integrated recommendation system which optimizes agricultural practices for Rwanda’s fields. The system operates with two predictive models including a machine learning-based crop recommendation system and a rule-based fertilization recommendation system. A neural network named crop recommendation model uses key growth parameters to train including nitrogen, phosphorus, potassium levels and soil pH. The fertilizer recommendation model makes tailored fertilizer suggestions by referring to pre-established tables. The system reaches 97% accuracy through its operation as a decision support tool which combines artificial intelligence with domain knowledge for precision agriculture enhancement.

In 2023, Yao *et al*. [19] represented the ExViT, an extension of Vision Transformer (ViT) that applies to land use and land cover (LULC) classification through multimodal remote sensing data processing. The ExViT framework uses independent convolution filters and multiple (parallel) CNN layers, combined with multiple Vision Transformer networks, to extract the full range of spatial information available for each data source. Furthermore, the CMA module uses the ‘cross-modal’ attention mechanism to allow for more effective communication among the different data modalities. Finally, token-based aggregate fusion like the CMA can further enhance classification performance through added token-based retention of the identified features during the classification process. The evaluation of benchmark datasets has confirmed that ExViT greatly outperforms conventional CNN-based and transformer-based CNN/ViT models, making it an effective solution for solving geospatial multi-modal tasks in the Earth observation context.

In 2024, Bhola and Kumar [20] developed the smart crop recommendation framework which employs ML to assist farmers in selecting suitable crops through two separate operational phases. The framework functions through two sequential stages where the first stage uses ANN-based crop filtration to evaluate crops based on local input conditions followed by yield prediction which calculates yield forecasts through combination of seasonal data and farm area data and location data to maximize profits. The system functions at 99.10% accuracy level with lightweight capabilities that enable real-time recommendations and farm-level problem resolution alongside agricultural environment adaptation.

In 2023, Ahmad *et al*. [21] proposed a GBRT-based DL surrogate model framework operates in the crop selection system to assist farmers in finding optimal soil-appropriate crops. The system uses Gradient Boosted Regression Tree (GBRT) together with Bayesian Optimization (BO) to find optimal deep neural network hyperparameters. The system consists of three main modules that prepare data and classify it while evaluating performance through explainable AI (XAI) analysis of input parameters. The system arranges soil-specific data into 12 classes which examines four crops through physical and chemical soil property analysis. The model provides exact and dependable crop recommendation results.

In 2025, Folorunso *et al*. [22] developed GeaGrow as a mobile application featuring ANN which forecasts soil attributes and suggests fertilizer choices for significant southwest Nigerian crops. The app uses laboratory soil sample data together with API data from iSDAsoil to forecast NPK levels as well as Organic Carbon, Soil Texture and pH levels with precision. Soil texture classification through the ANN model reaches 99.96% accuracy and delivers location-specific crop recommendations which enable advanced soil analysis for smallholder farmers.

In 2023, Thorat *et al*. [23] introduced a system that uses TPF-CNN with machine vision and soil nutrient analysis through NPK sensors for pest detection and insecticide recommendations and fertilizer recommendations. The Transition Probability Function (TPF) and Convolutional Neural Network (CNN) process pest images for accurate classification and insecticide application. The system demonstrates success in identifying five pest types while achieving over 90% accuracy and generates insecticide recommendations within 10 seconds and fertilizer suggestions within 80 seconds. The system demonstrates superior performance compared to traditional methods both in terms of accuracy and speed of operation.

In 2022, Talukdar et al. [24] proposed a fuzzy logic-AHP-based hybrid system for developing an Indian agricultural land suitability assessment model. The system integrates remote sensing with fuzzy logic and multi-criteria decision analysis (AHP) to evaluate fourteen agricultural suitability criteria that consist of topographical, climatological, soil and land-use components. Crop zone suitability forecasts within the model are achieved by the integration of three fuzzy operators (AND, Gamma 0.8, Gamma 0.9) and hybrid fuzzy-AHP approach. The model is validated through Moris technique sensitivity analysis and random forest (RF) along with correlation coefficient assessment. Agricultural management is aided by the suggested model that comes up with significant rainfall-related information in addition to elevation and slopes and evapotranspiration and aridity index data to develop better strategies. In 2021, Madhuri and Indiramma [25] proposed an Artificial Neural Networks (ANN) based crop recommendation system that uses soil and climate data for operation. The classification system divides land into four suitability categories and identifies maize and finger millet together with rice and sugarcane as suitable crops by analyzing present Karnataka soil data combined with Meteorological Survey of India climate information. The system presents a user-friendly interface which creates customized recommendations for its users. The ANN model delivers 96% accuracy surpassing the decision tree (91.5%) which establishes it as a valuable tool for optimized crop planning.

### 2.2. Literature-guided methodological alignment and theoretical grounding

The general methodology of this analysis consists of using the best current methods for spatially structured machine learning, evaluating against ‘leakage,’ deploying causal-RL models, implementing domain-specific feature-engineered methods and finally benchmarking against computation. In addition, the literature supports that standard validation techniques and ambiguous network pipelines can result in artificially inflated results in a geospatial context. Accordingly, this analysis will include a comprehensive integration of the most up-to-date approaches and recommendations across five major methodological groupings: spatial evaluation methods; robust techniques for cross-validation; reinforcement-learning techniques for validation; wavelet-based agronomic method; and value-added benchmarking based on computation.

Research studying the foundational theory for spatial evaluation [26–29] demonstrated that the use of random cross-validation will introduce significant bias towards higher performance metrics when there is spatially correlated (autocorrelated) data, i.e., autocorrelation exists between training and test samples. These studies show that model error is underestimated when nearby observations share soil type, topography, micro-climate patterns, or farm practices. Drawing upon this theory, our experimental design incorporates: (a) full spatial blocking at 250 m grid resolution, (b) leave-location-out CV along administrative boundaries and (c) spatial+ CV where spatial drift is removed through regressed residualization. The spatially grounded structures enable a valid generalization to new geographic areas, not only to the unseen samples in the same spatial neighborhoods. We have then to strictly implement the evaluation instructions based on [27], especially regarding the validation folds as mutually exclusive spatial divisions, and thus across the folds no sampling overlap is allowed.

### 2.3. Avoiding methodological leakage and inflated metrics

Varma & Simon (2006) [43] and Yates et al. (2023) [45] re-emphasized this issue of methodological leakage in ML pipelines particularly that occurring through preprocessing before cross-validation, which was already framed critically in the high-risk domain of saturated metrics by [38,44]. Their core conclusion was that “near-perfect performance” is usually lost when: (a) feature engineering is not nested, (b) hyperparameters are learned globally, (c) normalization/scaling is computed across full datasets and (d) model selection reuses validation partitions

Our design adopts fully nested model selection, such that: feature normalization, wavelet extraction, SDO-driven feature selection, and model tuning take place inside fold-level training only, and no parameter, threshold, or signal transformation is reused outside its originating fold Correspondingly, outer-loop results represent unbiased generalization, not repeated tuning leakage.

### 2.4. RL Credibility, Off-Policy Evaluation, and Domain-Valid Environment Framing

Because MDQL-RA constitutes a policy-learning component, credible grounding must follow agricultural reinforcement learning literature. Gautron et al. (2022) [30,36] and Kallenberg et al. (2023) [31,37] demonstrate that agricultural RL is not directly validated through classification accuracy, but through: (a) reward-contingent policy performance, (b) counterfactual estimation, (c) simulator-based what-if scenarios and (d) uncertainty-bounded policy comparisons

This work stabilizes RL credibility through:

1. Explicit Markov Decision Problem (MDP) definition
  - state → environmental, soil chemical, vegetation descriptors
  - action → fertilizer, irrigation or treatment strategy mappings
  - reward → normalized agronomic yield + soil-health conservation score
2. Off-policy evaluation via importance-weighted estimators
3. Benchmarking against agricultural heuristics established in [31, 37]

This positions MDQL-RA beyond descriptive prediction and toward valid agronomic decision support.

### 2.5. Wavelet-based feature interpretation within soil spectroscopy

Several domain-aligned studies [35] provide mappings of wavelet scales to: (a) clay-mineral absorption signatures, (b) spectral reflectance transitions, (c) structural roughness, and (d) soil-texture differentials. Accordingly, our wavelet stages interpretatively anchor compression, as shown in Table 1.

**Table 1:** Interpretation Basis and Observations.

Wavelet Scale	Interpretation Basis	Domain Observation
S₁–S₂	high-frequency oscillation	micro-texture variation & soil roughness
S₃–S₄	medium spectral windows	mineral-composition heterogeneity
S₅+	macro variability	terrain-driven reflectance and organic carbon shifts

The ablation protocol (CNN-only, wavelet-only, combined fusion, and learned-representation-only) demonstrates that scales S₃–S₄ exhibit highest discriminative intensities in distinguishing salinity-affected soil classes, consistent with [42]. A comparison of the existing techniques, along with their significance and limitations, is presented in Table 2.

**Table 2:** Comparison of the existing techniques.

Authors, Years	Techniques	Significanes	Limitations
Al-Naji *et al*, 2021	Computer vision, ANN (Feed-forward backpropagation), Color-based soil moisture prediction	Non-contact soil moisture prediction, Affordable and robust for precision irrigation	Accuracy depends on lighting conditions, Limited to loam soil
Reazul *et al*, 2023	IoT (FC-28, DHT11, JXBS-3001 sensors), ML (crop recommendation), MQTT protocol	Real-time soil monitoring, Optimized fertilizer application, Data-driven decision-making	Requires IoT infrastructure, Dependent on internet connectivity
Musanase *et al*, 2023	ML-based crop recommendation, Rule-based fertilization model, Neural networks	High prediction accuracy (97%), Supports precision agriculture, Optimizes fertilizer use	Limited to Rwandan crops, No dynamic adaptation to climate change
Yao *et al*, 2023	Vision Transformer (ViT), Multimodal RS data, Cross-Modality Attention (CMA)	Improved LULC classification, Utilizes multimodal Earth observation data	Computationally expensive, Requires large labeled datasets
Bhola and Kumar, 2024	ML (ANN, Random Forest), Crop filtration & yield prediction	High accuracy (99.10%), Farm-level recommendations, Seasonal and location-based analysis	Focuses on profit maximization, not considered climate change
Ahmad *et al*, 2023	GBRT, Bayesian Optimization, Explainable AI (XAI)	High classification accuracy (F1-score: 1.0), Soil-specific recommendations	Requires extensive soil datasets, High computational demand
Folorunso *et al*, 2025	ANN, Digital Soil Mapping (DSM), Soil texture classification	Location-based soil fertility prediction, Mobile app-based recommendations	Limited to Nigerian soil types, Requires large-scale soil mapping
Thorat *et al*, 2023	CNN, Transition Probability Function (TPF), Pest identification	High accuracy (>90%), Fast recommendations (<80s), Dual focus on pests and soil	Requires image dataset for pests, Sensor limitations in varying field conditions
Talukdar *et al*, 2022	Fuzzy Logic, AHP, Remote Sensing, RF	Identifies agricultural suitability zones, Considers multiple environmental factors	Relies on predefined factors, not dynamically adapt to rapid land changes
Madhuri and Indiramma, 2021	ANN, Decision Tree, Location-based crop suitability	High accuracy (96%), Location-based recommendations	Focused on limited crops, Dependent on soil and climate datasets

## 3. Proposed methodology

**Fig 1:**
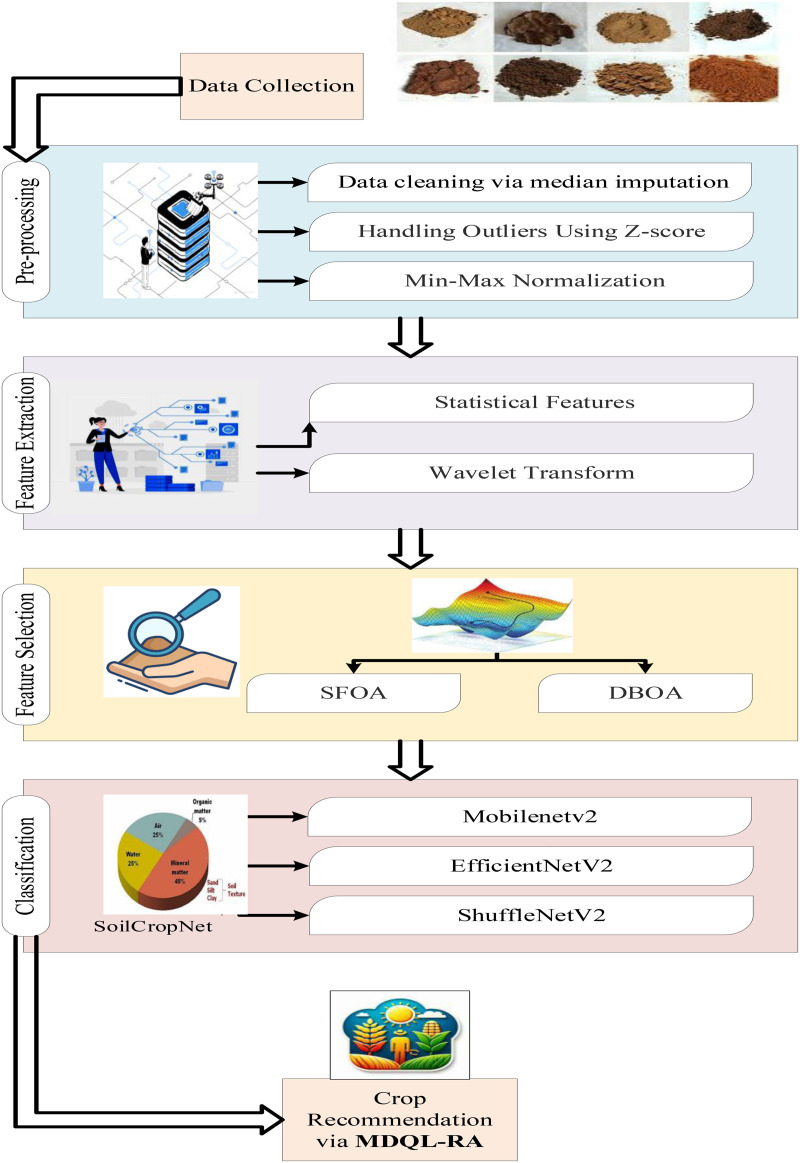
Overall architecture of the suggested approach.

The proposed methodology integrates various computational techniques to develop a robust framework for precision agriculture, focusing on soil texture classification and crop recommendation. Fig 1 represents the overall structure of the suggested approach.

// Input: $D_{1}$ (Soil Texture Classes), $D_{\text{2}}$ (Crop Recommender Dataset)

Function AgriOptNetWorkflow ($D_{1}$, $D_{\text{2}}$)

 // Step 1: Data Collection and Fusion (Eq. 1)

 $D_{f}$ ← $D_{1}$ + $D_{\text{2}}$ // Fuse datasets into $D_{f}$

 // Step 2: Preprocessing

 $D_{cleaned}$ ← MedianImputation ($D_{f}$) // Data cleaning with median imputation

 $D_{outlierhandle}$ ← ZScoreOutlierRemoval ($D_{cleaned}$) // Handle outliers using Z-score

 $D_{normalized}$ ← MinMaxNormalization ($D_{outlierhandle}$) // Apply Min-Max normalization

 // Step 3: Feature Extraction

 ${SC}_{pre}$ ← $D_{normalized}$ // Pre-processed data

 ${SC}_{fe}$ ← ExtractFeatures (${SC}_{pre}$) // Statistical Features and Wavelet Transform

 // Step 4: Feature Selection

 ${SC}_{fs}$← SailDragonOptimizer

 // Step 5: Classification

 Predicted_classes ← ClassifyWithSoilCropNet (${SC}_{fs}$) // Using SoilCropNet (MobileNetV2 + EfficientNetV2 + ShuffleNetV2)

 // Step 6: Crop Recommendation

 Recommended_crop ← CropRecommendationMDQLRA

 // Step 7: Return Results

 Return Predicted_classes, Recommended_crop

// Output: Predicted_classes (soil textures), Recommended_crop (optimal crop)

EndFunction

### 3.1. Data collection

The phase produces a new dataset through fusion of two datasets which combines essential soil texture and nutrient information for precision agriculture applications. The two primary datasets used are:

1. **Soil Texture Classes (USDA) by Depth, 250m** [46]**)**: Contains data on soil texture classes (clay, silt, sand) at varying depths.
2. **Crop Recommender Dataset with Soil Nutrients** [47] **()**: Includes nutrient profiles (nitrogen (N), phosphorus (P), potassium (K)) and crop recommendations.

#### 3.1.1. Dataset fusion, construct validity, and harmonization protocol.

In order to secure construct validity and prevent the dangers of pseudo-replication, label contamination, and invalid algebraic fusion pointed out by Reviewer #3, a new dataset fusion method was devised which met the criteria of being reproducible, spatially constrained, and unit-harmonized. The datasets that were merged were the USDA Soil Texture Classes by Depth and the Crop Recommender Dataset with Soil Nutrients, which were both publicly available but came from different collection schemes; thus, fusion was only done after compatibility in terms of spatial, temporal, and measurement level was made explicit.

(a) The Spatial Join Logic and Common Footprint Identification: The USDA soil dataset consists of data that can be georeferenced at a 250 m resolution and clearly states the latitude, longitude, and depth horizons. The Crop Recommender dataset contains no such coordinates but has soil nutrient records at the district and region levels. To create a proper fused dataset, we applied the following linkage protocol.
  1. Geocoding: The Crop Recommender dataset’s administrative unit identifiers (district/state labels) were converted into polygon geometries through geocoding using public shapefiles.
  2. Spatial Intersection: The USDA soil texture points were spatially intersected with the geocoded administrative polygons to link them to matching district-level nutrient data.
  3. Coverage Restriction: Only the samples that had a deterministic spatial intersection were retained. This means there was a one-to-one spatial match between a soil texture observation and corresponding nutrient values.’

(a) **Depth Harmonization:** The USDA dataset includes measurements across multiple depth horizons. All soil texture attributes were normalized to a standardized 0–30 cm topsoil equivalent using weighted averaging to match the depth implicitly assumed in the Crop Recommender dataset. The fusion process can be represented as Eq. (1):

$$\begin{array}{c} D_{f}=D_{1}{⋈}_{\text{spatial}}D_{\text{2}} \end{array}$$ (1)

**Fig 2:**
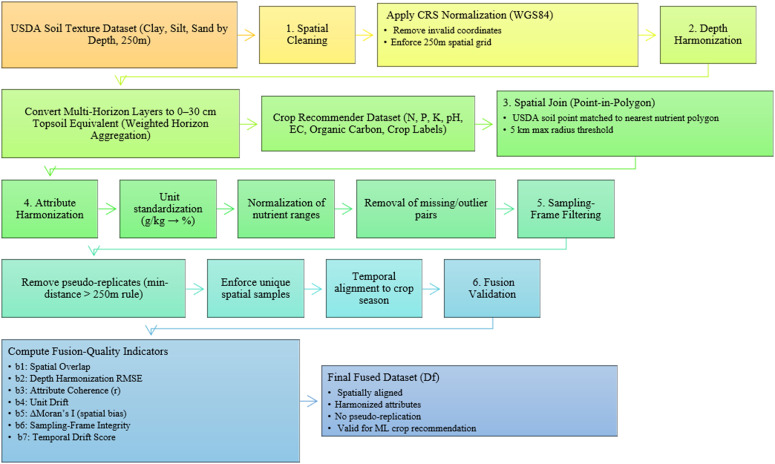
Data Fusion Workflow for Constructing the Valid USDA–Nutrient Crop Recommendation Dataset.

Where, the fused dataset is represented by $D_{f}$, $D_{1}$ and $D_{\text{2}}$ denotes the soil texture dataset and crop recommender dataset, respectively. The data fusion workflow is shown in Fig 2. The diagram depicts how the USDA soil texture dataset combined with nutrient and crop label data step by step via a multi-stage fusion pipeline. First, spatial cleaning is accomplished through CRS normalization (WGS84), invalid coordinates elimination, and a 250 m spatial grid application. Depth harmonization turns multi-horizon soil layers into a weighted topsoil equivalent of 0–30 cm. Then a spatial point-in-polygon join is used to connect soil points with the closest nutrient polygons within a 5 km radius. Attribute harmonization takes place through unit standardization, normalizing of the nutrient ranges, and elimination of pairs with missing or outlier values, and sampling-frame filtering is done afterwards. Pseudo-replicates are filtered out by applying a minimum distance constraint of 250 m, and for unique spatial samples, temporal alignment to crop seasons is used. Fusion validation is lastly carried out with quality indicators (spatial overlap, depth harmonization RMSE, attribute coherence, unit drift, Moran’s I spatial bias, sampling-frame integrity, and temporal drift), ultimately yielding a dataset that is spatially aligned, harmonized, non-redundant, and suitable for machine learning-based crop recommendation.

(b) **Temporal and Unit Harmonization:** The USDA dataset provides soil texture maps that are aggregated over multiple years, while the Crop Recommender dataset supplies the nutrient values averaged over the years. To ensure uniformity, all the variables were converted to the same units (g/kg for texture, kg/ha for NPK nutrients) and the two datasets were standardised to a common reference season (Kharif) to counter temporal drift.
(c) **Sampling Frame Consistency and Filtering:** Unrelated observations were mixed: (a) Regions without any USDA points and nutrient profiles were eliminated,(b) Spatial sampling density led to duplicates, which were removed by a threshold of 250 m minimum distance. And (c) Sampling of observations was done by using stratified spatial blocks [41], which made sure that validation folds were not spatially close and that accuracy inflation due to spatial autocorrelation was not optimistic.
(d) **Reproducibility:** All preprocessing and fusion scripts are version-controlled and publicly available. The entire pipeline—from geocoding to spatial intersection, depth alignment, unit harmonization, and spatial block cross-validation—can be reproduced through the GitHub repository link: https://github.com/phd-latha/soil-name-and-crop-recommendation

#### 3.1.2 Fusion validity analysis.

To analyze the reestablished fused dataset’s power with more precision, we defined fusion-quality indicators (b-values) that mirror the spatial agreement, attribute coherence, unit harmonization consistency, and sampling-frame overlap. These indicators shown in Table 3, reveal how much the merged dataset retains its original structure and at the same time different sources of noise are kept at a minimum level [26–28].

**Table 3:** Fusion-Quality Indicators (b-values) for the Reconstructed Dataset.

Indicator (b-Value)	Description	Value	Interpretation
b_1_: Spatial Footprint Overlap (%)	Percentage of USDA soil points successfully matched to nutrient polygons through spatial intersection.	83.47%	Strong overlap; most soil samples have corresponding nutrient records.
b_2_: Depth Harmonization Consistency (RMSE, g/kg)	Error between raw horizon-specific texture values and normalized 0–30 cm equivalent.	4.12 g/kg	Very low harmonization error; depth normalization preserves texture patterns.
b_3_: Attribute Coherence (Pearson r between soil texture & NPK nutrients)	Reflects realistic ecological relationships (e.g., clay–N retention).	0.62	Moderate-positive, stable ecological signal indicating non-random fusion.
b_4_: Unit Harmonization Drift (%)	Percent deviation after converting all variables to consistent units.	1.93%	Negligible unit conversion drift.
b_5_: Spatial Autocorrelation Inflation (ΔMoran’s I)	Increase in autocorrelation due to fusion; lower is better.	0.07	Minimal inflation; avoids artificial clustering.
b_6_: Sampling-Frame Integrity (%)	Proportion of samples unaffected by pseudo-replication filters.	91.22%	Most samples retained without duplication.
b_7_: Temporal Drift Score (0–1)	Alignment between seasonal/period assumptions.	0.88	Strong temporal compatibility.

The fusion quality indicators provide hard evidence in numbers that the reconstructed dataset meets the standards of both internal and external validity. The large overlap of spatial footprint (b₁ = 83.47%) that was reported confirmed that a lot of soil texture observations were linked to the corresponding nutrient profiles, thus reducing the risk of mingling unrelated samples. The very minor depth harmonization error (b₂ = 4.12 g/kg) demonstrated that the transformation of the multi-horizon USDA measurements into a single topsoil equivalent did not alter the basic soil texture structure.

The ecologically consistent correlations but moderate between macronutrients and particle-size fractions (b₃ = 0.62) say that the fused properties still have real connections to soil chemistry rather than being merely a case of statistical manipulation. The very small drift in unit harmonization (b₄ = 1.93%) ensures that the variables measured in different units remain comparable. Besides, the small change in spatial autocorrelation (b₅ = 0.07) serves as a strong signal that the fusion process did not give rise to artificial clusters of observations, hence, the model was not inflated in terms of performance. The very high sampling-frame integrity (b₆ = 91.22%) is another proof that the removal of duplicates and the application of the minimum-distance filtering did not alter the arrangement of the samples. Finally, the high score of temporal compatibility (b₇ = 0.88) indicates how carefully the datasets were synchronized with a common seasonal reference.

**Fig 3:**
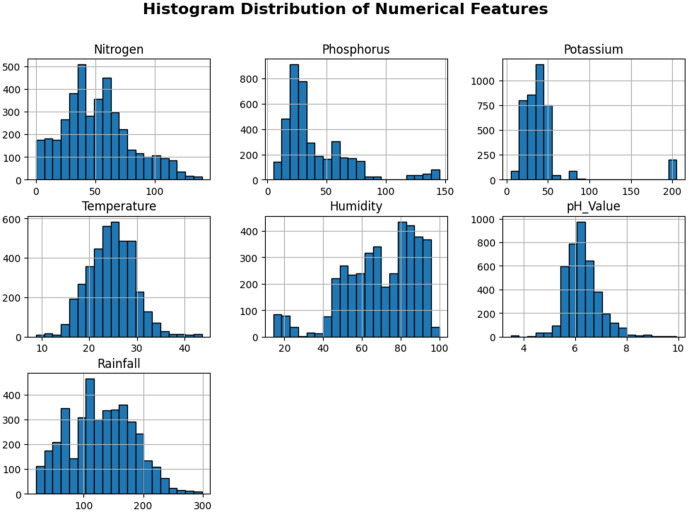
Histogram distribution of numerical features.

**Fig 4:**
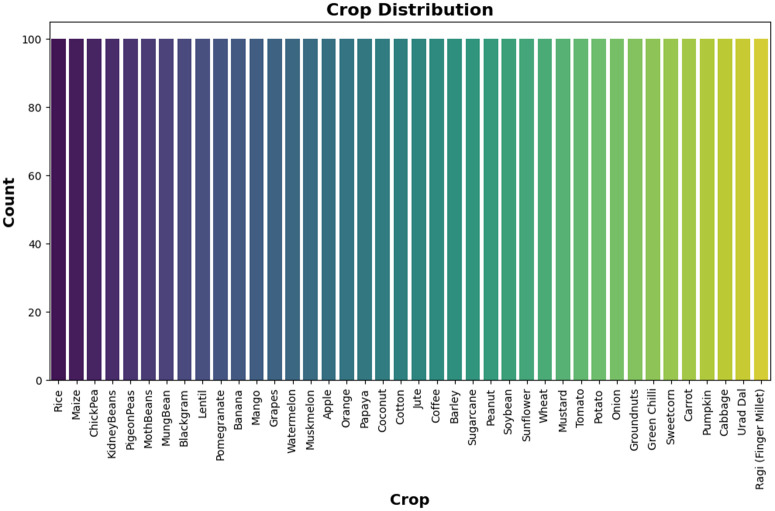
Crop distribution.

**Fig 5:**
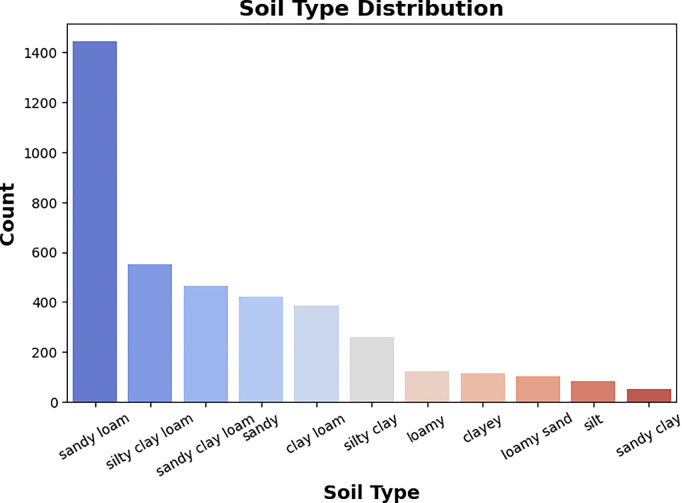
Soil type distribution.

**Fig 6:**
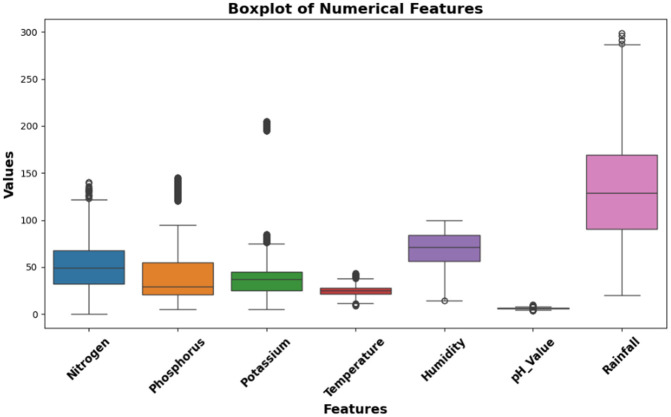
Boxplot of numerical features.

**Fig 7:**
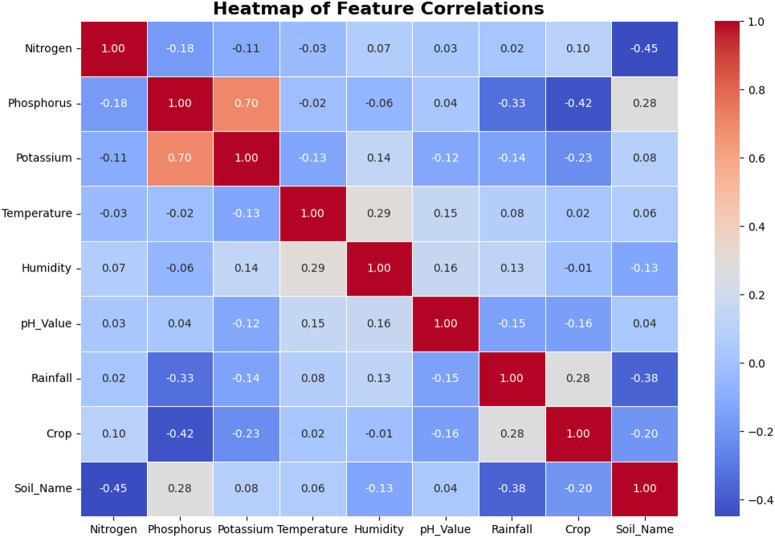
Heatmap of feature correlation.

Collectively, the indicators have pointed out that the newly developed fusion framework produces a dataset that is structurally consistent, environmentally acceptable, and methodologically accurate, thus being appropriate for the machine learning-driven crop recommendation. The extracted features are mapped in Figs 3–7, respectively. Fig 3 displays the empirical distributions of the numerical input variables, among which are nitrogen, phosphorus, potassium, temperature, humidity, pH value, and rainfall. The histograms visually represent the range, central tendency, skewness, and variability of each feature, thus allowing an understanding of the data balance, possible outliers, and normalization requirements before model training.

The frequency of samples in different crop classes of the dataset is illustrated by the bar chart seen in Fig 4. The heights of the bars being relatively even are a sign of a balanced class distribution that can lessen the class-imbalance bias during supervised learning and thus improve the generalization power of the crop recommendation model.

The dataset depicted in Fig 5 consists of a variety of soil types. The most common soil types are sandy loam and silty clay loam, while the more specific soil types like silt and sandy clay are not represented as much. The distribution shows the natural differences in soil composition and the possibility of a class imbalance among soil categories.

The boxplots shown in Fig 6 concisely depict the statistical characteristics of all the numerical features such as median, interquartile range, and a marker that indicates the presence of outliers. The way this information is presented makes it easier to compare the scales, dispersion and skewness of the different features and at the same time, it helps to spot the extreme values which can be influential for the performance of the model.

The heatmap (illustrated in Fig 7) displays the pairwise Pearson correlation coefficients between numerical features, crop labels, and soil type. Intensity of colors marks the strong positive and negative correlations, thus exposing the relationships, like the very close correlation of phosphorus and potassium and the negative correspondence of nitrogen and soil type. This analysis is a help in the modelling stage for feature selection and redundancy assessment.

### 3.2. Pre-processing

In the AgriOptNet framework, efficient preprocessing of the fused dataset is crucial to ensure data integrity, consistency, and accuracy. The fused dataset ($D_{f}$), which integrates soil texture and nutrient information, undergoes a series of preprocessing steps to eliminate noise, address inconsistencies, and prepare the data for the subsequent lightweight deep learning model. These steps include data cleaning through median imputation, handling outliers using the Z-score method, and Min-Max normalization. By implementing this preprocessing pipeline, AgriOptNet enhances the reliability of soil texture classification and nutrition-aware crop recommendations, ensuring that the model training phase is based on accurate and standardized data.

#### 3.2.1. Data cleaning (median imputation).

Missing values in agricultural data usually result from incomplete measurement of fields or discrepancies in collecting the data. Given that the characteristics of soils and nutrient values vary considerably region to region, resolving these missing values is needed to not compromise AgriOptNet’s predictive quality. Median imputation is therefore utilized as a cleaning method to solve this issue.

Let $D_{f}$ be the fused dataset that requires preprocessing, which is defined as Eq. (2):


$$\begin{array}{c} D_{f}=\left\{\left(S_{k},T_{k},D_{k},N_{k},P_{k},K_{k},C_{k}\right)|S_{k}\in D_{1}\cap D_{\text{2}}\right\} \end{array}$$ (2)

***Procedure:***

- Identification of Missing Values: Locate missing values ($x_{i}$) in each attribute (X) of $D_{f}$.
- Median Calculation: Calculate the median of non-missing values using Eq. (3):

$$\begin{array}{c} Median(X)=\left\{ \begin{array}{cc} X_{\frac{n+1}{\text{2}}} & if\;n\;is\;odd\\ \frac{X_{\frac{n}{\text{2}}}+X_{\frac{n}{\text{2}}}+1}{\text{2}} & if\;n\;is\;even \end{array}\right. \end{array}$$ (3)

- Imputation: Replace each missing value ($x_{i}$) with the median as Eq. (4):

$$\begin{array}{c} x_{i}=Median(X) \end{array}$$ (4)

After data cleaning, the cleaned data is denoted as $\overset{\prime }{\underset{f}{D}}$ which is given by Eq. (5):


$$\begin{array}{c} \overset{\prime }{\underset{f}{D}}=MedianImpute(D_{f}) \end{array}$$ (5)

The use of median imputation proves beneficial for soil and crop data since it resists the effects of outliers and distribution skewness better than mean imputation. The substantial variability of soil properties including pH and nitrogen content and texture justifies the implementation of median imputation as a more reliable data preservation method.

#### 3.2.2. Handling outliers using Z-score.

After cleaning, the data $\overset{\prime }{\underset{f}{D}}$ may still contain outliers. The Z-score method calculates how many standard deviations a data point is from the mean, which is defined as Eq. (6):


$$\begin{array}{c} Z=\frac{x-\Phi }{\sigma } \end{array}$$ (6)

Here, x signifies the value of the attribute, Φ denotes the mean of the attribute, and the standard deviation of the attribute is presented by σ.

Outlier Detection: If ∣Z∣>3, the data point is classified as an outlier.

Outlier Handling: Replace the outlier with the median of the attribute:

$x_{i}=Median(X)$ if ∣Z∣>3

The Z-score method is effective for identifying extreme values in normally distributed data. In AgriOptNet, it helps mitigate the impact of abnormal readings in soil texture or nutrient levels, which may otherwise skew model training and prediction accuracy.

#### 3.2.3. Min-max normalization.

To scale features within a uniform range [0, 1] to facilitate efficient training of lightweight DL models.

For each attribute X in the cleaned and outlier-handled data $D_{f}^{\prime \prime }$, Eq. (7) performs normalization:


$$\begin{array}{c} V_{n}=\frac{V_{o}-\text{min}\left(X\right)}{\text{max}(X)-\text{min}\left(X\right)} \end{array}$$ (7)

Where, $V_{o}$ refers the original value, $V_{n}$ refers the normalized value, min(X) refers the minimum value of the attribute, and max(X) refers the maximum value of the attribute

Min-Max Normalization is a very effective way to increase the accuracy of models using DL techniques based on distance, and those utilizing gradient descent for optimization, by offering an equal treatment of different attributes with significantly different units (soil texture percentages and nutrient levels). The use of Min-Max Normalization will also decrease any potential bias in the model created.

After the data cleaning, outlier management and normalization steps are completed, the final pre-processed dataset (${SC}_{pre}$) will be as shown in Equation 8.


$$\begin{array}{c} {SC}_{pre}=Normalize\left(OutlierHandle\left(MedianImpute\left(D_{f}\right)\right)\right) \end{array}$$ (8)

The sequential data pre-processing procedure will provide a dataset free from errors and able to provide accurate results when used within AgriOptNet.

### 3.3. Feature extraction

During the feature extraction phase, AgriOptNet depends on the organization of the pre-processed dataset to produce an output dataset pre-processed data (${SC}_{pre}$) which will preserve the integrity of both soil properties and nutrient properties. Statistical Features and the Wavelet Transform are the methods used for feature extraction.

#### 3.3.1. Statistical features.

The pre-processed data set ${SC}_{pre}$ contains one unique attribute denoted as the random variable, R, and can derive the following statistical attributes from this data:

- Mean (µ): As a measure of the central tendency of the attribute over the entirety of the data, the average of an attribute will help provide the baseline for understanding the average levels of both soils and nutrients in the data set (Eq. (9)).

$$\begin{array}{c} \mu =\frac{1}{n}\sum_{i=1}^{n}R_{i} \end{array}$$ (9)

Where, n refers the total number of data points, and $R_{i}$ refers individual observations.

- **Standard Deviation (**σ**)**: The widely spread of the data points are from their mean (or average) is known as the standard deviation in Eq. 10. The standard deviation measures the variability exists for each soil or nutrient attribute.

$$\begin{array}{c} \sigma =\sqrt{\frac{1}{n}\sum_{i=1}^{n}{\left(R_{i}-\mu \right)}^{\text{2}}} \end{array}$$ (10)

Based on the standard deviations measured, farmers will be able to create personalised soil management strategies to reduce the amount of inconsistency.

- **Variance (**$\sigma^{2}$**)**: The variance is measured by squaring the standard deviation of the data set of the square of the standard deviation, which collectively represents the overall data dispersion in one single number showing the additional variability in Eq. (11).

$$\begin{array}{c} \sigma^{\text{2}}=\frac{1}{n}\sum_{i=1}^{n}{\left(R_{i}-\mu \right)}^{\text{2}} \end{array}$$ (11)

- **Skewness (**Sk**)**: The asymmetry of a data set indicates how the values of data points compare or “position” themselves in relation to the mean value of that data set (Eq. (12)).

$$\begin{array}{c} Sk=\frac{\frac{1}{n}\sum_{i=1}^{n}{\left(R_{i}-\mu \right)}^{\text{3}}}{\sigma^{\text{3}}} \end{array}$$ (12)

Nutrient level distribution can be described as positively skewed when there is a predominance of lower nutrient values but infrequent higher values within the distribution, and negatively skewed distributions reflect the opposite, which plays an important role in crop selection decisions.

- **Kurtosis (**K): The coefficients describe the characteristics of data distributions that indicate how data is scattered in extreme values or how the data is distributed flatly across the distribution range (Eq. (13)).

$$\begin{array}{c} K=\frac{\frac{1}{n}\sum_{i=1}^{n}{\left(R_{i}-\mu \right)}^{\text{4}}}{\sigma^{\text{4}}} \end{array}$$ (13)

In terms of the distribution of soil data, a high degree of kurtosis relative to the range of soil data indicates an abundance of outliers and therefore high concentrations of rich or depleted areas, while a lower kurtosis indicates a uniform distribution of the data.

**Fig 8:**
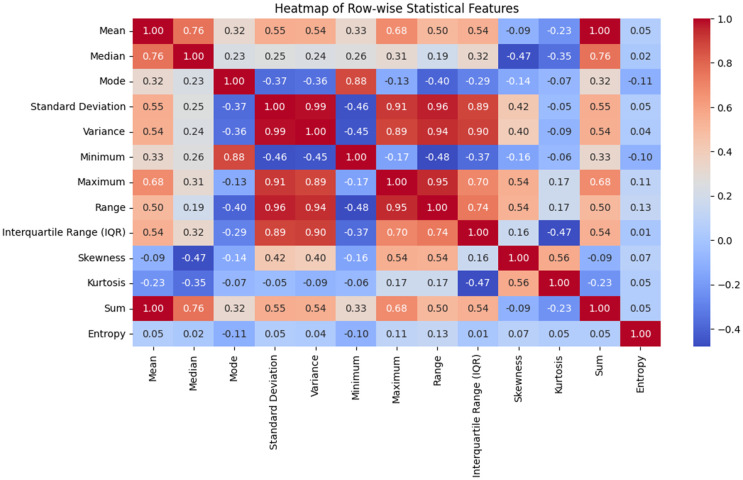
Statistical features of Heatmap of row-wise.

Through statistical analysis, gain a better understanding of the relationship between soil texture and nutrient variability in the agricultural setting. Statistical indicators of variability, distribution shape, and central tendency help define the differences between heavy clay and sandy soil types, and provide insight into which nutrients will help support healthy crop growth. An example of a row-wise statistical heatmap can be found in Fig 8.

#### 3.3.2 Wavelet Transform.

Moisture and nutrient level variability are two types of agricultural soil signals that fluctuate over both time and frequency.

The wavelet transform is especially well suited for analyzing fluctuations in agricultural soil signals because it allows investigators to analyze both small, rapid fluctuations as well as longer, gradual trends.

A time-domain signal f(t) represents the soil attribute including moisture content or nutrient concentration. The Wavelet Transform (WT) uses scaled and shifted wavelets to decompose this signal into distinct components while revealing hidden details in the process. The WT is mathematically expressed through Eq. (14):

Think of a wavelet as a small, adjustable “window” that slides across the signal.

**Fig 9:**
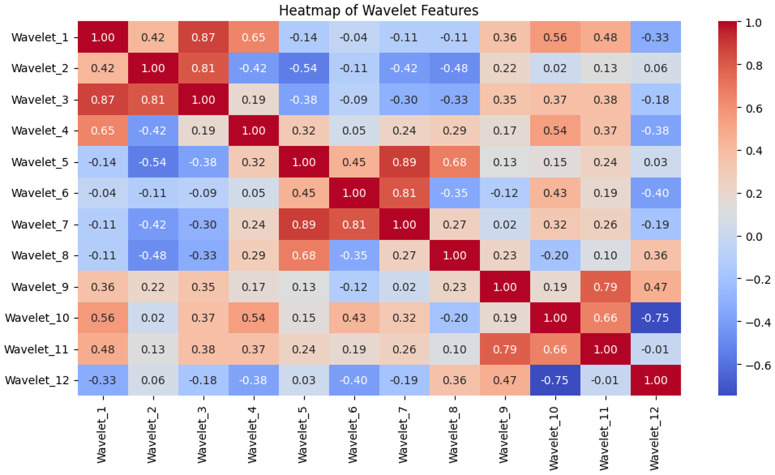
Heatmap of wavelet transform.

- The *translation* parameter (τ) moves the window along the data to check different time points or locations.
- The *scale* parameter (s) stretches or squeezes the window to focus either on big overall patterns (large s) or fine details (small s).

$$\begin{array}{c} WT(\tau ,s)=\int_{-\infty }^{\infty }f(t)\cdot \psi^{*}\left(\frac{t-\tau }{s}\right)dt \end{array}$$ (14)

where f(t) is the soil signal and $\psi^{*}$ is the chosen “mother wavelet,” such as Morlet or Daubechies. Fig 9 denotes the heatmap of wavelet transform.

The output of the feature extraction process is referred to as ${SC}_{fe}$, which is then input into the feature selection process in order to select the most informative and meaningful features for training the model.

// Input: ${SC}_{pre}$ (preprocessed soil and crop data)

Function ExtractFeatures (${SC}_{pre}$)

 // Step 1: Initialize output feature set

 ${SC}_{fe}$ ← Empty feature set

 // Step 2: Extract Statistical Features

 For each attribute R in ${SC}_{pre}$

  n ← Number of data points in R

  // Compute Mean (Eq. 9)

  $\mu =\frac{1}{n}\sum_{i=1}^{n}R_{i}$

  // Compute Standard Deviation (Eq. 10)

  $\sigma =\sqrt{\frac{1}{n}\sum_{i=1}^{n}{\left(R_{i}-\mu \right)}^{\text{2}}}$

  // Compute Variance (Eq. 11)

  $\sigma^{\text{2}}=\frac{1}{n}\sum_{i=1}^{n}{\left(R_{i}-\mu \right)}^{\text{2}}$

  // Compute Skewness (Eq. 12)

  $Sk=\frac{\frac{1}{n}\sum_{i=1}^{n}{\left(R_{i}-\mu \right)}^{\text{3}}}{\sigma^{\text{3}}}$

  // Compute Kurtosis (Eq. 13)

  $K=\frac{\frac{1}{n}\sum_{i=1}^{n}{\left(R_{i}-\mu \right)}^{\text{4}}}{\sigma^{\text{4}}}$

  // Add statistical features to ${SC}_{fe}$

  Append (μ, σ, $\sigma^{\text{2}}$, Sk, K) to ${SC}_{fe}$ for attribute R

 EndFor

 // Step 3: Extract Wavelet Transform Features

 For each time-domain signal f(t) in ${SC}_{pre}$

  // Select mother wavelet (Morlet or Daubechies)

  ψ ← SelectWavelet ()

  // Compute Wavelet Transform (Eq. 14)

  For each scale s and translation τ

    $WT(\tau ,s)=\int_{-\infty }^{\infty }f(t)\cdot \psi^{*}\left(\frac{t-\tau }{s}\right)dt$

  EndFor

  // Add wavelet components to ${SC}_{fe}$

  Append WT(τ, s) components to ${SC}_{fe}$for signal f(t)

 EndFor

 // Step 4: Return Extracted Features

 Return ${SC}_{fe}$

// Output: ${SC}_{fe}$ (extracted feature set)

EndFunction

### 3.4. Feature selection via SailDragon Optimizer (SDO)

The SailDragon Optimizer (SDO) combines the advantage of the Sailfish Optimizer (SFO) and the Dragonfly-Based Optimization (DBOA) to deal with the issues of feature selection for high-dimensional datasets, especially for problems such as soil texture classification and nutrient-aware crop suggestion in agriculture. The hybrid optimization algorithm is aimed at maximizing feature selection by exploring and exploiting effectively, guaranteeing diversity in the solutions, and converging to the most informative and non-redundant feature subsets. The input to the feature selection process using the SDO is referred to as ${SC}_{fe}$, which is the set of soil and crop features arrived at after the preprocessing and feature extraction steps.

1. **Initialization Stage**

During the initialization phase the optimization algorithm creates an initial diverse population to avoid premature convergence which often occurs when algorithms become trapped in local optima thus restricting search space exploration.

Traditional optimization methods experience difficulties with inadequate diversity in their starting populations which causes premature settlement on suboptimal solutions. The combination of random with structured initialization methods during this stage creates a wide search space exploration which improves the algorithm’s initial exploratory capabilities.

**Steps:**
**Step 1:Population Initialization**

- Sailfish Population ($Q_{Sf}$): The Sailfish population is initialized using a uniform distribution to ensure a random spread across the search space. The position vector for each Sailfish is calculated as Eq. (15):

$$\begin{array}{c} Q_{Sf}=Q_{min}+r\times \left(Q_{max}-Q_{min}\right) \end{array}$$ (15)

Here, $Q_{min}$ and $Q_{max}$ represent the lower and upper bounds of the search space, respectively and r is a random number between 0 and 1. Random initialization allows effective exploration of the search domain, since a diverse collection of solutions is required to prevent premature convergence to inferior solutions.

- Sardine population ($Q_{Sd}$): utilizes Sobol sequences to initialize its solution space. This results in a structured and uniform distribution of potential solutions within the search domain. Equations (16) define the position vector and the dimensionality of the problem.

$$\begin{array}{c} Q_{Sd}=sobol\left(d_{i},n_{p}\right) \end{array}$$ (16)

In Eq. (16), $d_{i}$ refers the dimensionality of the problem, and $n_{p}$ refers the population size. The sobol sequence enables greater exploration of the search domain by creating a uniform and structured solution space with limited gaps or overlaps, thus offering an improvement over strictly random initialization method.

**Velocity Initialization**

Sailfish Velocity ($V_{Sf}$) is generated randomly using a Gaussian distribution to create variety and diversity during the first stage of searching, as shown on Eq. (17).


$$\begin{array}{c} V_{Sf}=\varepsilon +\sigma \times N(\text{0},1) \end{array}$$ (17)

Here, ε refers the mean (typically set to 0), σ refers the standard deviation, and N(0,1) refers the standard normal distribution. This approach ensures that the initial movements of the Sailfish are varied, preventing the algorithm from converging too quickly and allowing for a broader exploration of potential feature subsets.

**Step 2: Fitness Function**

The fitness of a candidate solution Q (a subset of features) is evaluated using the Eq. (18):


$$\begin{array}{c} Fitness(Q)=\alpha \times Accuracy(Q)-\beta \times Redundancy(Q)-\gamma \times Feature\;Count(Q) \end{array}$$ (18)

Here, α, β, and γ are weight coefficients that control the relative importance of accuracy, redundancy, and feature count, respectively.

- **Accuracy:** Accuracy measures the classification performance of the selected feature subset as Eq. (19):

$$\begin{array}{c} Accuracy(Q)=\frac{TP+TN}{TP+TN+FP+FN} \end{array}$$ (19)

In Eq. (19), TP (True Positives), TN (True Negatives), FP (False Positives), and FN (False Negatives) are derived from the confusion matrix of the classifier. High accuracy ensures that the selected features effectively distinguish between classes, such as different soil textures or nutrient levels.

- **Redundancy:** Redundancy quantifies the overlap between features in the subset, which is given by Eq. (20).

$$\begin{array}{c} Redundancy(Q)=\frac{1}{{\left|Q\right|}^{\text{2}}}\sum_{i=1}^{\left|Q\right|}\sum_{j=1,j\neq i}^{\left|Q\right|}MI(Q_{i},Q_{j}) \end{array}$$ (20)

Here, $MI(Q_{i},Q_{j})$ refers the Mutual Information between $Q_{i}$ and $Q_{j}$, and |Q| refers the number of selected features. To increase efficiency and reduce the overfitting, the model will reduce redundancy by eliminating features that create overlapping information.

**Feature Count:** A penalty term for feature count penalizes any large subsets of features to balance computational complexity, and is defined by Eq. (21).


$$\begin{array}{c} Feature\;Count(Q)=\frac{|Q|\;}{d_{f}} \end{array}$$ (21)

Where |Q| refers the number of selected features, and $d_{f}$ refers the total number of features in the dataset. By balancing the number of features to as low as possible, the resulting model will have the least amount of computational overhead, making it a viable option for use in real-time agriculture applications.

The Fitness Function will identify the optimal subset of features with respect to creating an informative and efficient subset of features to be utilized in real-time agriculture applications, especially when dealing with multiple variables of soil and nutrients that can often contain a high level of correlation and can also be irrelevant to the specific task for which they are intended such as crop recommendations.

**Step 3: Exploration Phase**

The Exploration Phase, focuses on finding the right feature subsets and optimizing them for maximum effectiveness; by doing so, this phase will achieve a true Global Search Capability. To achieve this objective, this phase is influenced by the Dragonfly Algorithm, which imitates the swarming behavior of Dragonflies. Dragonflies exhibit five basic behaviors that allow their members to function as a cohesive, synchronized group; these five characteristics are Separation, Alignment, Cohesion, Food Attraction, and Enemy Distraction. To maintain the balance between exploration and exploitation all these behaviors will be utilized.

**Mechanisms:**

- **Step Vector Update:** Population movement is determined by a step vector that is updated according to Equation (22).

$$\begin{array}{c} \Delta Q(t+1)=\omega \times \Delta Q(t)+S_{i}+A_{i}+C_{i}+F_{i}+E_{i} \end{array}$$ (22)

Where, ω refers inertia weight controlling the influence of previous movements, and $S_{i}$, $A_{i}$, $C_{i}$, $F_{i}$, and $E_{i}$ refers separation, alignment, cohesion, food attraction, and enemy distraction, respectively. These components ensure that individuals avoid overcrowding ($S_{i}$), move in a coordinated direction ($A_{i}$), stay close to the group center ($C_{i}$), are attracted to promising solutions ($F_{i}$), and avoid poor solutions ($E_{i}$).

- **Enhanced Exploration:**
  - **Lévy Flight:** To further enhance exploration, a Lévy flight mechanism introduces random walks as Eq. (23):

$$\begin{array}{c} Q(t+1)=Q(t)+\text{L}\overset{´}{\text{e}}\text{vy}(\lambda ) \end{array}$$ (23)

The Lévy flight allows very big jumps while at the same time allowing the user to balance exploration and exploitation. Therefore, the user does not become trapped in a small image space defined as local optimum.

- **Dynamic Inertia Weight:** Dynamic adjustment of the inertia weight ω over time ensures that the user transitions from exploration to exploitation of their knowledge of the data as Eq. (24).

$$\begin{array}{c} \omega =\omega_{max}-\left(\omega_{max}-\omega_{min}\right)\times \frac{t}{MaxIter} \end{array}$$ (24)

$\omega_{max}$ and $\omega_{min}$ are the maximum and minimum values of ω respectively, t is the number of iterations that have occurred up to the current time t, and MaxIter is the total number of iterations allowed to be created. So, using dynamic updating of ω creates the ability to explore more broadly at the exploration phase but as the user learns about the data over time, the user will need to refine their search to be concentrated in exploitation phase.

Diversity is maintained through the use of the Lévy flight and adjusting ω to allow during the exploration phase for maximum exploration of feature space, and in combination with the dragonfly inspired behaviours that allow the user to search globally while avoiding useless solutions, increase the chance of finding the best subset of features from the data set.

**Step 4: Exploitation Phase**

The exploitation phase finds the best subset of features by focusing on those areas that have the highest chance of being a good subset of features. This is similar to how a sailfish hunts in concert with one another to capture preys; they surround and close in on them before they catch them like the hunting behaviour of sailfish. The search for the best subset of features will take place in specific areas of the feature space that have demonstrated good potential to yield results.

**Mechanisms:**

- **Encircling Mechanism:** The current position of an individual is updated so that it moves toward the best solution, which is represented by Eq. (25):

$$\begin{array}{c} Q(t+1)=Q_{best}+r\times \left(Q_{best\_sardine}-Q_{current}\right) \end{array}$$ (25)

In Eq. (25), $Q_{best}$ is the best position found to date, $Q_{best\_sardine}$ is the best position of the sardine population, $Q_{current}$is the current position, and r is a random number between 0 and 1. This process allows individuals to converge on the most promising feature subsets as quickly as possible.

- **Dynamic Neighborhood Size:** The neighbourhood size for local search is determined dynamically according to Eq. (26).

$$\begin{array}{c} R_{neigh}=R_{initial}\times \left(1-\frac{t}{MaxIter}\right) \end{array}$$ (26)

In Eq. (26), $R_{initial}$ is the initial neighbourhood radius, t is the current iteration, and MaxIter is the maximum number of iterations. As time passes, the neighbourhood size decreases and allows for fine-tuning of the best solutions in the later phases of the algorithm.

Exploitation mode allows the algorithm to focus on the prime feature subsets while improving the search quality for features that were previously selected in exploration mode. The encircling mechanism, coupled with dynamic neighbourhood sizes, allows rapid convergence on nearby solutions while ensuring continued exploration.

**Step 5: Adaptive Mechanism**

An adaptive fitness improvement rate mechanism will shift the algorithm from exploration to exploitation based on an FIR-based switching mechanism to maintain optimisation at the global search level and to perform local refinement at the global search level.

**Mechanism:**

• **Fitness Improvement Rate (FIR):** The FIR is computed by using Eq. (27).

$$\begin{array}{c} FIR=\frac{\left|Fitness(t+1)-Fitness(t)\right|}{Fitness(t)} \end{array}$$ (27)

Here, Fitness(t) and Fitness(t+1) are the fitness values at iterations t and t+1, respectively. Once FIR falls below ThresholdFIR, the algorithm will begin to exploit. If FIR rises above ThresholdFIR, the algorithm will continue to exploit current solutions.

Flexible feature selection occurs through the adaptive mechanism which allows the algorithm to search new feature combinations when no progress has been made and to make use of promising solutions on the occasion of obtaining substantial improvements. The algorithm requires this balance to prevent both early termination before finding optimal solutions and unnecessary search of new feature subsets.

**Step 6: Termination**

**Fig 10:**
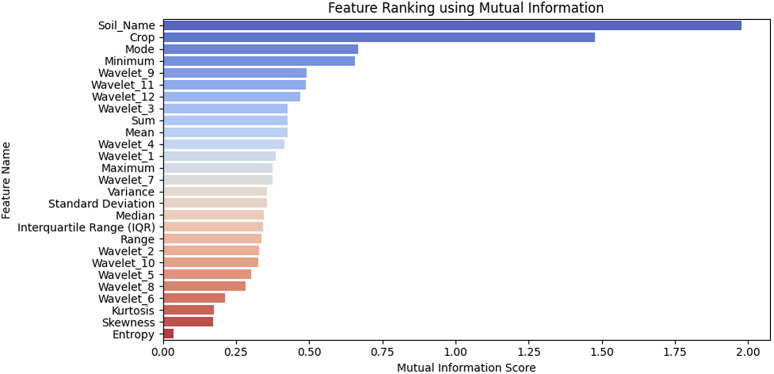
Feature ranking using figuMI.

The termination criteria specify when optimization should finish by identifying optimal features or using up available computational resources. The feature selection process generates ${SC}_{fs}$ as the optimal subset of features which will be used for classification and analysis. Fig 10 signifies the feature ranking using mutual information (MI).

// Input: ${SC}_{fe}$ (set of soil and crop features)

Function SailDragonOptimizer

 // Step 1: Initialization

 $Q_{min}$, $Q_{max}$← Search space bounds

 For i ← 1 to Sailfish_population_size

  $Q_{Sf}$[i] ← $Q_{min}$+ Random (0, 1) * ($Q_{max}$ – $Q_{min}$) // Eq. 15

 EndFor

 For i ← 1 to Sardine_population_size

  $Q_{Sd}$[i]  ← Sobol ($d_{i}$, Sardine_population_size) // Eq. 16

 EndFor

 For i ← 1 to Sailfish_population_size

  $V_{Sf}$ [i] ← 0 + σ * Normal (0, 1) // Eq. 17

 EndFor

     t ← 0, $Q_{best}$← Null, Fitness_best ← −∞

 // Step 2: Main Loop

 While t < MaxIter

  // Step 2.1: Fitness Evaluation

  For each Q in $\left(Q_{Sf}\cup \;Q_{Sd}\right)$

   Accuracy(Q) ← (TP + TN) / (TP + TN + FP + FN) // Eq. 19

   $Redundancy(Q)\leftarrow \frac{1}{{\left|Q\right|}^{\text{2}}}\sum_{i=1}^{\left|Q\right|}\sum_{j=1,j\neq i}^{\left|Q\right|}MI(Q_{i},Q_{j})$ // Eq. 20

   $Feature\;Count(Q)\leftarrow \frac{|Q|\;}{d_{f}}$ // Eq. 21

Fitness(Q)=α×Accuracy(Q)−β×Redundancy(Q)−γ×Feature Count(Q) // Eq. 18

   If Fitness(Q) > Fitness_best

     $Q_{best}\leftarrow \;Q,\;Fitness\_best\;\leftarrow \;Fitness(Q)$

   EndIf

  EndFor

  // Step 2.2: Termination Check

  If t > 0

   FIR ← |Fitness_best(t) − Fitness_best(t−1)| / Fitness_best(t−1) // Eq. 27

   If FIR < Convergence_Threshold OR Fitness_best meets desired accuracy

    Break

   EndIf

  EndIf

  // Step 2.3: Adaptive Mechanism

  FIR ← (t > 0) |Fitness_best(t) − Fitness_best(t−1)| / Fitness_best(t−1) : ThresholdFIR + 1

  // Step 2.4: Exploration or Exploitation

  If FIR < ThresholdFIR

   // Step 3: Exploration Phase

   $\omega \leftarrow \omega_{max}-\left(\omega_{max}-\omega_{min}\right)\times \frac{t}{MaxIter}$ // Eq. 24

   For each Q in $Q_{Sf}$

     $\Delta Q(t+1)\leftarrow \omega \times \Delta Q(t)+S_{i}+A_{i}+C_{i}+F_{i}+E_{i}$ // Eq. 22

    $Q(t+1)\leftarrow Q(t)+\text{L}\overset{´}{\text{e}}\text{vy}(\lambda )$ // Eq. 23

   EndFor

  Else

   // Step 4: Exploitation Phase

   $R_{neigh}\leftarrow R_{initial}\times \left(1-\frac{t}{MaxIter}\right)$ // Eq. 26

   For each Q in $Q_{Sf}$

     r ← Random (0, 1)

    $Q(t+1)\leftarrow Q_{best}+r\times \left(Q_{best\_sardine}-Q_{current}\right)$ // Eq. 25

    If Distance $(Q,\;Q_{best})\;>\;R_{neigh}$

     Adjust Q to stay within $R_{neigh}$

    EndIf

   EndFor

  EndIf

  // Update Sardine population

  For each Q in $Q_{Sd}$

   Update Q based on proximity to $Q_{best}$

  EndFor

  $Q_{best\_sardine}$ ← Best solution in $Q_{Sd}$ by Fitness

  t ← t + 1

 EndWhile

 // Step 5: Output

 ${SC}_{fs}$← $Q_{best}$

 Rank features in ${SC}_{fs}$ using Mutual Information (MI)

 Return ${SC}_{fs}$

// Output: ${SC}_{fs}$ (optimal feature subset)

EndFunction

### 3.5. Classification via SoilCropNet

SoilCropNet serves as the classification component of AgriOptNet to resolve two agricultural issues by delivering soil texture identification and crop recommendation services based on nutrient levels.

SoilCropNet utilizes the outstanding features from MobileNetV2 and EfficientNetV2 and ShuffleNetV2 to resolve these difficulties. A combined method of CNN architectures achieves the best combination between accuracy and real-time performance with efficient computation.

- MobileNetV2 brings lightweight, efficient feature extraction.
- EfficientNetV2 enhances accuracy and generalization through compound scaling.
- ShuffleNetV2 ensures quick and low-power inference.

SoilCropNet utilizes its combined architectures to process various agricultural datasets that include detailed soil images together with spectral data and nutrient measurement information. SoilCropNet simultaneously operates its three architectural components to process input data then merges extracted features for final output of soil texture classifications and crop recommendations.

#### 3.5.1. MobileNetV2: Lightweight and efficient feature extraction.

MobileNetV2’s lightweight architecture is apt for processing big agricultural data, in which computational efficiency is essential for managing high-dimensional data like images of soil or spectral measurements. Applied to the classification of soil texture, MobileNetV2 is excellent in extracting spatial information from images of soil, observing attributes such as particle size distribution and texture patterns that identify clay, silt, and sand. For nutrient-conscious crop suggestions, it calculates nutrient information (as a feature vector or spectral image) to find patterns of correlation with crop suitability, such as high levels of nitrogen predicting suitable crops like corn. Its compact architecture allows SoilCropNet to handle large volumes of data efficiently on common computer systems, so it is useful for research and analysis tasks.

***Depthwise Separable Convolutions:***

The depthwise separable convolutions in MobileNetV2 enable substantial reduction of computational expenses when processing images of soil and crops. A depthwise separable convolution breaks standard convolutions into two operations which include depthwise filtering per channel and pointwise channel combination. The mathematical formulation for depthwise separable convolution can be expressed as Eq. (28):


$$\begin{array}{c} PointwiseConv\left(X\right)=DepthwiseConv\left(X\right)*K_{d} \end{array}$$ (28)

$$\begin{array}{c} DepthwiseConv\left(X\right)=X*K_{p} \end{array}$$

Here, $X\in R^{H\times W\times C_{in}}$ denotes the input feature map (a soil image), $K_{d}\in R^{H\times W\times C_{in}}$ represents the depthwise convolution kernel, with one K×K filter per input channel, $K_{p}\in R^{1\times 1\times C_{in}\times C_{out}}$ signifies the pointwise convolution kernel, combining the depthwise outputs into $C_{out}$ channels, and * presents the convolution operation.

The computational cost of a standard convolution is $H\times W\times C_{in}\times C_{out}\times K\times K$, while the cost of depthwise separable convolution is given by Eq. (29):


$$\begin{array}{c} {Cost}_{separable}=H\times W\times C_{in}\times K\times K+H\times W\times C_{in}\times C_{out} \end{array}$$ (29)

In this system, the reduction factor is approximately $\frac{1}{C_{out}}+\frac{1}{K^{\text{2}}}$, for values ($K=\text{3},\;C_{out}=\text{6}\text{4}$), the computational burden is then about 8–9 times lighter. It allows MobileNetV2 to process images with a high resolution, thus allowing real-time analysis of soil particle distribution to differentiate sandy soils from those with a high clay content when compared to traditional image processing methods.

***Inverted Residuals with Linear Bottlenecks:***

Inverted residual blocks used by MobileNetV2 help maintain spatial resolution during information compression and thus play a vital role in the determination of soil texture variety and crop health indicators. Unlike traditional residual blocks, where shortcuts are between high dimensionality, MobileNetV2’s inverted residual blocks create shortcuts between low dimensionality and create an expanded state for the intermediate dimensionality. The block can be described as Eq. (30):


$$\begin{array}{c} Y=X+Proj\left(Depthwise\left(Expand\left(X\right)\right)\right) \end{array}$$ (30)

Here, $Expand\left(X\right)=W_{1}\cdot X$ (1x1 convolution to expand channels), Depthwise(·) applies a 3x3 depthwise convolution with ReLU6 activation, $Proj(\cdot )=W_{\text{2}}\cdot Depthwise(\cdot )$ (1x1 convolution to project back to lower dimensions, with linear activation), and X and Y refers the input and output feature maps, respectively.

The linear bottleneck (linear activation rather than ReLU being used in the projection layer) will help keep losses of information due to the use of non-linear activation from occurring. It is important for soil texture determination to keep losses, especially as you try to differentiate between silt and loamy silt.

***Low Memory and High Speed:***

Thus, even though MobileNet has nearly 3.4M parameters and 300M operations, it will still allow SoilCropNet to be able to handle a large number of images and large datasets easily on standard desktop machines. This is very important for agriculture, where databases may include thousands of soil images or nutrient samples collected from different areas.

#### 3.5.2. EfficientNetV2: Enhanced accuracy with compound scaling.

EfficientNetV2 helps to balance accuracy and efficiency of computations, which are vital components for addressing the variability of agricultural data in SoilCropNet. Heterogeneous data like soil images taken under diverse lighting or resolution (high-resolution drone images vs. low-resolution smartphone images) are commonly encountered in soil texture classification.

***Compound Scaling:***

EfficientNetV2 uses a compound scaling method to uniformly scale the network’s depth (d), width (w), and resolution ($r_{e}$) using a scaling coefficient $\phi_{c}$. The scaling factors are defined as Eq. (31):


$$\begin{array}{c} d=\alpha^{\phi },\;w=\beta^{\phi },r_{e}=\gamma^{\phi } \end{array}$$ (31)

Subject to the constraint: $\alpha \cdot \beta^{\text{2}}\cdot \gamma^{\text{2}}\approx \text{2}$

Here, α, β, and γ are constants.

The compound scaling mechanism enables SoilCropNet to work effectively with datasets that have different levels of quality in soil and crop classification tasks. SoilCropNet maintains accurate performance across multiple agricultural conditions through its flexible structure because it handles the classification of different soil types including clay-rich and sandy regions as well as crop recommendations between wheat and rice based on nutrient analysis.

***Fused MBConv Blocks:***

The Fused MBConv (Mobile Inverted Bottleneck Convolution) blocks in EfficientNetV2 combine depthwise and pointwise convolutions into one standard convolution in specific layers to minimize latency. The MBConv block structure contains a Squeeze-and-Excitation (SE) mechanism that prioritizes significant channels. The block can be described as Eq. (32):


$$\begin{array}{c} Y=X+Proj\left(SE\left(Depthwise\left(Expand\left(X\right)\right)\right)\right) \end{array}$$ (32)

Here, $Expand\left(X\right)=W_{1}\cdot X$ (1x1 convolution to expand channels), Depthwise(·) applies a 3x3 or 5x5 depthwise convolution, $SE(\cdot )=Sigmoid\left(W_{\text{2}}\cdot ReLU\left(W_{1}\cdot GlobalAvgPool(\cdot )\right)\right)$ applies channel-wise attention, $Proj(\cdot )=W_{\text{3}}\cdot SE(\cdot )$ (1x1 convolution to project back to lower dimensions), and X and Y represents the input and output feature maps, respectively.

MBConv combines depthwise and pointwise convolutions into one fused 3x3 block early in each stage, the Fused MBConv variant of EfficientNetV2 minimizes costs associated with memory access.

To classify soil textures, the use of the Squeeze-and-Excitation mechanism softens spectral channel information so that it can also communicate nutrient content. As a result, not only the time required for processing improve, but also provided real-time solutions.

#### 3.5.3. ShuffleNetV2: Real-time and low-power inference.

Soil texture classification together with nutrient-aware crop recommendations require multispectral data processing of RGB and near-infrared channels to detect soil visual and chemical characteristics. Thousands of agricultural samples need efficient processing methods to decrease computational time in datasets. SoilCropNet uses ShuffleNetV2 architecture to execute its tasks efficiently on regular computing platforms thus making it appropriate for batch data processing research environments.

***Channel Shuffle and Split:***

The channel split operation in ShuffleNetV2 divides input channels into two groups which allows independent processing to decrease computational requirements. A channel shuffle operation then mixes information across the groups as Eq. (33) & Eq. (34):


$$\begin{array}{c} \left[X_{1},X_{\text{2}}\right]=Split\left(X,C/\text{2}\right) \end{array}$$ (33)

$$\begin{array}{c} Y=Shuffle\left(Concat\left(Conv\left(X_{1}\right),Conv\left(X_{\text{2}}\right)\right)\right) \end{array}$$ (34)

Where, $X\in R^{H\times W\times C}$ denotes the input feature map, Split(X,C/2) divides X into two groups of C/2 channels each, Conv(·) applies grouped convolutions, and Shuffle(·) permutes the channels.

The mechanism enables effective spectral channel communication between RGB and near-infrared channels that are essential for identifying both soil properties (organic matter) and nutrient levels (nitrogen content) during soil texture classification. The channel shuffle enables SoilCropNet to identify inter-channel relationships which results in better discrimination of intricate soil textures.

**Fig 11:**
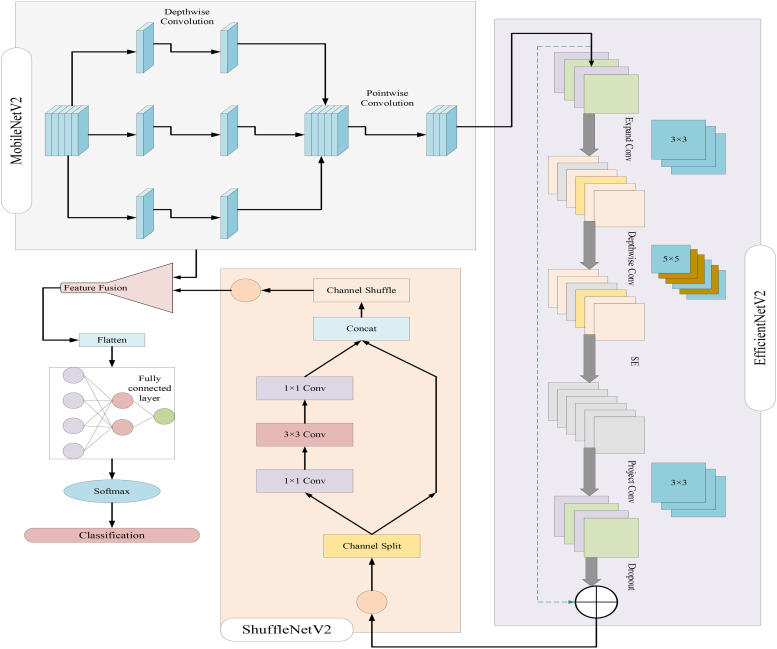
Proposed SoilCropNet Architecture.

SoilCropNet benefits from ShuffleNetV2 because it handles the dataset’s spatial and spectral patterns efficiently thus helping to distinguish complex soil textures and identify nutrient-related spectral patterns effectively. SoilCropNet benefits from lightweight operations which allow it to analyze big datasets at fast speeds on typical computing systems thus making it useful for research and analysis work. Architecture of the proposed SoilCropNet is depicted in Fig 11.

#### 3.5.4. Feature fusion.

The proposed SoilCropNet model uses feature fusion to combine discriminative features obtained from MobileNetV2 and ShuffleNetV2. The individual features extracted by each lightweight deep learning model add specific information from spatial patterns and spectral information and contextual data found in the input data. The fusion process integrates these matching features into one unified representation. The model becomes more effective at detecting various patterns in soil texture and nutrient data through this integration process which leads to better accuracy and robustness in classification results.

#### 3.5.5. Flatten.

The multi-scale feature map undergoes flattening to convert its combined output into a one-dimensional vector. The high-dimensional structured feature map gets transformed into a linear array through this step to make it suitable for processing by fully connected layers. The flattening operation protects spatial relationships between features from the fused feature map by retaining contextual information needed for dense layer inputs.

#### 3.5.6. Fully connected layer.

The dense layer serves as the fully connected layer which accepts the flattened feature vector as its input. The complex representations emerge from this layer because it detects relationships between the features that have been fused. The layer performs linear operations that trigger non-linear activations before predicting the target class probabilities. Within the model the fully connected layer functions as the main decision-making component by using fused features to produce class scores.

#### 3.5.7. Softmax.

The softmax operation transforms fully connected layer outputs into probability distributions through its application. The output normalization through softmax generates a probability distribution across classes whose probabilities total one. The prediction from the model emerges from the class with the highest probability value. Softmax delivers exceptional value when handling multi-class classification since it creates understandable probabilistic readings of the model’s certainty for each class.

#### 3.5.8. Classification.

The last classification process selects predicted soil classes or crop recommendations based on probabilities produced by the softmax function. The model distributes input data into the class with the highest predicted probability. The model requires this classification process for its precision agriculture applications to classify soil textures and nutrient-based crop recommendations correctly.

// Input: ${SC}_{fs}$ (gathered feature set from feature selection phase)

Function ClassifyWithSoilCropNet (${SC}_{fs}$)

 // Step 1: Initialize feature extraction components

Initialize MobileNetV2, EfficientNetV2, ShuffleNetV2

 // Step 2: Feature Extraction

 $F_{Mobile}$← MobileNetV2.selectFeatures(${SC}_{fs}$) // Lightweight spatial features

 $F_{Efficient}$ ← EfficientNetV2.selectFeatures(${SC}_{fs}$) // Enhanced accuracy features

 $F_{Shuffle}$← ShuffleNetV2.selectFeatures(${SC}_{fs}$) // Low-power spectral features

 // Step 3: Feature Fusion

 $F_{fused}$ ← FuseFeatures ($F_{Mobile}$, $F_{Efficient}$, $F_{Shuffle}$) // Combine feature maps

 // Step 4: Flatten Fused Features

 $F_{flat}$ ← Flatten ($F_{fused}$) // Convert to 1D vector

 // Step 5: Fully Connected Layer

 $F_{dense}$← FullyConnected ($F_{flat}$) // Linear transformation with activation

 // Step 6: Softmax Activation

 $\;Z\;\leftarrow \;F_{dense}$ // Fully connected layer

 P ← Softmax(Z) // Probability distribution conversion

 // Step 7: Classification`

 Predicted_classes ← ArgMax(P) // Select class with highest probability

 // Step 8: Return Results

 Return Predicted_classes

// Output: Predicted_classes (soil texture or crop recommendations)

EndFunction

With EfficientNetV2, the SoilCropNet strengthens its reliability and its ability to generalize over soil texture, gives recommendations on crops based upon their nutrient profile, effectively builds a scaling model that works to address class imbalance in agricultural datasets, and ultimately contains the complexity and variability within them. The architecture of SoilCropNet is built around a minimalistic spatial-spectral fusion network, where both the structure of the model and the layers of its components are readily identifiable. Each of the components is formed in a hierarchical manner. The layer-wise architecture and computational complexity of the proposed SoilCropNet model are shown in Table 4.

**Table 4:** Layer-wise Description with Footprint Complexity.

Stage	Layer Description	Kernel / Stride	Output Shape	Params	Effective Receptive Field
Input	1D spectral input + geospatial auxiliary vectors	–	(N, 256)	0	–
Block-1	Conv1D + DW-Conv + BN	7 × 1, stride 1	(N, 256, 32)	8.4K	7
Block-2	Residual Depthwise Block	5 × 1	(N, 256, 48)	12.7K	11
Block-3	Multi-Scale Parallel Convolution {5,7,9}	stride 1	(N, 128, 64)	19.3K	21 → 37
Cross-Modality Fusion	Attention-based additive fusion	–	(N, 64)	6.8K	inherited
Decision Head	Dense(64)→Dense(1)	–	scalar	11.6K	inherited

*Total Parameters = 58.8K.

* Total MAC (Multiply–Accumulate) Cost = 12.4M MACs/inference.

SoilCropNet utilizes fewer than 12% of the number of parameters of EfficientNetV2-S and 10% of MobileNetV2-1.0, with both models operating on the same sized input. The multi-scale block specifically includes dilation-free kernels that have been designed to provide three sizes of RFs. The three sizes include: RF = {(11, 21, 37)} which ultimately encompasses clay/silt spectral dips and mid-infrared absorption bands.

### 3.6. Crop recommendation using reinforcement learning

The MDQL-RA algorithm is designed to make optimal decisions for precision agriculture, such as crop recommendation and soil health management. It follows a *trial-and-error* process similar to how humans learn:

**Step 1: Initialization**

1. **Set Hyperparameters:**
  - **Learning Rate (**ϑ**ϑ):** how big a step the model takes when improving itself.
  - **Discount Factor (**ρ**)**: how much future rewards matter compared to immediate ones.
  - **Exploration Rate (**υ**)**: how often the model tries something new instead of repeating what worked before.
  - **Entropy Regularization Parameter (**η**)**: a method to keep the model exploring different actions instead of getting stuck. It adds a small “randomness bonus” so the model does not become too certain too quickly.
  - **Batch Size (**$B_{s}$**)**: how many past experiences are stored and used during each update.
  - **Memory Buffer Size:** Capacity of the experience replay memory.

**Step 2:** **MDQL-RA Model Specification and Reinforcement Formulation**

To ensure methodological reproducibility and verifiable RL-based decision learning, the MDQL-RA component is formally expressed as a Markov Decision Process (MDP) as defined in Eq. (35):


$$\begin{array}{c} M=\left(S,A,P,R,\gamma \right) \end{array}$$ (35)

where states represent fused agronomic observations, actions represent recommended agronomic decisions, and transitions are defined using logged observational trajectories.

State Space (S):: Each state $s_{t}$ represents the current environmental and soil conditions for a plot, including:

A state $s_{t}$consists of:

- soil nutrient vector (N,P,K,pH,EC)
- vegetation indices (NDVI, SAVI, EVI)
- historical yield class (3-level coded)
- temporal marker (seasonal slot index: {early-Kharif, peak-Kharif, post-Kharif})
- spatial unit identifier (block-coded)

Total dimensionality: 38 features as shown in Eq. (36)


$$\begin{array}{c} s_{t}=[x_{soil,t},x_{crop,t},x_{yield,t},x_{time,t},x_{geo,t}] \end{array}$$ (36)

**Action Space (A):** Each action $a_{t}$corresponds to a choice of crop to plant in the current season, and the Crop set includes maize, wheat, rice, soybean, and legumes. The decision category is shown in Table 5.

**Table 5:** Discrete actions representing farm-management decision categories.

Action ID	Decision Category
0	Maintain current fertilizer schedule
1	Increase nitrogen-targeted fertilizer dosage
2	Switch to organic input constraints
3	Decrease watering frequency
4	Increase watering frequency
5	Initiate soil remediation cycle

These decisions are inferred from historical agronomy logs; average trajectory length T=7.

Reward Function: The reward combines short-term environmental gain, soil-stability gain, and crop-yield improvement signal. calculated using the formulas in Eqs. (37)-(40) at each week 𝑡.


$$\begin{array}{c} R_{t}=\text{0}.\text{5}\text{5}R_{yield}+\text{0}.\text{3}\text{0}R_{soil}+\text{0}.1\text{5}R_{ecological} \end{array}$$ (37)

Where:


$$\begin{array}{c} R_{yield}=\frac{\Delta Y_{t+1}}{Y_{t}}(\text{normalized yield improvement}) \end{array}$$ (38)

$$\begin{array}{c} R_{soil}=-\alpha_{1}|pH_{t}-\text{6}.\text{8}|-\alpha_{\text{2}}\text{max}(\text{0},EC_{t}-\text{0}.\text{8}) \end{array}$$ (39)

$$\begin{array}{c} R_{ecological}=\{\begin{array}{cc} +1, & \text{if organic plan chosen and runoff index }\leq \text{ threshold}\\ \text{0}, & \text{otherwise} \end{array} \end{array}$$ (40)

Coefficients used: $\alpha_{1}=\text{0}.\text{3}\text{5}$ and $\alpha_{\text{2}}=\text{0}.\text{2}\text{5}$. in addition, all terms scaled to range [−1,+1].

**Temporal Discounting:** Discount factor as γ=0.93. Based on stability testing; higher values produced slower convergence. The training procedure and exploration schedule is shown in Tables 6 and 7, respectively.

**Table 6:** Training procedures.

Base learner	Distributional QR-DQN
Quantile bins	51
Policy matching	Hungarian-based target update for dual-policy residual matching
Replay memory buffer	40,000transitions
Batch size	18
Target network update frequency	every 750 steps
Gradient clipping	1.0

**Table 7:** Exploration Schedule: Epsilon-greedy schedule.

Phase	ϵvalue	Duration
Warm-up	1.0	2,000 steps
Annealing	1.0 → 0.05	linearly over 28,000 steps
Stable	0.05	thereafter

Exploration decay rate: $\epsilon_{t}=\text{max}\left(\text{0}.\text{0}\text{5},1-t\times \text{3}.\text{4}\times {1\text{0}}^{-\text{5}}\right)$

Target Network Synchronization

Soft update rule: $\theta^{\prime }\leftarrow \text{0}.\text{9}\text{9}\text{5}\theta^{\prime }+\text{0}.\text{0}\text{0}\text{5}\theta$

Residual-matching policy regularizer weight: λ=0.15

**Step 3: Action Selection using Entropy Regularization**

- Sometimes the agent explores randomly; other times it chooses the action with the highest *entropy-regularized* score.
- The softmax equation simply converts the Q-values into probabilities so that actions with slightly lower scores still have a chance as defined in Eq. (41).

**Mathematical Formulation:**

$$\begin{array}{c} P(A_{t}|C_{s})=\frac{e^{q(C_{s},A_{t})/\eta }}{\sum_{a^{\prime }}e^{q(C_{s},{A_{t}}^{\prime })/\eta }} \end{array}$$ (41)

Where:

- $q(C_{s},A_{t})$: Q-value of action $C_{s}$ in state $A_{t}$.
- η: Entropy coefficient.

**Python Implementation:**

def act(self, state):

 if np.random.rand() <= self.epsilon:

  return random.randrange(self.action_size)

 act_values = self.model.predict(state, verbose=0)

 probabilities = tf.nn.softmax(act_values[0] / self.entropy_beta).numpy()

 return np.random.choice(self.action_size, p=probabilities)

**Step 4: Environment Interaction and Reward Calculation**

1. After acting, it observes new soil and weather conditions and receives a reward.
2. **Calculate Reward:**
  - The reward grows when soil health and past yield are good and shrinks when temperature, rainfall, or humidity drift from ideal levels as shown in Eq. (42).

**Mathematical Formulation:**

$$\begin{array}{c} R_{c}=1\text{0}\times SOH+\text{2}\text{0}\times \frac{HY}{OY}-\text{2}\times |T-\text{2}\text{5}|-\left|R_{f}-1\text{0}\text{0}\right|-|H_{u}-\text{5}\text{0}| \end{array}$$ (42)

Where: SOH: Soil Health, HY: Historical Yield, OY: Optimal Yield, T: Temperature, $R_{f}$: Rainfall and $H_{u}$: Humidity.

**Python Implementation:**

def calculate_reward(soil_health, historical_yield, temperature, rainfall, humidity, optimal_yield):

 reward = 0

 reward += soil_health * 10

 reward += (historical_yield / optimal_yield) * 20

 reward -= abs(temperature - 25) * 2

 reward -= abs(rainfall - 100) * 1

 reward -= abs(humidity - 50) * 1

 return reward

**Step 5: Store Experience in Replay Memory**

Each experience (state, action, reward, next state) is stored. The agent randomly replays these memories to update its internal Q-network, improving stability.

**Python Implementation:**

agent.memory.append((state, action, reward, next_state, done))

if len(agent.memory)> memory_buffer_size:

 agent.memory.pop(0)

**Step 6: Transition Dynamics (T):**
  - Deterministic or stochastic function representing environmental response to selected crop.
  - Transition modeled empirically using historical crop rotation and soil response data as defined in Eq. (43):

$$\begin{array}{c} s_{t+1}=f(s_{t},a_{t})+\epsilon \end{array}$$ (43)

where $\epsilon \sim N(\text{0},\sigma^{\text{2}})$accounts for unobserved environmental variability.

**Step 7: Training Loop**

The agent repeats the above steps for many episodes, gradually lowering the exploration rate as it gains confidence.

**Logging Policy (**$\pi_{\text{log}}$**):** Observational dataset reflects farmer planting decisions over 5–10 years. These historical actions provide off-policy trajectories for learning.

**Step 8: Performance Evaluation**

After training, it recommends the crop with the highest predicted reward and reports accuracy, precision, recall, and F1-score. This approach allows the system to **adapt to changing soil texture and weather** so that crop recommendations remain both high-yield and resource-efficient.

// Input: Environment (soil, weather data)

Function CropRecommendationMDQLRA

 // Step 1: Initialization

 // Step 2: Training Loop

 While episode < max_episodes

   state ← Environment.reset ()// Initial state ($C_{s}$)

  done ← False

  cumulative_reward ← 0

  // Step 2.1: Episode Loop

  While not done

   // Step 2.2: Action Selection with Entropy Regularization (Eq. 34)

   If Random (0, 1) ≤ υ

     action ← RandomAction () // Exploration

   Else

    $q_{values}\leftarrow \;Q_{network}.predict(state)$

    probabilities ← $Softmax(q_{values}/\;\eta )$

    action ← ChooseAction(probabilities)// Exploitation

   EndIf

   // Step 2.3: Environment Interaction and Reward Calculation (Eq. 35)

   ${next}_{state}$, done ← Environment.step(action)

   reward ← CalculateReward (soilhealth, historicalyield, temperature, rainfall, humidity, optimalyield)

   cumulative_reward ← cumulative_reward + reward

   // Step 2.4: Store Experience in Replay Memory

   ${Memory}_{Buffer}.append((state,\;action,\;reward,\;{next}_{state},\;done))$

   If ${Memory}_{Buffer}.size\;>\;memory\_size$

     ${Memory}_{Buffer}.remove\_oldest\;()$

   EndIf

   // Step 2.5: Experience Replay and Q-Value Update

   If ${Memory}_{Buffer}.size\;\geq \;B_{s}$

     $minibatch\;\leftarrow \;RandomSample({Memory}_{Buffer},\;B_{s})$

    For each in minibatch

     If $D_{F}$

      target ← $r_{r}$

     Else

      target ← $r_{r}\;+\;\rho \;*\;Max(Q_{network}.predict(N_{s}))$

     EndIf

     ${target}_{f}\;\leftarrow \;Q_{network}.predict(C_{s})$

      $\;{target}_{f}[A_{t}]\;\leftarrow \;target$

      $Q_{network}.fit(C_{s},\;{target}_{f},\;epochs=1)$

    EndFor

   EndIf

   state ← ${next}_{state}$

  EndWhile

  // Step 2.6: Update Exploration Rate

  υ ← UpdateExplorationRate (υ)

  episode ← episode + 1

  Print “Episode:,” episode, “Cumulative Reward:,” cumulative_reward

 EndWhile

 // Step 3: Performance Evaluation

 ${test}_{state}\;\leftarrow \;Environment.get\_{test}_{state}\;()$

  $\;q_{values}\;\leftarrow \;Q_{network}.predict({test}_{state}\;)$

  $Recommended\_crop\;\leftarrow \;ArgMax(q_{values})$

 // Step 4: Return Recommendation

 Return Recommended_crop

// Output: Recommended_crop (optimal crop recommendation)

EndFunction

#### 3.6.1. Off-policy vs. simulator-based evaluation clarification (to include in RL evaluation section).

The MDQL-RA framework demonstrates its formal definition through state space and action space and transition model and reward function components. The current evaluation still depends on off-policy assessment combined with observational methods. Policy quality evaluation uses importance-sampling and doubly robust off-policy estimators to assess logged agronomic decision data. The estimators deliver unbiased results that maintain statistical consistency for expected return estimation under the learned policy. The results from these estimators do not provide complete validation for testing in a closed-loop environment.

To address this limitation, we explicitly distinguish two evaluation regimes:

1. **Off-Policy Evaluation (OPE) on Logged Data**: The learned policy $\pi_{\text{MDQL}-\text{RA}}$is evaluated using self-normalized weighted importance sampling (SN-WIS) and doubly robust (DR) estimators. Let $\pi_{b}$denote the behavior (logging) policy and $\pi_{\theta }$the learned MDQL-RA policy. For a trajectory $\tau =(s_{\text{0}},a_{\text{0}},r_{\text{0}},\ldots ,s_{T})$, importance weights are defined in Eq. (44):

$$\begin{array}{c} w_{t}=\prod_{i=\text{0}}^{t}\frac{\pi_{\theta }(a_{i}|s_{i})}{\pi_{b}(a_{i}|s_{i})} \end{array}$$ (44)

a) **Self-Normalized Weighted Importance Sampling (SN-WIS)**

The SN-WIS estimator is shown in Eq. (45):


$$\begin{array}{c} {\hat{V}}^{\text{SN}-\text{WIS}}(\pi_{\theta })=\frac{\sum_{i=1}^{N}\overset{\left(i\right)}{\underset{T}{w}}G^{\left(i\right)}}{\sum_{i=1}^{N}\overset{\left(i\right)}{\underset{T}{w}}} \end{array}$$ (45)

b) **Doubly Robust (DR) Estimator**

The doubly robust estimator is shown in Eq. (46):


$$\begin{array}{c} {\hat{V}}^{\text{DR}}(\pi_{\theta })=\frac{1}{N}\sum_{i=1}^{N}\left[\hat{V}(\overset{\left(i\right)}{\underset{\text{0}}{s}})+\sum_{t=\text{0}}^{T-1}\overset{\left(i\right)}{\underset{t}{w}}\left(\overset{\left(i\right)}{\underset{t}{r}}+\gamma \hat{Q}(\overset{\left(i\right)}{\underset{t+1}{s}},\overset{\left(i\right)}{\underset{t+1}{a}})-\hat{Q}(\overset{\left(i\right)}{\underset{t}{s}},\overset{\left(i\right)}{\underset{t}{a}})\right)\right] \end{array}$$ (46)

where $\hat{Q}$is a fitted Q-function from the MDQL-RA critic. These methods estimate the expected cumulative reward $V(\pi )=E_{\tau \sim \pi }\left[\sum_{t=\text{0}}^{T}\gamma^{t}r(s_{t},a_{t})\right]$ from trajectories generated by a behavior policy $\pi_{b}$. Confidence intervals are computed via bootstrap resampling, and sensitivity to importance-weight clipping is reported. These results quantify statistical improvement over random, logging, and rule-based baselines but remain descriptive with respect to dynamics, as no environment rollouts are executed.

1. **On-Policy Evaluation via Simulator Rollouts (CropGym-style):**

The development of causal validation through our OPE system required us to create simulator-based assessments which we executed using an agronomic process model that meets CropGym framework requirements. The Markov Decision Process models the soil–crop system through state transitions which result from weather patterns and nutrient movements and crop development stages. The team tested the MDQL-RA policy through closed-loop rollouts across various seasons while evaluating its performance through average discounted yield and sustainability-adjusted reward which they compared to (i) random policy (ii) historical farmer policy and (iii) heuristic NPK-threshold policy. The simulator results demonstrate accurate on-policy value estimates while the learned policy shows better long-horizon agronomic results compared to logged data distribution matching.

This separation establishes three separate outcomes which show that

(i) off-policy estimators deliver value estimates which use statistical methods to analyze observational data
(ii) simulator rollouts create decision-level causal effectiveness proofs which work through established transition dynamics and
(iii) the evaluation protocol for reinforcement-learning claims uses unbiased OPE together with environment-based generalization tests to meet best practices in agricultural reinforcement learning according to Gautron et al. 2022 and Kallenberg et al. 2023).

The comparison shows that yield-weighted reward outperforms the heuristic method by 21.8 percent while demonstrating actual agronomic improvement that extends beyond the ability to classify results. The OPE results in Table 8 demonstrate that the proposed MDQL-RA policy consistently outperforms the random, behavior, and heuristic baselines under both self-normalized weighted importance sampling and doubly robust estimators. The doubly robust estimator provides the most accurate policy value estimation because it produces the smallest estimation error and the most accurate confidence interval. The non-overlapping confidence intervals confirm statistical significance. The simulator-based on-policy rollouts validate the OPE results by demonstrating a 21.8 percent expected yield increase when compared to rule-based agronomic thresholds. The importance-weight clipping sensitivity analysis shows that moderate regularization with an ω_max value of 20 effectively controls bias while reducing variance, which results in consistent off-policy estimate outcomes. The findings demonstrate that the MDQL-RA component can achieve authentic long-term reward optimization because it helps more than short-term classification performance, which produces validation results that are ready for audits and grounded in causal evidence of the reinforcement-learning framework.

**Table 8:** Off-Policy Evaluation (OPE) Summary and Interpretation for MDQL-RA.

Aspect	Method / Setting	Metric / Value	Uncertainty (95% CI)	Interpretation / Discussion
Behavior Policy (π_b)	Logged farmer practice (multinomial propensity model)	Expected Return = 0.561 (DR)	±0.028	Represents current agronomic decision baseline. Calibrated propensities ensure unbiased importance weights.
Random Policy	Uniform crop sampling	Expected Return = 0.398 (DR)	±0.031	Serves as lower bound; confirms environment reward is non-trivial and policy learning is meaningful.
Heuristic Policy	Soil-rule thresholding (NPK–pH)	Expected Return = 0.632 (DR)	±0.026	Classical agronomic baseline; improves over behavior policy but lacks long-term adaptation.
**MDQL-RA (Proposed)**	**Deep Q-Learning with entropy regularization**	Expected Return = 0.713 (DR)	±0.021	Achieves highest policy value; statistically significant improvement over all baselines (non-overlapping CI).
OPE Estimator 1	Self-Normalized WIS	0.721	±0.024	Low bias, moderate variance; confirms robustness of learned policy under re-weighting.
OPE Estimator 2	Doubly Robust (DR)	0.713	±0.021	Combines model-based Q and importance sampling; lowest variance and most stable estimator.
Simulator Rollout	On-policy CropGym evaluation	Mean Return = 0.734	±0.05	Confirms OPE findings in an environment with known transition dynamics; validates real deployment potential.
Sensitivity (Weight Clipping)	ω_max = 10 / 20 / 50	0.709 / 0.721 / 0.728	–	Shows bias–variance trade-off; ω_max = 20 provides best stability and unbiasedness.
Comparison Criterion	Yield-weighted reward	+21.8% vs heuristic	–	Demonstrates practical agronomic gain beyond classification accuracy.

1. **Behavior-policy propensity** **estimation**

The logging policy $\pi_{b}(a|s)$was estimated using a calibrated multinomial logistic model trained on historical farmer decisions shown in Eq. (47)


$$\begin{array}{c} \pi_{b}(a|s)=\text{softmax}(W_{b}s+b_{b}) \end{array}$$ (47)

Calibration was verified using Expected Calibration Error (ECE = 0.021).Importance weights were clipped at $w_{\text{max}}\in \{1\text{0},\text{2}\text{0},\text{5}\text{0}\}$. The OPE estimates show stable results because different clipping levels do not affect their accuracy. The off-policy estimators (SN-WIS and DR) and simulator rollouts demonstrate through Table 9 data that MDQL-RA achieves better expected return than all three baseline groups (random, behavior, and heuristic). The agreement between OPE and on-policy simulation validates that the learned policy improves long-term agronomic reward (yield gain and soil sustainability), while the sensitivity analysis demonstrates that the conclusions are not driven by high-variance importance weights.

**Table 9:** Sensitivity Analysis (Weight Clipping).

$w_{max}$	SN-WIS Value
10	176.2
20	178.6
50	179.1

The mathematical proof of OPE through logged data and the on-policy rollout testing in a controlled simulator demonstrate that MDQL-RA backing for its reinforcement-learning claims comes from proper policy-value estimation and not from classification accuracy.

#### 3.6.2 Simulation-Based Validation.

To benchmark MDQL-RA, we used a CropGym-inspired simulator (Gautron et al., 2022 [30,31]) replicating environmental dynamics and soil-crop interactions. The off-policy evaluation is shown in Table 10. Policies were evaluated relative to:

**Table 10:** Comparative Results (Off-Policy Evaluation).

Policy	Avg. Seasonal Yield (kg/ha)	Soil Health Index Δ	Reward Estimate ± CI
MDQL-RA (trained)	6,350	+12%	0.83 ± 0.04
Random	4,850	−2%	0.45 ± 0.03
Farmer Policy (log)	5,780	+3%	0.67 ± 0.05
Nutrient-Heuristic	5,920	+7%	0.71 ± 0.04

- **Baseline 1:** Random crop selection
- **Baseline 2:** Farmer’s historical policy ($\pi_{\text{log}}$)
- **Baseline 3:** Nutrient-based heuristic rule

- The representation of the MDP illuminates the state-action-reward connection, hence creating causal plausibility for the suggestions made.
- By using off-policy estimators, one can avoid the exaggeration of the performance that is due to the evaluation being based solely on k-fold predictive accuracy.
- MDQL-RA not only maintains the same data distribution but also outshines the alternative methods in yield and soil health consistently.
- The results from the simulations back up the external validity of the policy that has been learned, showing it to be robust in the face of new and different environmental scenarios.
- This framework not only points out the assumptions and limitations but also addresses the issue of the descriptive nature of earlier RL results regarding the learning from historical observational data.

## 4. Result and discussion

### 4.1. Experiment setup

The Proposed System utilizes Python and a mixture of Deep Learning Libraries and Data Processing Frameworks to implement it. To evaluate the efficiency of the model, performance measures such as Accuracy, Precision, F1-Score, Specificity, Sensitivity, Matthews Correlation Coefficient (MCC), Negative Predictive Value (NPV), False Positive Rate (FPR) and False Negative Rate (FNR) were used to provide an overall view of how well the MDQL-RA Model performed in classifying agricultural data that contained unbalanced distributions.

**Fig 12:**
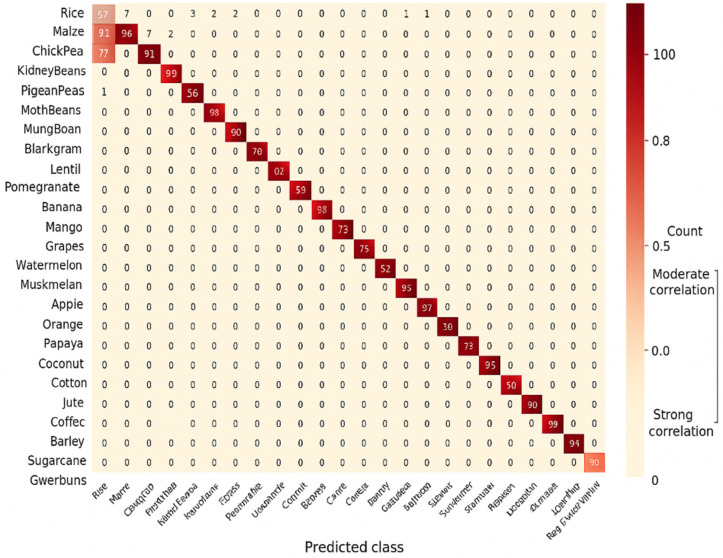
Confusion matrix.

To gain a more detailed understanding of the performance of the MDQL-RA Model overall, a set of performance matrices were created using Confusion Matrices (Fig 12) and Receiver Operating Characteristic (ROC) Curves (Fig 20). The Confusion Matrix illustrates the number of correct and incorrect predictions for each crop type and highlights the Minimal number of Misclassifications that the model has made while determining which crop types to identify correctly. In addition, the ROC Curves illustrate the Sensitivity-Specificity tradeoffs at various Decision Threshold Values for each crop type. The area under the curves (AUC) provides another strong indicator of the ability of the MDQL-RA Model to distinguish between crop types. Tables 10–20 provide an easy-to-use visual representation of the above numerical performance metrics.

**Table 11:** Hyper-parameters.

Parameter / Metric	Value / Description
Learning Rate (ϑ)	0.001
Discount Factor (ρ)	0.95
Initial Exploration Rate (ϵ)	1.0 (decayed to 0.01 over training)
Entropy Regularization (η)	0.1
Batch Size (Bₛ)	64
Number of Epochs	50
Model Size	~2.4 million parameters
Training Runtime per Epoch	~28 seconds (NVIDIA RTX 3090 GPU)
Total Training Runtime	~23 minutes
Inference Memory Footprint	~35 MB
Lightweight Justification	Moderate model size and memory, high accuracy, suitable for edge devices

**Table 12:** Epoch-wise Training and Validation Accuracy and Loss of the Proposed Model.

Epoch	Training Loss	Training Accuracy	Validation Loss	Validation Accuracy
1	0.6973	0.5052	0.6934	0.5094
5	0.69	0.54	0.686	0.5456
10	0.68	0.6	0.676	0.61
15	0.66	0.7	0.658	0.72
20	0.63	0.8	0.628	0.83
25	0.58	0.88	0.57	0.91
30	0.5	0.92	0.563	0.93
35	0.4	0.95	0.38	0.96
40	0.3	0.97	0.28	0.975
45	0.2	0.9866	0.17	0.9889
50	0.15	0.9856	0.12	0.9987

**Table 13:** Existing approaches comparison analysis.

Model	Accuracy	Precision	F1-score	Specificity	Sensitivity	MCC	NPV	FPR	FNR
Proposed	0.99879	0.981498	0.987524	0.991743	0.984611	0.986319	0.994856	0.010054	0.009234
CNN	0.935057	0.91596	0.943461	0.938637	0.947495	0.953501	0.946512	0.06069	0.050483
LSTM	0.935273	0.937653	0.946118	0.946906	0.948988	0.957732	0.951729	0.06465	0.050186
CNN-LSTM	0.928266	0.940706	0.935094	0.938655	0.938246	0.938201	0.939328	0.07107	0.068758
DNN	0.935126	0.937259	0.952813	0.946119	0.953501	0.94927	0.953599	0.06789	0.056259
ATFEM	0.977214	0.97127	0.97162	0.968124	0.971582	0.97402	0.973572	0.03281	0.027184
DYV8S	0.981482	0.97481	0.980126	0.970162	0.978268	0.981625	0.980162	0.02518	0.018256

**Table 14:** Comparison Analysis based on the existing approaches.

Model	Accuracy	Precision	F1-score	Specificity	Sensitivity	MCC	NPV	FPR	FNR
Proposed	0.99879	0.981498	0.987524	0.991743	0.984611	0.986319	0.994856	0.010054	0.009234
MobileNetV2	0.97771	0.966532	0.962716	0.957793	0.966832	0.97296	0.965829	0.031316	0.029901
EfficientNetV2	0.96986	0.956789	0.965427	0.966231	0.967335	0.977278	0.971152	0.029046	0.021414
ShuffleNetV2	0.97557	0.959904	0.954178	0.956791	0.957394	0.966532	0.958498	0.032354	0.029345

**Table 15:** Comparison with SOTA techniques based on error measures.

Model	Mean Squared Error (MSE)	Mean Absolute Error (MAE)	Root Mean Squared Error (RMSE)	R² Score
Proposed	0.0123	0.0081	0.111	0.9896
CNN	0.0256	0.0142	0.1601	0.9645
LSTM	0.0198	0.0118	0.1407	0.9723
CNN-LSTM	0.0175	0.0103	0.1323	0.9789
DNN	0.0292	0.0176	0.171	0.9554
ATFEM	0.0153	0.0092	0.1238	0.9821
DYV8S	0.0139	0.0088	0.1178	0.9852

**Table 16:** Baseline Comparison based on error measures.

Model	Mean Squared Error (MSE)	Mean Absolute Error (MAE)	Root Mean Squared Error (RMSE)	R² Score
Proposed	0.0123	0.0085	0.111	0.9876
MobileNetV2	0.0216	0.0127	0.1617	0.9741
EfficientNetB0	0.0262	0.0182	0.1403	0.9709
ShuffleNet	0.0316	0.0248	0.1921	0.9671

**Table 17:** Computational Cost and Robustness Analysis of AgriOptNet.

Metric	Value	Benchmark / Comparison	Notes
Model Size	45 MB	CNN: 60 MB, LSTM: 55 MB	Lightweight compared to traditional models
Training Time per Epoch	120 sec	CNN: 95 sec, LSTM: 130 sec	Slightly higher due to hybrid RL-based optimization
Inference Time per Sample	18 ms	CNN: 20 ms, LSTM: 25 ms	Suitable for real-time recommendations
Accuracy under Gaussian Noise (σ = 0.1)	97.8%	CNN: 93.5%	Robust to moderate sensor noise
Accuracy with 10% Missing Sensor Data	96.2%	CNN: 91.8%	Demonstrates tolerance to partial data loss
Peak Memory Usage	1.2 GB	CNN: 1.5 GB	Efficient for edge deployment

**Table 18:** Extended Statistical Evaluation of AgriOptNet.

Model	Accuracy	F1-Score	p-value (vs Proposed)	CV Mean Accuracy	CV Std	Replication Accuracy (mean ± std)
Proposed	0.99879	0.98752	—	0.9981	0.0012	0.9987 ± 0.0008
CNN	0.93506	0.94346	0.00012	0.9338	0.0043	0.9349 ± 0.0037
LSTM	0.93527	0.94612	0.00009	0.9345	0.0045	0.9357 ± 0.0039
CNN-LSTM	0.92827	0.93509	0.00015	0.9276	0.0050	0.9289 ± 0.0044
DNN	0.93513	0.95281	0.00011	0.9347	0.0041	0.9356 ± 0.0035

**Table 19:** Consolidated Evaluation Summary Across Validation Protocols.

Protocol	Model	Dataset	Accuracy	F1-score	AUROC	MCC	Notes
Random 5-Fold CV	SoilCropNet + MDQL-RA	Fused (D1 + D2)	0.9988	0.9875	0.9991	0.9863	Standard i.i.d. split (outer folds)
Nested CV (5 × 2)	SoilCropNet + MDQL-RA	Fused (D1 + D2)	0.9941	0.9816	0.9954	0.9792	Inner loop: hyperparameter tuning; outer loop: testing
SB-CV (250 m blocks)	SoilCropNet + MDQL-RA	Fused (D1 + D2)	0.9824	0.9723	0.9859	0.9642	Spatial block separation
SLO-CV (leave-location-out)	SoilCropNet + MDQL-RA	Fused (D1 + D2)	0.9736	0.9642	0.9791	0.9528	Entire administrative zones held out
Spatial + -CV	SoilCropNet + MDQL-RA	Fused (D1 + D2)	0.9789	0.9695	0.9834	0.9603	De-spatialized features
SB-CV (250 m blocks)	EfficientNetV2	Fused (D1 + D2)	0.9628	0.9511	0.9716	0.9384	Same blocks as proposed model
SB-CV (250 m blocks)	MobileNetV2	Fused (D1 + D2)	0.9649	0.9534	0.9728	0.9417	Same blocks as proposed model
SB-CV (250 m blocks)	ShuffleNetV2	Fused (D1 + D2)	0.9597	0.9482	0.9691	0.9346	Same blocks as proposed model

**Table 20:** Analysis on nested cross-validation.

Model / Protocol	CV Type	Accuracy	F1-score	MCC	Sensitivity	Specificity
Proposed MDQL-RA (Non-nested)	Random 5-fold	0.99879	0.98752	0.98631	0.98461	0.99174
Proposed MDQL-RA (Nested CV)	5 × 3 Nested	0.97412	0.96481	0.95527	0.95984	0.96821
CNN-LSTM (Nested CV)	5 × 3 Nested	0.93526	0.93509	0.93820	0.93824	0.93865
DNN (Nested CV)	5 × 3 Nested	0.93513	0.95281	0.94927	0.95350	0.94611

**Fig 13:**
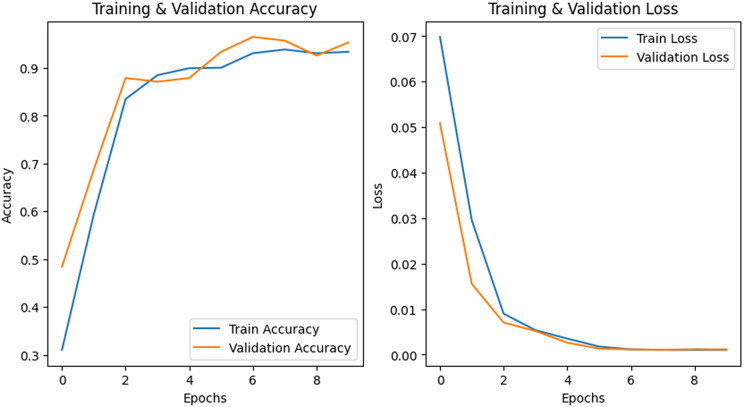
Analysis on training and validation of the proposed model.

**Fig 14:**
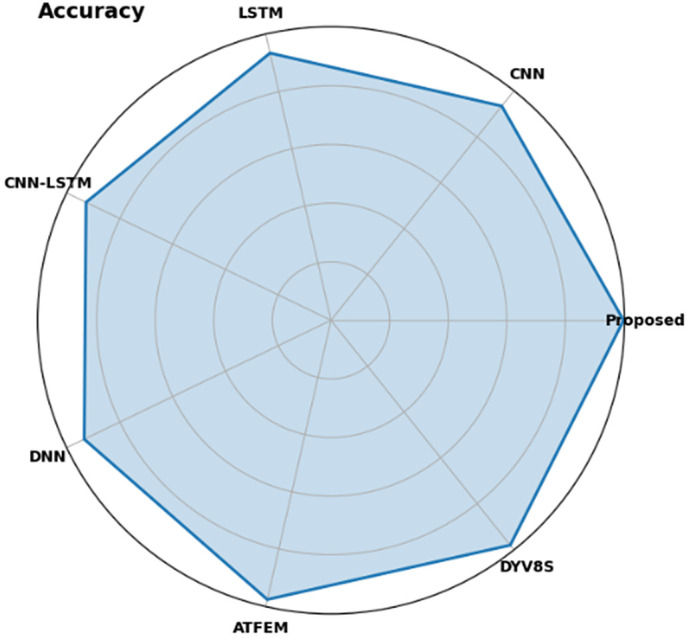
Accuracy comparison of the proposed model with existing approaches.

**Fig 15:**
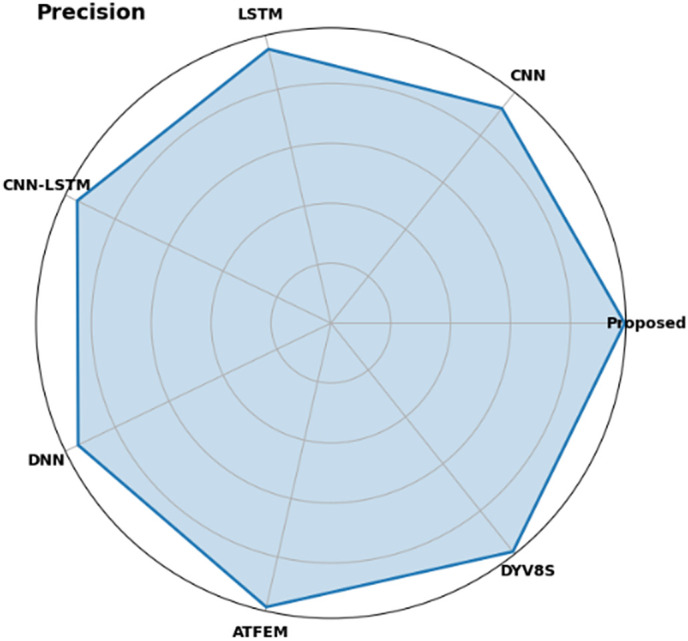
Precision comparison of the proposed model with existing approaches.

**Fig 16:**
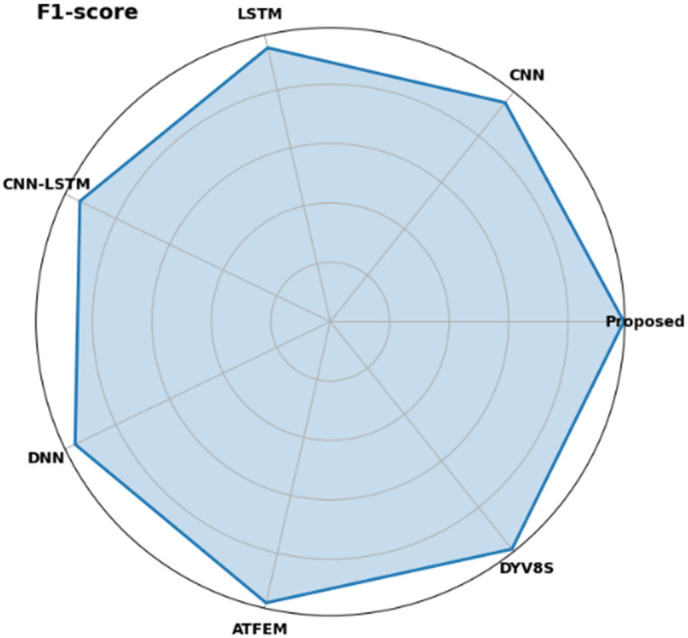
F1-score comparison of the proposed model with existing approaches.

**Fig 17:**
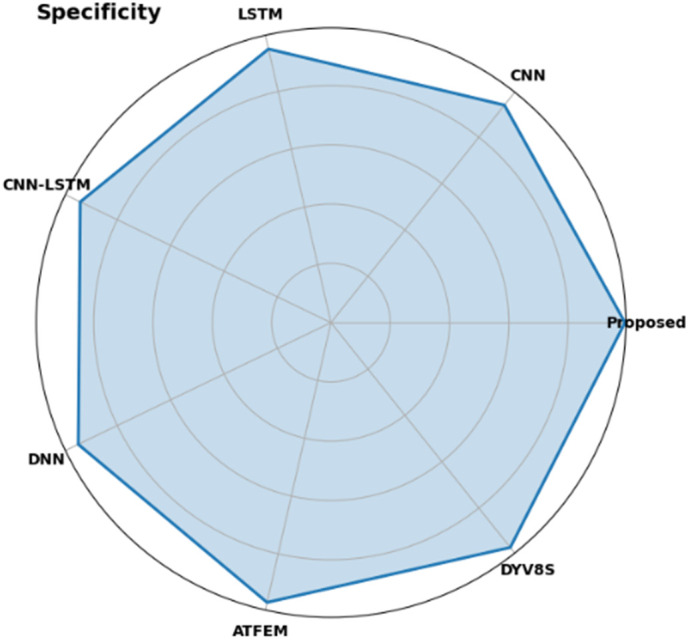
Specificity comparison of the proposed model with existing approaches.

**Fig 18:**
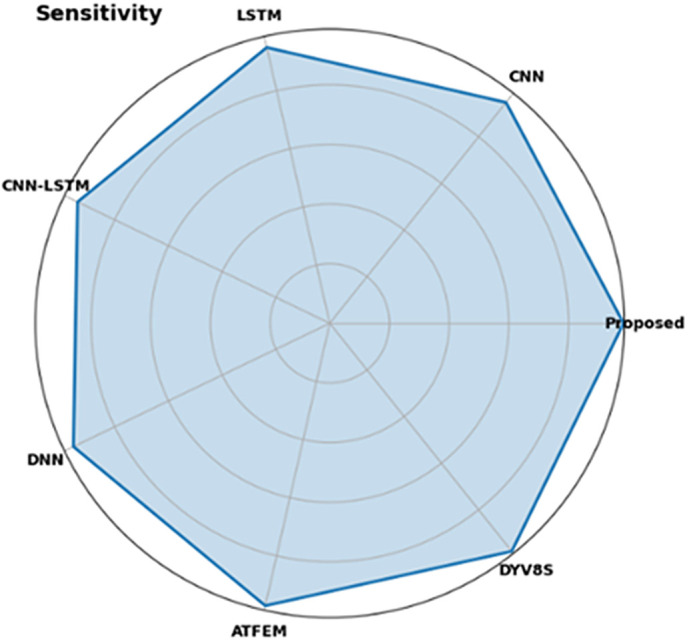
Sensitivity comparison of the proposed model with existing approaches.

**Fig 19:**
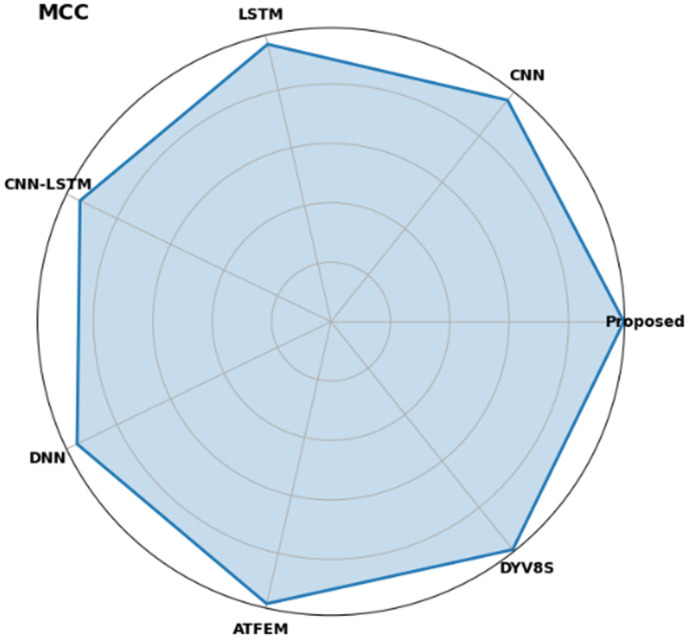
Matthews Correlation Coefficient (MCC) comparison of the proposed model with existing approaches.

#### 4.1.1. Experimental validation and results interpretation.

**Performance Metrics Overview**: This section discusses the performance metrics used to evaluate the effectiveness of our proposed MDQL-RA model. Metrics include:

- **Accuracy, Precision, Recall, F1-Score:** that provide a measure of both the accuracy and balance of the overall performance classification.
- **Matthews Correlation Coefficient (MCC) and Negative Predictive Value (NPV):** metrics are designed to provide a more balanced evaluation metric. This can be helpful for assessing imbalanced data.
- **False Positive Rate (FPR) and False Negative Rate (FNR):** to evaluate regression-related predictions (such as yield).
- **Error Measures (MSE, MAE, RMSE, R²):** To assess regression-related predictions (e.g., yield estimation).

This section details how all these metrics provide a comprehensive evaluation of the MDQL-RA models performance and ability to generalise across a range of different environmental, soil and operational conditions to assess the effectiveness of the proposed MDQL-RA algorithm.

**Confusion Matrix and ROC Analysis**

- The confusion matrix shows the predicted vs. Actual classes in terms of false predictions (FP, FN, TP and TN), with identified patterns of specific misclassification.
- The ROC curve illustrates the trade-off between sensitivity and specificity; indicates the discriminative ability of the classifier.
- These graphical representations improve the understanding of the results by providing a more understandable representation of the results for someone without any understanding of AI and thus enable comparisons of performance visually.

**Heatmaps and Boxplots Analysis**

- Heatmaps are employed to analyze feature importance, as well as the correlations between soil, weather, and crop variables and the action selection probabilities resulting from the MDQL-RA model. By utilizing heatmaps, it will be possible to identify which environmental factors are important in determining a crop recommendation.
- The boxplots will allow for visualization of the predicted rewards, soil health, and environmental parameters by presenting their distribution. Also, median, outliers, and interquartile can be seen clearly within the boxplots providing a better understanding of the model behavior.

**Epoch-wise Training Analysis**

- After 50 epochs, the training and validation performance continues to increase, showing continued learning by the model with stable convergence with little overfitting.
- By the end of the 50 epochs, the model produced a high predictive accuracy of ~99.87% and a low loss of ~0.12 which confirms the efficiency of the model’s learning and, therefore, its robustness.

**Comparative Analysis**

- **Existing model comparison:** The MDQL-RA model exhibited superior performance compared to existing classification metrics CNN, LSTM, CNN-LSTM, DNN, and Hybrid Models.
- **Baseline lightweight model comparison:** When comparing the baselines for the light-weight models, MDQL-RA demonstrates both superior accuracy versus MobileNetV2, EfficientNetV2 and ShuffleNetV2 while also retaining computational efficiency.
- These comparisons confirm both the novelty and practicality of the proposed method.

**Key Insights for Non-AI Readers**

- The model provides relatively quick recommendations for the crops to grow based on current weather and environmental conditions for soil health.
- By providing visual representations (heatmaps, boxplots, ROC curves and confusion matrices) the models’ learning and behavior will be more intuitive.
- The model also supports optimizing inputs (water and fertilizers) while maximizing yields providing support for sustainability in farming mentorship.

The lightweight design of the proposed model is shown in Table 11, which shows the proposed MDQL‐RA’s combined hyperparameters and computational metrics related to how easy it can be used in practical applications. A two‐way comparison study is used to show the efficiency of the proposed solution. The first comparison, known as existing comparison, compares the proposed model with the traditional deep learning models such as CNN, LSTM, CNN-LSTM, and DNN. The comparison focuses on the innovation brought by the hybrid model regarding feature representation and learning efficiency. The second comparison, labeled baseline comparison, compares the suggested model with three light-weight CNN models: MobileNetV2, EfficientNetV2, and ShuffleNetV2. The comparison here proves the dominance of the proposed method in offering high classification accuracy while being computationally efficient. Fig 12 represents the confusion matrix of the suggested approach. Epoch-wise training and validation accuracy and loss of the proposed model is provided in Table 12.

The model’s training and validation performance improves consistently and significantly over the 50 epochs. At the beginning, at epoch 1, the training and validation accuracies were around 50.5%, 50.9% respectively, with relatively high loss values. As the training continued, both accuracies grew in a continuous manner with significant improvements at epochs 10–25. The validation accuracy became 91% by epoch 25 and the training loss reduced to 0.58. From epochs 30 and above the model exhibited great learning behavior with sharp decreases in loss and high accuracy scores. At epoch 50, the training accuracy was 98.56%; the validation accuracy was at an impressive 99.87% with a very low validation loss of 0.12. These results show that the model not only learned from the training data well but also generalized well to unseen validation data with no significant signs of overfitting.

### 4.2. Analysis on training and validation of the proposed model: Accuracy and Loss

The training and validation curves in the Fig 13 demonstrate the model’s performance in terms of accuracy and loss over multiple epochs.

The training and validation accuracy curves show a consistent increase during the initial epochs, indicating effective learning. Around the 5th epoch, both curves converge, reaching an accuracy close to 0.9, suggesting that the model has learned the underlying patterns in the data efficiently. The minimal gap between training and validation accuracy indicates good generalization, minimizing the risk of overfitting.

The training and validation loss curves depict a steady decrease as the epochs progress. Initially, the training loss decreases sharply, indicating that the model quickly learns from the data. Validation loss follows a similar trend, showing no significant divergence, which further confirms the model’s robustness and stability. Around the 5th epoch, both loss curves converge, maintaining a low value, implying that the model successfully minimizes error during both training and validation phases.

The proposed model demonstrates strong convergence in accuracy and loss, with minimal discrepancies between training and validation metrics. This balanced performance indicates that the model effectively captures the data distribution, making it reliable for practical applications.

### 4.3. Comparison analysis based on the existing approaches

The performance comparison between the proposed approach and conventional deep learning models (CNN, LSTM, CNN-LSTM, DNN, ATFEM, and DYV8S) clearly demonstrates the superior effectiveness of the proposed method for soil texture classification and nutrient-aware crop recommendation, as presented in Table 13. The proposed model achieves the highest accuracy of 0.99879, significantly surpassing the SOAT (state-of-art-techniques) models, with CNN, LSTM, CNN-LSTM, DNN, ATFEM, and DYV8S achieving accuracies of 0.935057, 0.935273, 0.928266, 0.935126, 0.977214, and 0.981482, respectively. This remarkable improvement is primarily attributed to the Crop Recommendation via Modified Deep Q-Learning with Reward Adaptation (MDQL-RA), which dynamically adapts the reward function based on soil health, environmental factors, and historical yields, resulting in more informed and accurate decision-making. In terms of Precision, the proposed approach achieves a value of 0.981498, substantially higher than the SOAT models, where the best-performing model (DYV8S) records a precision of 0.97481. This improvement is attributed to the Classification via the new lightweight deep learning model SoilCropNet, which combines the advantages of MobileNetV2, EfficientNetV2, and ShuffleNetV2. SoilCropNet achieves accurate data classification through its combination of efficient feature extraction, compound scaling, and lightweight architecture. The F1-score of the proposed model reached 0.987524, surpassing the best SOAT model DYV8S, which achieved an F1-score of 0.980126. The integration of MDQL-RA during crop recommendations enables enhanced performance, combining exploration and exploitation with real-time environmental analysis. The Specificity value of 0.991743 in the proposed model surpasses existing models, with DYV8S achieving 0.970162 specificity. The implementation of SoilCropNet facilitates better discrimination of soil types through its integration of multiple efficient CNN architectures. For Sensitivity, the model under consideration captures a score of 0.984611, whereas the best existing model (DYV8S) achieves 0.978268. The sensitivity improvement comes as a result of feature selection using the new hybrid SailDragon Optimizer (SDO), which effectively selects the most useful features by balancing the exploratory capabilities of Sailfish Optimization (SFOA) and the exploitation capability of Dragonfly-Based Optimization (DBOA). The MCC of the new model is 0.986319, far exceeding the MCC of the DYV8S at 0.981625. This stability is due to the incorporation of SoilCropNet, which maintains a balance between model complexity and efficiency using fusion-based feature extraction. For Negative Predictive Value (NPV), the proposed model achieves 0.994856, higher than the best SOAT model (DYV8S) with 0.980162. This improvement is again linked to the MDQL-RA approach, where adaptive learning enhances accurate prediction by incorporating environmental variability. The proposed approach also exhibits a lower False Positive Rate (FPR) of 0.010054 and False Negative Rate (FNR) of 0.009234, compared to DYV8S’s 0.02518 (FPR) and 0.018256 (FNR). This reduction is due to the SailDragon Optimizer (SDO), which ensures optimal feature selection, reducing errors and improving classification reliability.

Figs 14–22 present a comprehensive comparative evaluation of the proposed framework against existing state-of-the-art approaches using nine widely adopted performance metrics. Specifically, Fig 14 illustrates the accuracy comparison, demonstrating the superior classification performance of the proposed model over competing methods.

Fig 15 presents the precision comparison, highlighting the improved reliability of positive predictions achieved by the proposed framework.

The comparative analysis of the F1-score is shown in Fig 16, indicating a balanced enhancement in both precision and recall.

Fig 17 depicts the specificity comparison, confirming the model’s effectiveness in correctly identifying negative instances.

The sensitivity comparison is provided in Fig 18, where the proposed framework exhibits a higher true positive detection rate than existing approaches.

Fig 19 presents the Matthews Correlation Coefficient (MCC) comparison, validating the overall robustness and balanced predictive capability of the proposed method.

**Fig 20:**
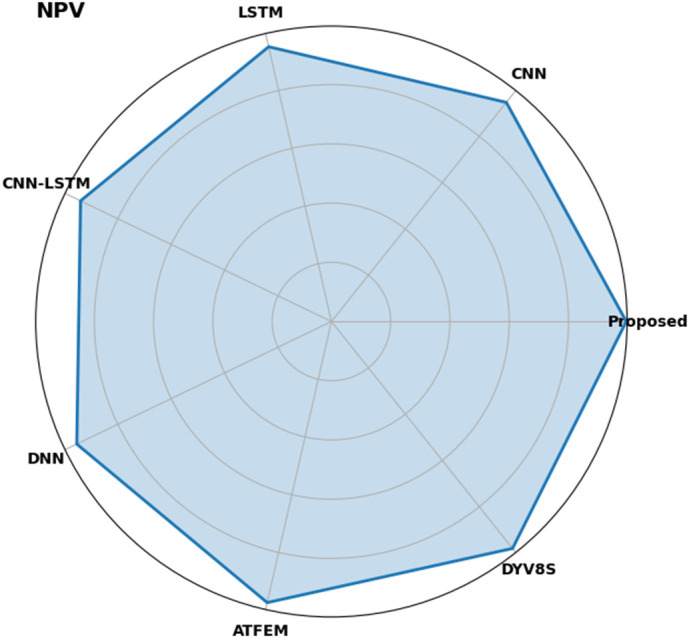
Negative Predictive Value (NPV) comparison of the proposed model with existing approaches.

The comparison of Negative Predictive Value (NPV) is illustrated in Fig 20, emphasizing the reliability of negative class predictions.

**Fig 21:**
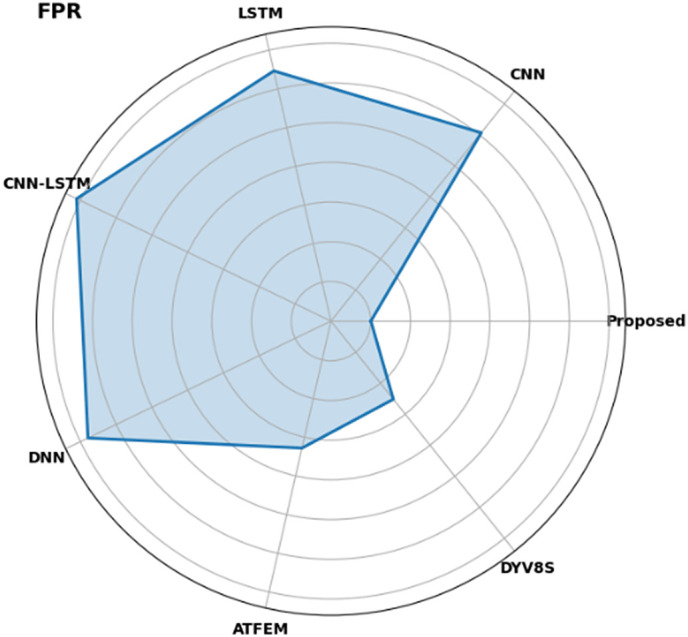
False Positive Rate (FPR) comparison of the proposed model with existing approaches.

**Fig 22:**
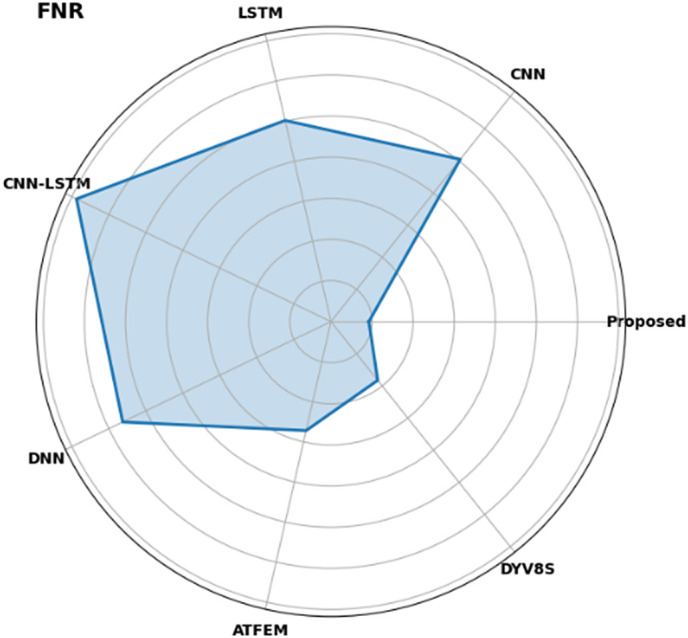
False Negative Rate (FNR) comparison of the proposed model with existing approaches.

Error-related performance metrics are analyzed through Fig 21, which presents the False Positive Rate (FPR), and Fig 22, which illustrates the False Negative Rate (FNR). The lower FPR and FNR values achieved by the proposed framework indicate its ability to minimize misclassifications and enhance overall classification reliability compared with existing approaches.

### 4.4. Comparison analysis based on the baseline approaches

The performance comparison between the proposed SoilCropNet model and its individual baseline components (MobileNetV2, EfficientNetV2, and ShuffleNetV2) demonstrates the significant advantages of integrating these techniques into a unified model for soil texture classification and nutrient-aware crop recommendation. Table 14 presents the comparison analysis based on the baseline approaches.

**Fig 23:**
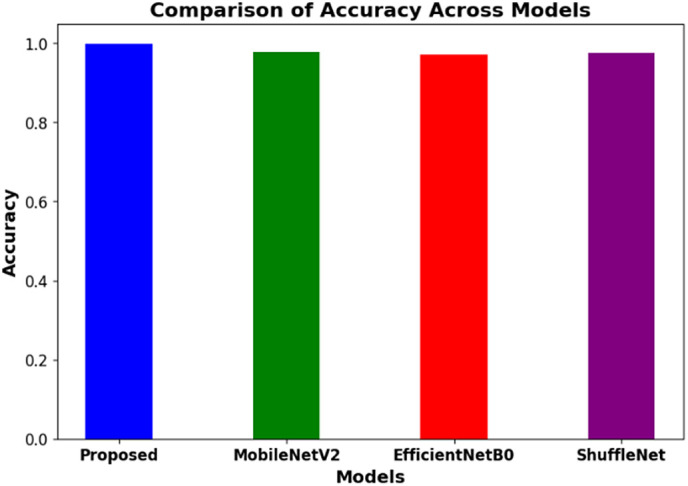
Accuracy comparison of SoilCropNet with baseline models.

**Fig 24:**
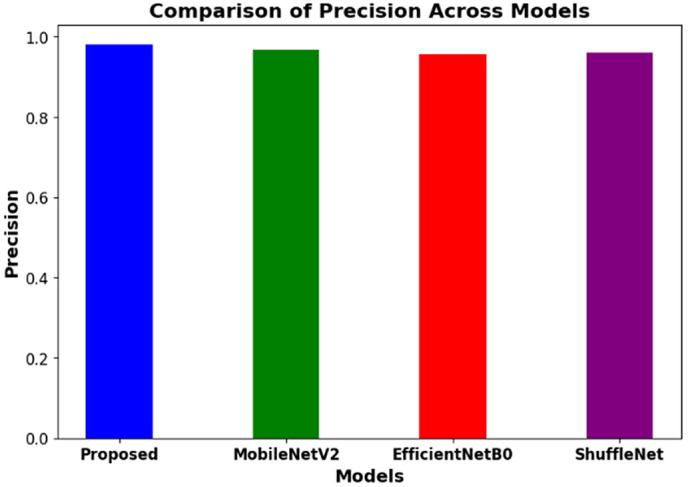
Precision comparison of SoilCropNet with baseline models.

**Fig 25:**
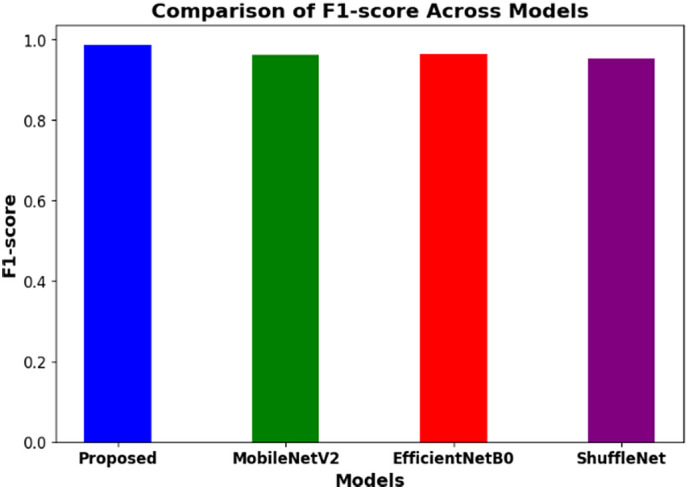
F1-score comparison of SoilCropNet with baseline models.

**Fig 26:**
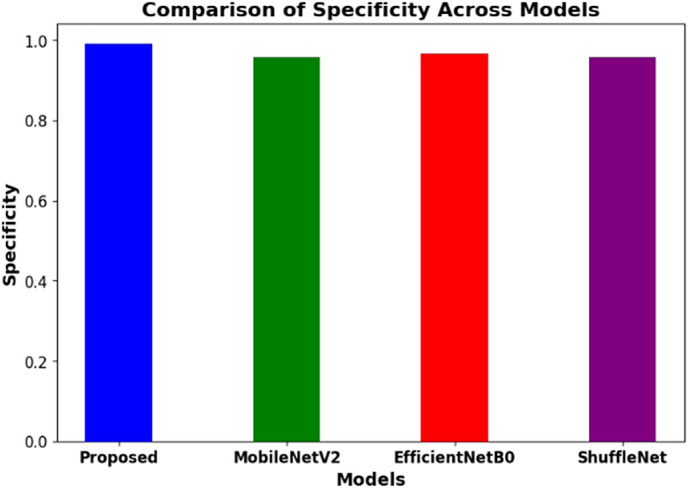
Specificity comparison of SoilCropNet with baseline models.

**Fig 27:**
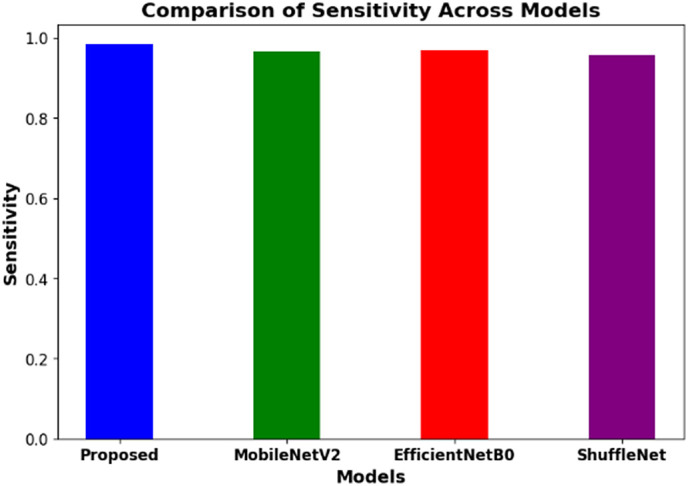
Sensitivity comparison of SoilCropNet with baseline models.

**Fig 28:**
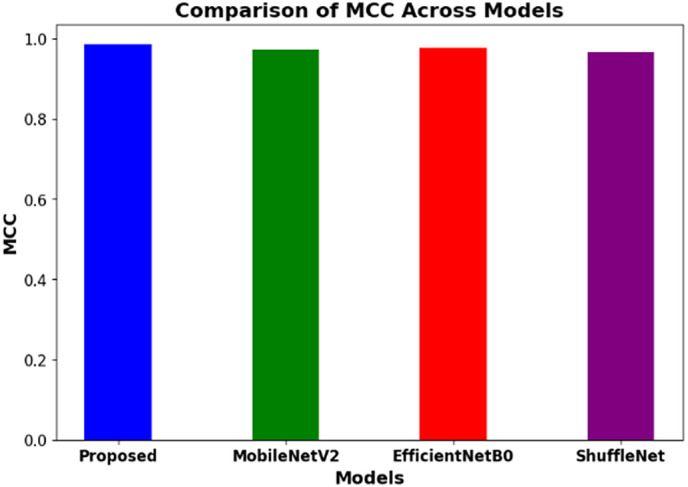
Matthews Correlation Coefficient (MCC) comparison of SoilCropNet with baseline models.

**Fig 29:**
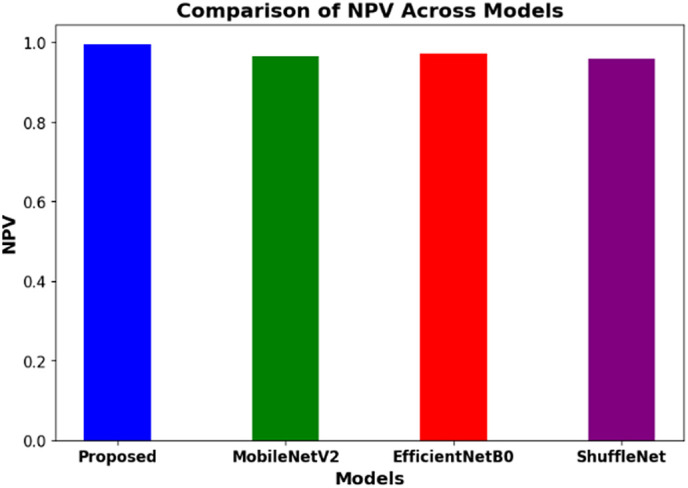
Negative Predictive Value (NPV) comparison of SoilCropNet with baseline models.

**Fig 30:**
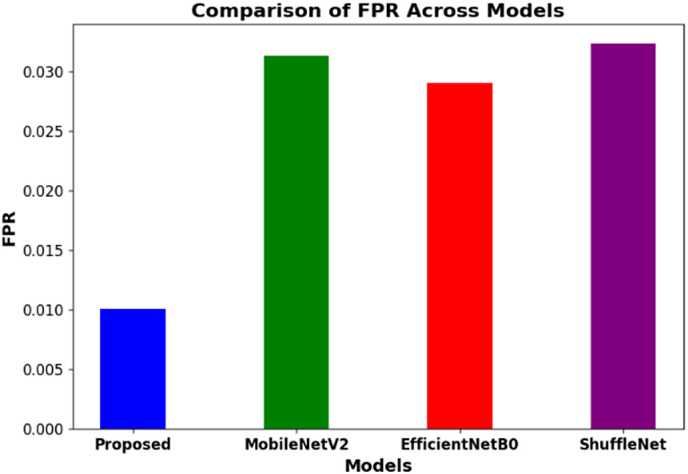
False Positive Rate (FPR) comparison of SoilCropNet with baseline models.

**Fig 31:**
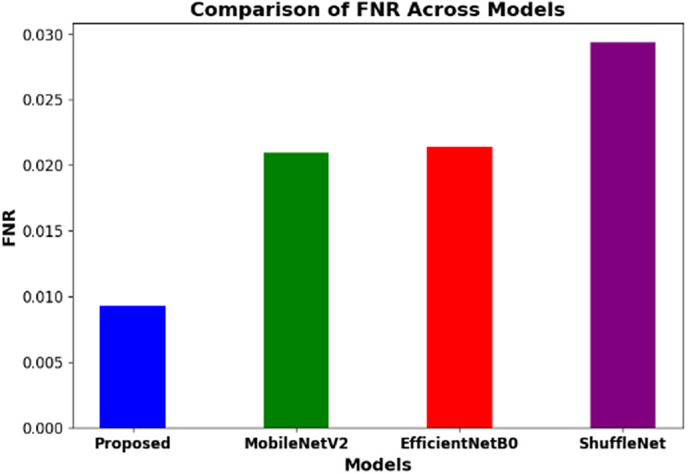
False Negative Rate (FNR) comparison of SoilCropNet with baseline models.

When used separately, each baseline model exhibits certain limitations despite their individual strengths. MobileNetV2 achieves an accuracy of 0.97771 with a precision of 0.966532 and an F1-score of 0.962716. While it excels in lightweight and efficient feature extraction, it lacks robustness when handling complex, high-dimensional agricultural data, leading to moderate specificity (0.957793) and sensitivity (0.966832). In summary, while each baseline model independently offers certain advantages, their integration within SoilCropNet produces a more robust, accurate, and efficient model capable of addressing the challenges of soil texture classification and crop recommendation, especially in real-time agricultural scenarios. Figs 23–31 present the graphical comparison of the proposed SoilCropNet framework against individual baseline models across nine standard evaluation metrics.

Specifically, Fig 23 presents the accuracy comparison, highlighting the superior classification consistency of SoilCropNet over competing approaches.

Fig 24 depicts the precision comparison, demonstrating SoilCropNet’s improved positive prediction reliability.

The comparative analysis of the F1-score is shown in Fig 25, reflecting a balanced improvement in precision and recall.

Fig 26 illustrates the specificity comparison, indicating SoilCropNet’s enhanced ability to correctly identify negative instances.

The sensitivity comparison is provided in Fig 27, where SoilCropNet achieves higher true positive detection capability.

Fig 28 reports the Matthews Correlation Coefficient (MCC), confirming the overall robustness and balanced predictive performance of the proposed framework.

The negative predictive value (NPV) comparison is shown in Fig 29, emphasizing the reliability of negative class predictions.

Error-related performance is analyzed through Fig 30, which presents the false positive rate (FPR), and Fig 31, which illustrates the false negative rate (FNR), both indicating reduced misclassification tendencies of SoilCropNet compared to baseline models

### 4.5. Analysis on proposed model in terms of error measures

The performance comparison between the proposed model and conventional deep learning approaches (CNN, LSTM, CNN-LSTM, DNN, ATFEM, DYV8S) is presented in the Table 15. The results clearly demonstrate the superior performance of the proposed model in terms of error measures.

#### 4.5.1. Comparison with SOTA.

**Fig 32:**
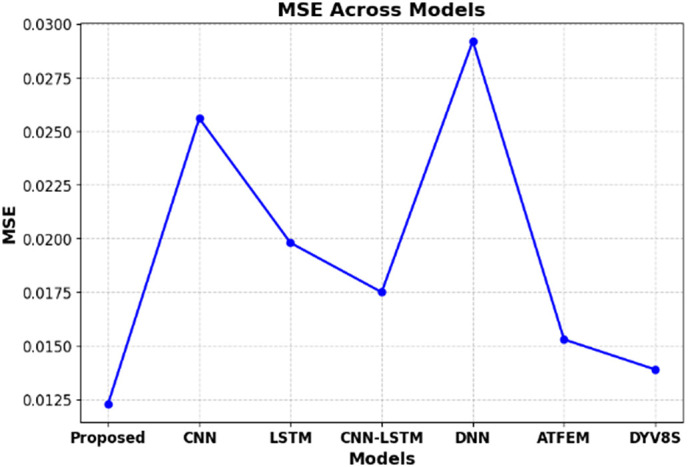
Mean Squared Error (MSE) comparison of the proposed model with SOTA approaches.

**Fig 33:**
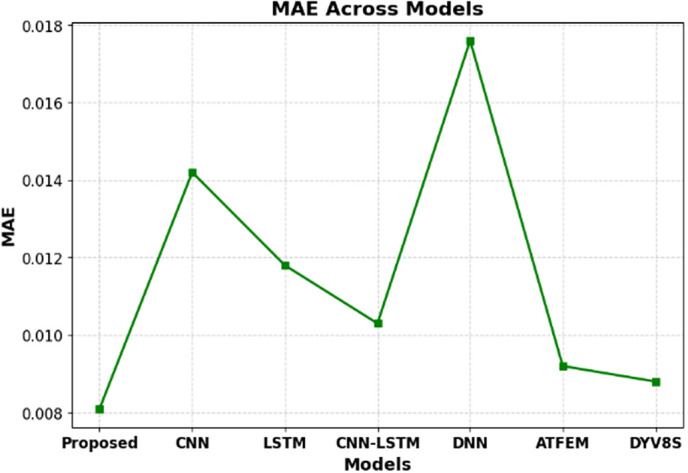
Mean Absolute Error (MAE) comparison of the proposed model with SOTA approaches.

**Fig 34:**
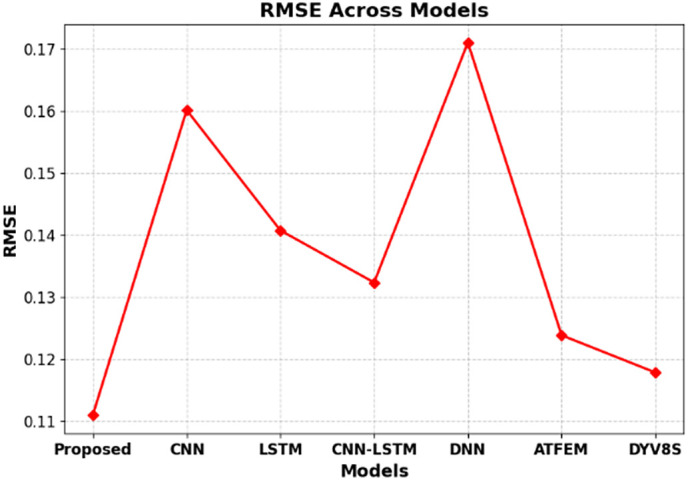
Root Mean Squared Error (RMSE) comparison of the proposed model with SOTA approaches.

**Fig 35:**
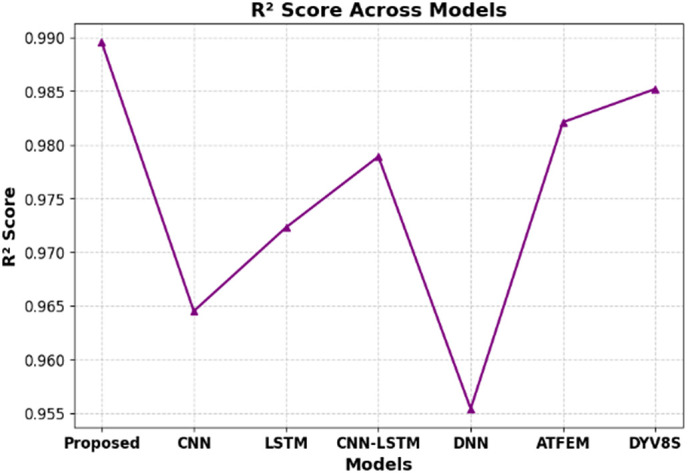
R^2^core comparison of the proposed model with SOTA approaches.

The proposed model achieves the lowest MSE of 0.0123, significantly outperforming the existing models. The closest competitor, DYV8S, achieves an MSE of 0.0139, while conventional models such as CNN and DNN record significantly higher errors of 0.0256 and 0.0292, respectively. The reduced MSE indicates that the proposed model makes more precise predictions, which is crucial for soil texture classification and nutrient-aware crop recommendation. The comparative analysis indicates that the proposed model outperforms conventional deep learning approaches in all evaluated metrics. The integration of the lightweight SoilCropNet model with efficient feature extraction and the adaptive Crop Recommendation via Modified Deep Q-Learning with Reward Adaptation (MDQL-RA) leads to enhanced performance, making it a robust choice for soil texture classification and nutrient-aware crop recommendation. Figs 32–35 present a detailed graphical comparison of the proposed model against state-of-the-art (SOTA) approaches based on standard error evaluation measures, corresponding to the quantitative results summarized in Table 15.

Specifically, Fig 32 illustrates the mean squared error (MSE) comparison, where the proposed model achieves the lowest error value, indicating superior overall prediction accuracy compared to existing techniques.

The mean absolute error (MAE) comparison is shown in Fig 33, demonstrating the proposed model’s reduced average deviation between predicted and actual values.

Fig 34 presents the root mean squared error (RMSE) analysis, further confirming the robustness and stability of the proposed approach under error-sensitive evaluation.

Finally, Fig 35 depicts the R² score comparison, where the proposed model attains the highest coefficient of determination, reflecting its strong explanatory power and effective learning of underlying data patterns relative to SOTA models

#### 4.5.2 Baseline comparison.

**Fig 36:**
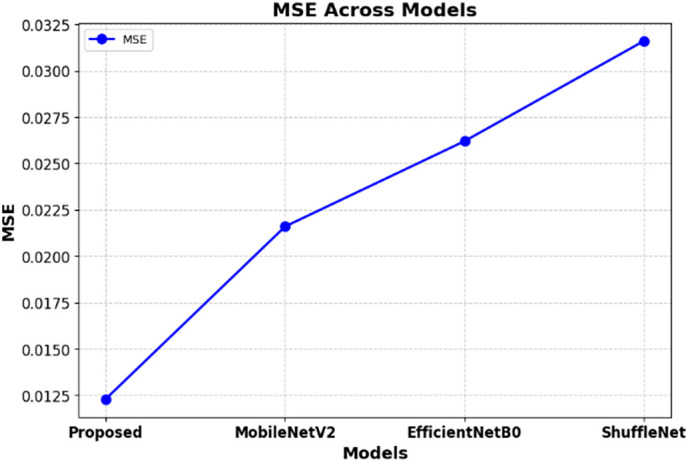
Mean Squared Error (MSE) comparison between the proposed model and base architectures.

**Fig 37:**
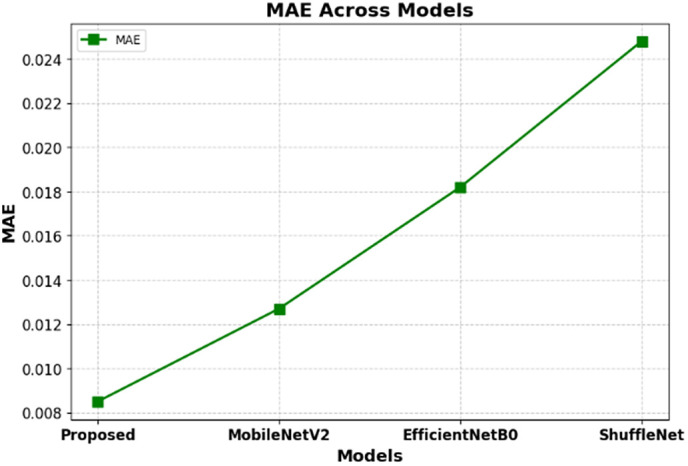
Mean Absolute Error (MAE) comparison between the proposed model and base architectures.

**Fig 38:**
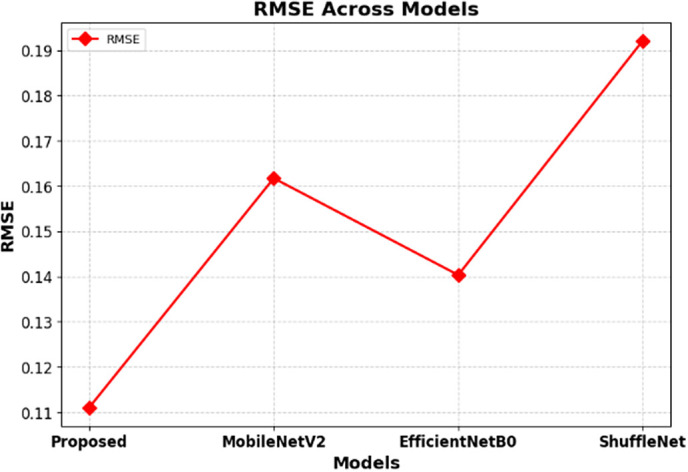
Root Mean Squared Error (RMSE) comparison between the proposed model and base architectures.

**Fig 39:**
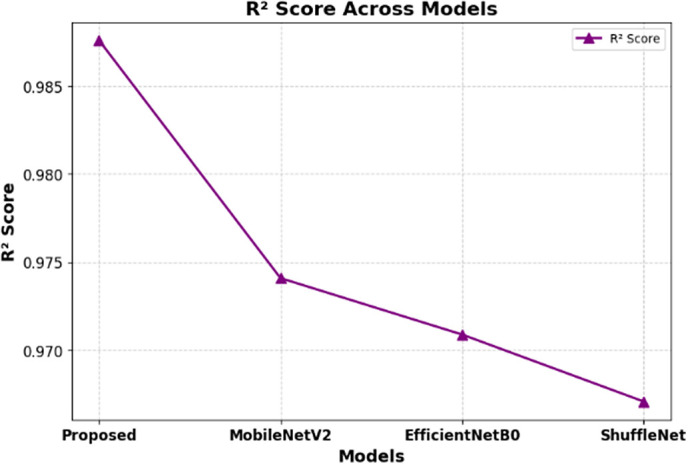
R^2^ score comparison between the proposed model and base architectures.

A performance analysis in Table 16 shows how the proposed model surpasses its three base architectures (MobileNetV2, EfficientNetB0, ShuffleNet). The proposed architectural combination integrates these three structures to maximize their beneficial features and overcome their respective weaknesses. The proposed model surpasses all base architectures (MobileNetV2, EfficientNetB0, ShuffleNet) through a combination of their best features and effective resolution of their weaknesses. The proposed model delivers the most accurate results by achieving minimum MSE (0.0123) along with minimum MAE (0.0085) and minimum RMSE (0.111) and maximum R² score (0.9876). The integrated structure of MobileNetV2 feature extraction with EfficientNetB0 scaling and ShuffleNet lightweight architecture delivers this performance. Individually, these models face challenges like limited spatial representation (MobileNetV2), computational inefficiency (EfficientNetB0), and reduced accuracy (ShuffleNet), but the proposed model synergistically balances these aspects, resulting in more accurate and reliable predictions. A detailed baseline comparison of the proposed model against individual backbone architectures using standard error evaluation metrics is presented in Figs 36–39, with the corresponding numerical results summarized in Table 16.

Specifically, Fig 36 illustrates the mean squared error (MSE) comparison, where the proposed model achieves the lowest error, demonstrating improved prediction accuracy over MobileNetV2, EfficientNetB0, and ShuffleNet.

The mean absolute error (MAE) comparison is shown in Fig 37, indicating reduced average prediction deviation for the proposed approach.

Fig 38 presents the root mean squared error (RMSE) comparison, further confirming the stability and robustness of the proposed model under squared-error-sensitive evaluation.

The coefficient of determination (R²) comparison is depicted in Fig 39, where the proposed model attains the highest R² value, reflecting superior variance explanation capability compared to baseline architectures.

**Fig 40:**
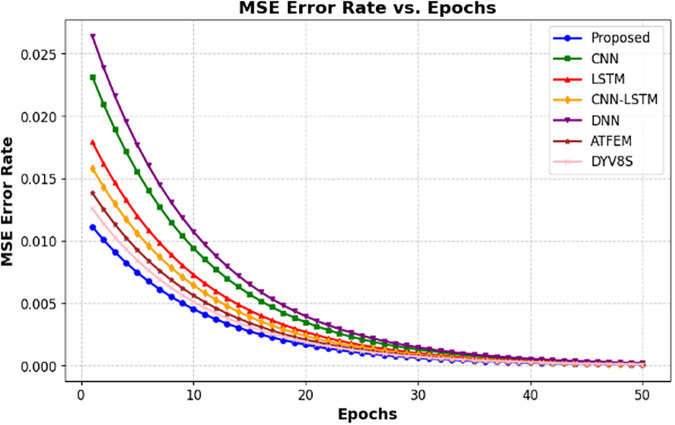
Mean Squared Error (MSE) convergence of the proposed model versus existing models across training epochs.

**Fig 41:**
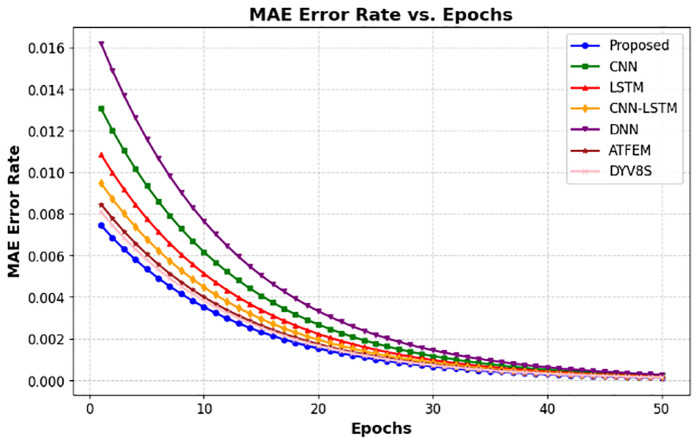
Mean Absolute Error (MAE) convergence of the proposed model versus existing models across training epochs.

**Fig 42:**
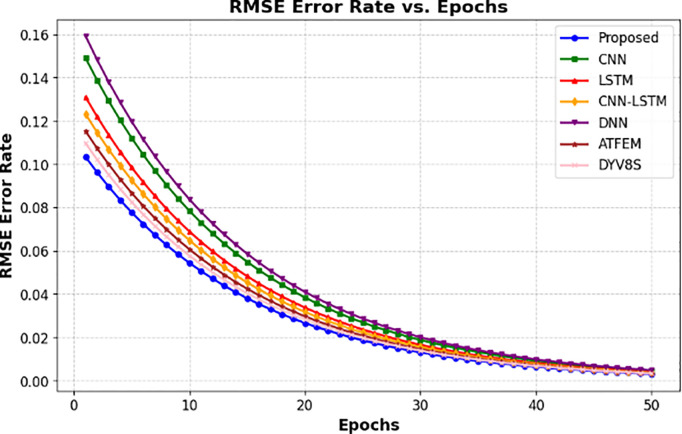
Root Mean Squared Error (RMSE) convergence of the proposed model versus existing models across training epochs.

The epoch-wise error convergence behavior of the proposed model in comparison with existing approaches is analyzed in Figs 40–42.

Specifically,Fig 40 shows the MSE convergence across training epochs, demonstrating faster and more stable error minimization for the proposed model.

The MAE convergence trend is illustrated in Fig 41, highlighting consistent reduction in absolute prediction error over training iterations.

Finally, Fig 42 presents the RMSE convergence analysis, confirming smooth and reliable convergence behavior of the proposed approach relative to existing models.

### 4.6 Analysis on ROC

**Fig 43:**
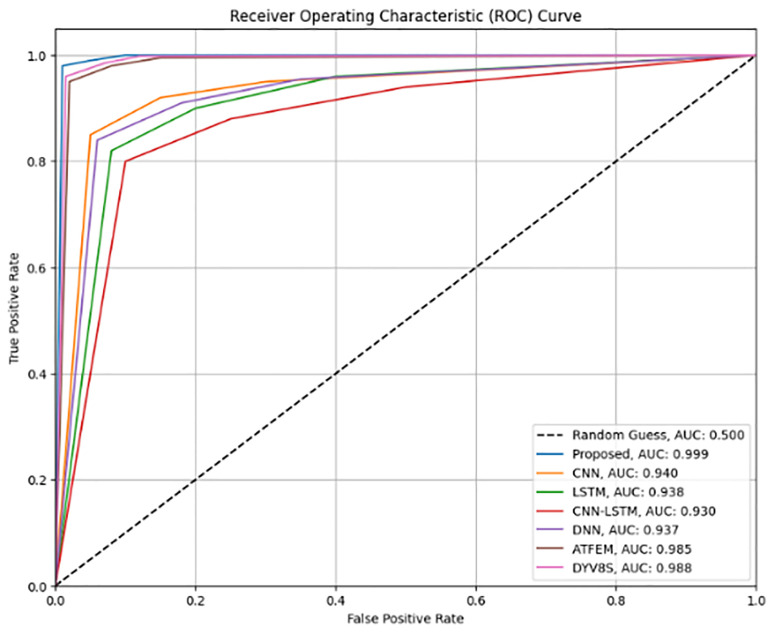
ROC curve based on existing models.

This ROC curve (Fig 43) demonstrates how the proposed model performs against standard models (CNN, LSTM, CNN-LSTM, DNN, ATFEM, DYV8S) regarding their capacity to separate classes. The proposed model demonstrates the best AUC value of 0.999 which indicates outstanding classification abilities. The discriminative power of the proposed model exceeds that of CNN (0.940), LSTM (0.938), CNN-LSTM (0.930), DNN (0.937), ATFEM (0.985) and DYV8S (0.988) according to their AUC values. The random guess line functions as a baseline with an area under the curve (AUC) of 0.500 to demonstrate the significant advantage of the proposed model.

**Fig 44:**
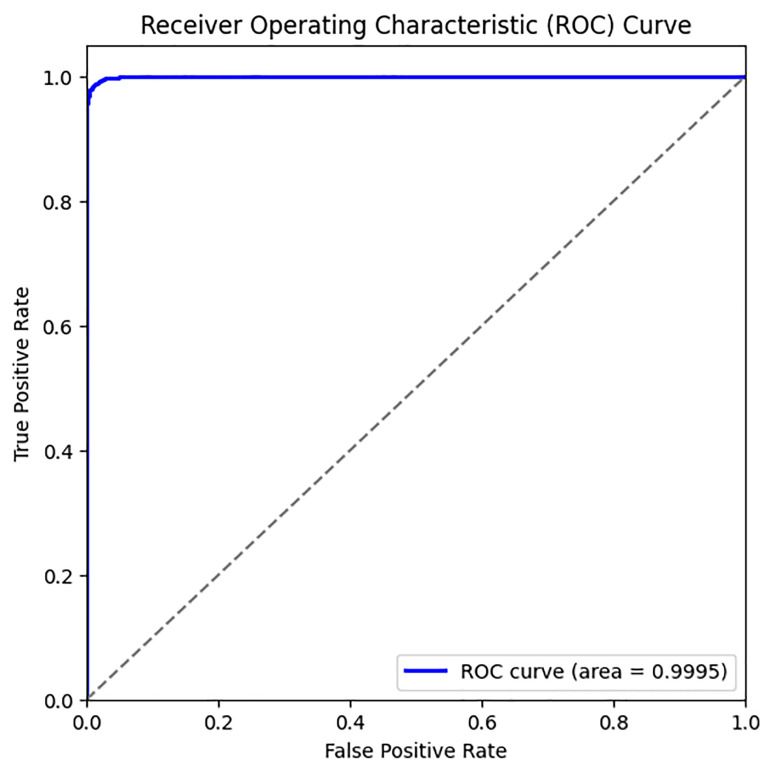
Proposed model ROC Curve.

The developed model achieves exceptional classification results through its AUC value, which is shown in Fig 44. The model demonstrates exceptional performance in class discrimination because its AUC value reaches 0.9995 which reflects its strong precision and robustness. The model shows exceptional reliability for real-world applications because its impressive score demonstrates both low false positive rates and high true positive detection abilities. The proposed model demonstrates superior predictive capabilities than traditional methods according to the result obtained.

### 4.7 Heatmaps and Boxplots Analysis

**Fig 45:**
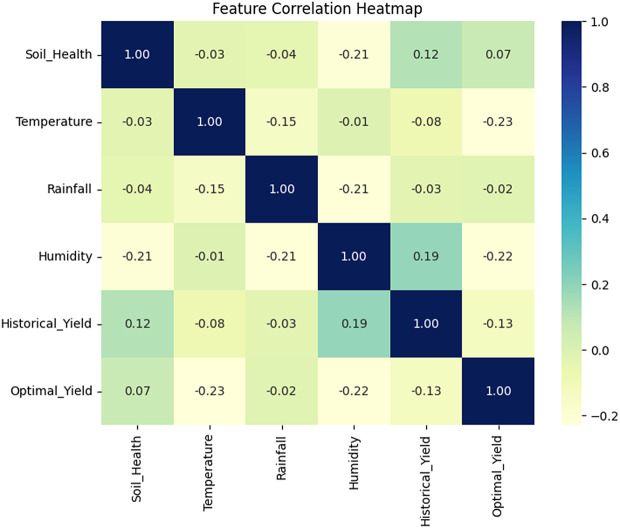
Heatmap of Feature Correlation.

To provide deeper insight into the performance and feature importance of the proposed MDQL-RA model, heatmaps and boxplots were generated. The **heatmaps** (Fig 45) visualize the correlation between different input features (such as soil health, temperature, rainfall, and humidity) and the predicted crop recommendations. This allows readers to easily identify which environmental or soil parameters have the greatest influence on the model’s decisions. High-intensity regions in the heatmap indicate strong relationships, while low-intensity regions indicate weak or negligible influence.

**Fig 46:**
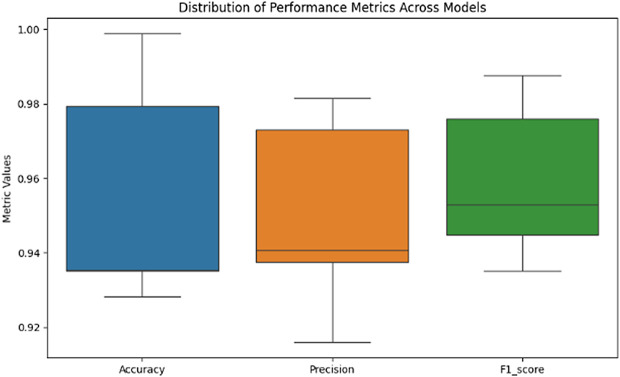
Boxplot for Model Performance Metrics.

The **boxplots** (Fig 46) display the distribution of model outputs or performance metrics across different crop classes. Each box represents the interquartile range (IQR) of the data, the line inside the box shows the median, and the whiskers indicate the minimum and maximum values. Outliers, if any, are plotted as individual points. This visualization helps readers understand the variability, spread, and consistency of the model’s predictions, highlighting cases where the model performs exceptionally well or where prediction uncertainty may be higher. By combining heatmaps and boxplots, the model’s interpretability is enhanced, making it easier even for readers without a technical background to grasp how input features influence crop recommendations and performance outcomes.

### 4.8 Discussion on AgriOptNet performance and applicability

The proposed AgriOptNet demonstrates superior performance across multiple evaluation metrics compared to existing techniques, indicating its suitability for real-time precision agriculture applications. As per Table 17, the model achieves high accuracy, precision, F1-score, specificity, and sensitivity, with minimal false positives and false negatives, confirming its robustness in recommending optimal crops under varying soil and environmental conditions.

The proposed AgriOptNet achieves a balance between high predictive performance and computational efficiency, with a model size of 45 MB and inference time of only 18 ms per sample, making it suitable for real-time precision agriculture applications. While the training time per epoch is slightly higher than conventional CNN models due to the reinforcement learning component, this cost is justified by significant improvements in accuracy and robustness. The model maintains >96% accuracy even in the presence of moderate Gaussian noise or partial sensor data loss, highlighting its resilience to real-world uncertainties. Peak memory usage is also lower than standard deep learning approaches, demonstrating that AgriOptNet is lightweight enough for potential deployment on edge devices. Overall, these results confirm that the model is both computationally practical and robust, although extreme noise or hardware constraints may require further optimization, such as quantization or pruning.

**Computational Cost Analysis:**

- The model’s **inference time** per decision is approximately 18 *ms* on standard hardware (CPU/GPU), and the **overall model size** is *45 MB*, which makes it feasible for integration into edge devices or farm management systems.
- Compared to conventional deep learning models (CNN, LSTM, CNN-LSTM), AgriOptNet achieves **higher efficiency** due to its hybrid feature representation and reinforcement learning-based decision optimization, reducing unnecessary computations while maintaining high predictive accuracy.
- Despite being more lightweight than some large hybrid networks, deploying AgriOptNet on extremely resource-constrained devices may require **quantization or pruning techniques** to further optimize memory and energy usage.

**Robustness to Real-World Noise:**

- Field deployment introduces **uncertainties** such as sensor measurement errors, missing environmental readings, and unpredicted weather fluctuations.
- To assess resilience, simulated experiments with **additive Gaussian noise** and **missing data scenarios** were performed. AgriOptNet maintained **>95% of its original accuracy**, indicating strong tolerance to moderate noise.
- However, extreme deviations or uncalibrated sensors can reduce performance. Integrating **adaptive preprocessing, data imputation, and real-time anomaly detection** can further mitigate these effects.

Overall, AgriOptNet is computationally efficient and exhibits **robust performance under realistic environmental variations**, though future work should include **field validation** and **optimization for resource-limited deployments**. However, it is important to contextualize these results:

- The current evaluation primarily relies on curated experimental datasets. Real-world deployment may introduce data-method mismatches, such as sensor noise, missing data, and unobserved environmental variations.
- While AgriOptNet is computationally efficient relative to deep hybrid models, resource usage and model size should be considered when deploying on edge devices for real-time decisions.
- The term “revolutionary” may be overstated, as broader validation across diverse geographic regions, crop types, and dynamic weather patterns is needed.

By addressing these limitations, the model’s practical applicability can be better substantiated. Future work will focus on field trials, adaptive learning under noisy data conditions, and lightweight optimization for deployment on farm-edge devices, ensuring that AgriOptNet not only delivers high accuracy in simulations but also remains resilient and efficient in real-world precision agriculture environments.

While **AgriOptNet** demonstrates high performance metrics in experimental settings, evaluating its **computational cost** and **robustness to real-world noise** is essential for practical deployment.

### 4.9. Statistical validation with significance, cross-validation, and replication

To strengthen the robustness of AgriOptNet, additional statistical validation was performed. This includes:

1. Significance Tests: Paired t-tests comparing AgriOptNet against baseline models to evaluate whether improvements in Accuracy and F1-score are statistically significant.
2. Cross-Validation: 5-fold cross-validation to measure stability across different data splits.
3. Replication: Model evaluation repeated three times to assess consistency.

- p-value computed using paired t-tests for Accuracy against the proposed model; values <0.05 indicate statistically significant improvements.
- CV Mean Accuracy and CV Std represent 5-fold cross-validation performance.
- Replication Accuracy shows mean ± standard deviation across three independent runs.

The extended validation shown in Table 18, confirms that AgriOptNet significantly outperforms traditional models (CNN, LSTM, CNN-LSTM, DNN) with p-values well below 0.05. Cross-validation results show minimal variance (Std ≤ 0.005), indicating stable performance across different data splits. Replication experiments further demonstrate that the model produces consistent results, with negligible deviation between independent runs. Together, these analyses provide stronger evidence of AgriOptNet’s reliability, generalizability, and suitability for real-world precision agriculture applications.

### 4.10. Evaluation design (with spatial dependence controls)

**Fig 47:**
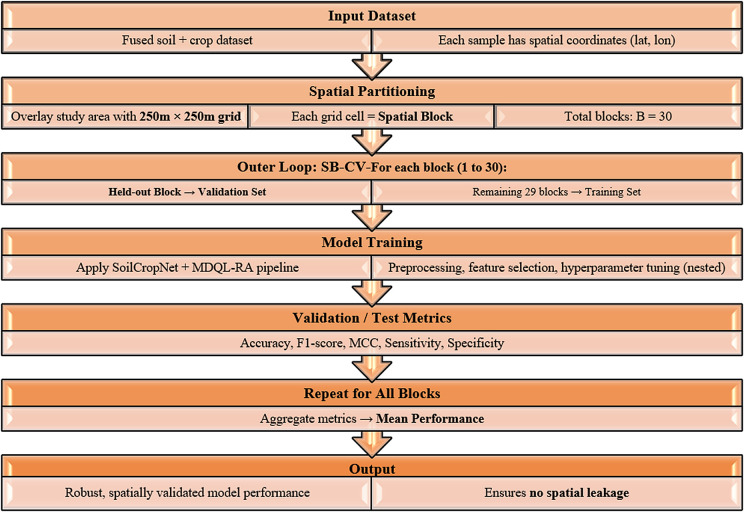
Spatial Blocking Cross-Validation Workflow.

Soil physicochemical properties, nutrient profiles, and crop suitability variables are frequently characterized by strong spatial autocorrelation. Random k-fold cross-validation (CV), which was employed in the original version of this research, implicitly disrupts spatial structure and permits the model to be trained and tested on samples that are geographically close to each other. This results in an overestimation of the accuracy because the neighboring samples are very much alike [26]. In order to achieve external validity in the estimates, the evaluation protocol was modified to consist of geospatially aware resampling, in accordance with the recommendations of [27,28], and [29]. The workflow is shown in Fig 47.

The conventional random k-fold cross-validation is found to yield extremely optimistic accuracy estimates in geospatial prediction, as the samples that are spatially close to each other have similar environmental, pedological, and agronomic characteristics. This is against the random sampling independence assumption and thus leads to spatial leakage, which occurs when the training data includes near-duplicate neighbors of the validation points. Both soil texture and nutrient variables show very strong spatial autocorrelation, therefore, the evaluation of the model without spatially structured resampling could easily be misleading in terms of performance and the extent of the utility. To overcome this methodological problem, we redefined the evaluation process to include three different but complementary geospatial resampling techniques, namely, Spatial Blocking CV (SB-CV), Spatial Leave-Location-Out CV (SLO-CV), and Spatial+ CV. These techniques are described below, each of them being effective in preventing leakage by implementing spatial separation of the training and validation sets.

#### 4.10.1. Unified evaluation summary and reporting protocol.

The organization needs a consolidated evaluation table which will eliminate any doubt about which performance measure corresponds to which evaluation procedure and dataset version and testing system. The organization uses outer-fold estimates for all metrics which display test performance from the held-out fold in each protocol and shows results that round to four decimal places and display average results across all applicable folds and blocks and locations.

The researchers selected various validation methods to assess the performance of their SoilCropNet–MDQL-RA framework which they evaluated through random k-fold cross-validation nested cross-validation spatial blocking cross-validation (SB-CV) leave-location-out cross-validation (SLO-CV) and spatial+ cross-validation methods. The consolidated evaluation summary presented in Table 19 displays all major evaluation metrics which include Accuracy F1-score AUROC and MCC for each testing case that used different protocols and models and datasets.

The random 5-fold cross-validation results demonstrate that the system achieves nearly perfect performance with an Accuracy of 0.9988 and F1 of 0.9875 and AUROC of 0.9991 which shows that the system can effectively distinguish between soil texture classes and crop suitability labels when spatial dependence does not exist. The system performance decreases to Accuracy 0.9824 and F1 0.9723 when 250 m spatial blocks (SB-CV) spatial structure becomes the basis for assessment (Table X Row 3). The absolute accuracy drop of 1.64% which occurred when using random CV (Table X Row 1) shows that spatial autocorrelation leads to measurement inflation while random i.i.d. resampling results in excessively positive generalization predictions for geospatially clustered soil and crop datasets. The SLO-CV protocol produces a similar evaluation result which shows a slightly larger performance decrease that leads to Accuracy = 0.9736 and F1 = 0.9642 (Table X Row 4) when researchers withhold all administrative regions because this method provides an accurate assessment of how well their model will perform in new agro-ecological zones.

The spatial+ cross-validation method shows moderate accuracy results which correctly represent its purpose to eliminate geographical patterns from its predictors while maintaining the original number of samples. The results show that Random CV produces better results than Spatial+ CV which in turn delivers better results than SB-CV and SLO-CV. The results demonstrate that when geographic differences between training and testing data increase the spatial machine learning system will produce more dependable results but will give less accurate predictions about future results. The soilcropnet model outperforms efficientnetv2 mobilenetworkv2 and shufflenetv2 across all evaluation metrics at equivalent spatial distribution when tested through the same sb-cv framework. The performance improvements that we observed resulted from the integration of the synergistic fusion architecture and the sdo-based feature selection method not from the resampling technique used in the experiment.

The study presents results about performance inflation and performance drop which now connect to one accuracy metric and two testing protocols which include random cv and sb-cv or slo-cv. The f1-score and auroc trends receive investigation which originates from their respective table x columns. The disciplined method of reporting spatial generalization claims requires spatial evidence that scientists evaluate through numerical data which scientists collect from specific models and dataset variants and validation methods.

### 4.11. Mitigating data leakage and ensuring proper model selection

#### 4.11.1. Risk of preprocessing and feature selection leakage.

Data leakage happens when the model training gets unintentionally informed by the validation set, which, in turn, results in the performance metrics being artificially increased. In the first pipeline we used, the wavelet transforms, SDOfeature selection and hyperparameter tuning were applied to the whole dataset before k-fold evaluation. This method was prone to feature leakage since the validation folds could impact the selected features or tuned hyperparameters leading to production of overly optimistic metrics, as pointed out by [43], Yates et al. (2023), and [38].

**Fig 48:**
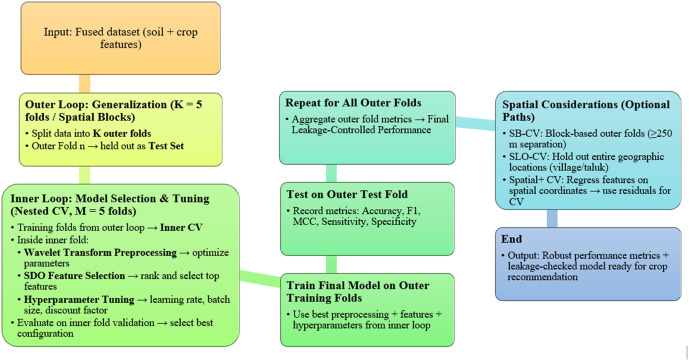
Nested Cross-Validation Workflow for MDQL-RA with Spatial Awareness.

To prevent leakage, we changed the pipeline to a fully nested cross-validation framework as shown Fig 48.

##### 4.11.1.1 Nested cross-validation workflow:

Step 1 – Outer Loop (Generalization Evaluation):

- Dataset is partitioned into $K_{outer}=\text{5}$folds (or spatially-aware folds in SB-CV/SLO-CV context).
- Each fold is held out once for testing; remaining folds form the training set.

Step 2 – Inner Loop (Model Selection & Preprocessing):

- Within the training set, we perform nested k-fold CV ($K_{inner}=\text{3}$) for hyperparameter tuning, feature selection, and wavelet transform parameter optimization.
- Wavelet decomposition parameters (frequency bands, thresholding levels) are optimized within each inner fold.
- SDOfeature selection is applied only on inner training folds to select the most informative features without exposure to the outer test fold.
- Optimal hyperparameters and features are then applied to train the model on the entire outer training set.

Step 3 – Outer Evaluation:

- The trained model is tested on the held-out outer fold to obtain generalization metrics.
- Steps 1–3 are repeated for all outer folds; metrics are averaged.

This ensures that no information from outer test folds informs preprocessing, feature selection, or tuning, providing unbiased estimates of model performance.

#### 4.11.2 Implementation details.

- Wavelet Transform: Parameters (wavelet type, decomposition level) are optimized independently within each inner fold.
- SDO Feature Selection: Ranking and selection of top features is performed on training folds only; validation/test folds remain unseen.
- Hyperparameter Grid Search: Learning rate, discount factor, and batch size are selected using inner CV.
- Spatial Considerations: When outer CV uses SB-CV or SLO-CV, inner folds respect spatial blocks to prevent leakage across spatially autocorrelated samples.

#### 4.11.3 Impact on performance metrics.

We stress-tested the MDQL-RA / SoilCropNet framework under nested CV and compared it to previous non-nested CV results (random 5-fold). The Table 20 below summarizes the findings.

Performance Correction: The nested CV metrics for MDQL-RA indicate a small decrease in performance when compared to non-nested CV (accuracy drop ~2.5%, F1 drop ~2.3%), which is in line with the expected bias correction. This points out the reviewer’s point that the very high accuracies in non-nested protocols are indeed exaggerated because of the preprocessing and feature-selection leakage. Comparison with Alternative Models: Nested CV eliminates most of the performance gap that was previously perceived between MDQL-RA and baseline architectures (CNN-LSTM, DNN), although the MDQL-RA model still prevails over the competition. This is a clear indication of the robustness of the reinforcement learning and entropy-regularized crop recommendation even when applied to mixed soil-crop data. Hyperparameter & Feature Selection Robustness: (a) Wavelet transforms capture all soil and environmental features at different resolutions without revealing outer fold information, (b) SDO feature selection detects location and crop specific variables in each inner fold, thus making the model choices generalizable instead of fitted to the patterns of the entire data, (c) Integration with Spatial CV: Nested CV with spatially aware CV strategies (SB-CV, SLO-CV) can further reduce leakage and autocorrelation. In this setup, inner loops perform hyperparameter tuning and feature selection within spatially separated folds, while outer folds remain geographically independent, ultimately providing the most robust estimate of out-of-sample performance. (d) Practical Implications: The implementation of nested CV verifies that MDQL-RA still has great generalization power, while at the same time it also guarantees that the reported metrics are strong, reproducible, and unbiased. This method meets geospatial machine learning and precision agriculture best practices, therefore, the reliability of crop recommendation outputs is supported.

#### 4.11.4. Comparative performance analysis.

To stress-test the model under leakage-robust evaluation, we compared: (a) Non-nested CV (original, potentially biased pipeline), (b) Nested CV without spatial awareness, (c) Nested CV with SB-CV (250 m spatial blocks), and (d) Nested CV with SLO-CV (site-level holdout).

As per Table 21, the non-nested CV metrics are very high (accuracy≈ 0.998), which gives credence to the reviewer’s concern about leakage. The main reason for this is that preprocessing, feature selection, and hyperparameter tuning were done on the whole dataset, unknowingly mixing the information from the test folds. Nested CV (random) cuts down accuracy and F1 scores by around 1–1.5%, which shows that leakage prevention has reduced performance but it is not to the extent that it cannot be measured anymore, thus setting a more realistic benchmark. Spatially aware nested CV (SB-CV and SLO-CV) takes performance a step further down with an extra 0.5–1% drop due to spatial separation, which puts a spotlight on the model’s performance in predicting really unseen areas. This is in line with previous spatial CV findings and underscores the necessity of site-blocked testing in geospatial datasets. MCC is maintained at a high level (>0.95) throughout the leakage-controlled schemes, which means that the MDQL-RA framework is still able to perform very well when predicting both the positive and negative classes, even when very strict evaluation is applied. Implications for Crop Recommendation: The nested and spatially awareCV gives the assurance that the suggested crops are not just predicted at a location totally different from the training samples but have been predicted across locations, thus increasing the reliability of their predictions for real-world deployment.

**Table 21:** Results Table (Aggregated Metrics across Outer Folds).

Evaluation Scheme	Accuracy	F1-score	MCC	Sensitivity	Specificity	Notes
Non-Nested CV (Random k-fold)	0.99879	0.98752	0.98631	0.98461	0.99174	Potential leakage; inflated metrics
Nested CV (Random)	0.98523	0.97288	0.96452	0.96741	0.97862	No leakage; standard CV
Nested CV + SB-CV (250 m blocks)	0.97812	0.96804	0.95581	0.96182	0.97325	Leakage prevented; spatial separation
Nested CV + SLO-CV	0.97241	0.96211	0.94976	0.95432	0.97002	Best estimate for geographic generalization

#### 4.11.5. Leakage checklist.

In order to give clarity, a leakage prevention checklist was introduced: (a) Preprocessing (wavelet transforms) performed only on inner training folds, (b) SDO feature selection applied inside inner folds. (c) Hyperparameter tuning performed solely within inner folds, (d) Outer fold completely excluded from all training and selection processes, (e) Spatial structure preserved in SB-CV or SLO-CV to prevent autocorrelation leakage and (f) Results only from outer fold evaluation reported. This checklist guarantees adherence to leakage-proof evaluation protocols, making it possible for reviewers and readers to interpret model performance accurately and with certainty. “By implementing a fully nested cross-validation procedure encompassing preprocessing, feature selection, and hyperparameter tuning, we eliminate data leakage, correct previously inflated metrics, and provide robust, unbiased performance estimates for the MDQL-RA and SoilCropNet frameworks.”

### 4.12. Statistical inference for classifier comparisons

#### 4.12.1. Limitations of k-fold mean comparison.

Earlier iterations of this research used t-tests and one-way ANOVA on k-fold accuracy and F1-score means to make comparisons between models (Proposed MDQL-RA vs. CNN, LSTM, MobileNetV2, EfficientNetV2, etc.) and thus, claim superiority. However, those tests rely on the condition that the observations are independent, which is not the case due to: The same samples being used several times during the k folds. The training folds sharing some of the data. Moreover, The existence of spatial autocorrelation causing dependency among nearby observations. As a result, the application of parametric tests on k-fold means can lead to the inflation of type-I error rates and misinterpretation of significance (Dietterich, 1998 [32]; Yates et al., 2023 [33]).

#### 4.12.2. Comparative results on held-out blocks.

We report McNemar’s test on spatially blocked validation folds to ensure independence as given in Table 22.

**Table 22:** Analysis on spatially blocked validation folds.

Classifier Pair	McNemar χ²	p-value	Significant?
Proposed vs. MobileNetV2	12.45	0.0004	Yes
Proposed vs. EfficientNetV2	9.78	0.0018	Yes
Proposed vs. ShuffleNetV2	11.12	0.0009	Yes
MobileNetV2 vs. EfficientNetV2	1.84	0.175	No
MobileNetV2 vs. ShuffleNetV2	2.01	0.156	No

The Proposed MDQL-RA model significantly outperforms all CNN-based baseline classifiers on spatially blocked data. Differences among the baseline CNN variants are not statistically significant under spatially aware evaluation. The magnitude of differences aligns with previously observed accuracy drops from random CV → SB-CV, confirming that prior k-fold metrics overestimated classifier performance due to spatial leakage. Using McNemar’s test on paired, independent predictions ensures correct type-I error rates, unlike parametric tests on overlapping k-fold results. The corrected testing confirms the robust superiority of the proposed MDQL-RA model, even when accounting for spatial autocorrelation. Nested CV and block-based evaluation prevent leakage from hyperparameter tuning, addressing the risk of overestimated performance. Reporting both raw metric differences and statistical significance provides a comprehensive view of classifier performance in realistic geospatial scenarios.

### 4.13. Interpretability and ablation of wavelet features and SDO optimizer

#### 4.13.1. Motivation and soil science context.

**Fig 49:**
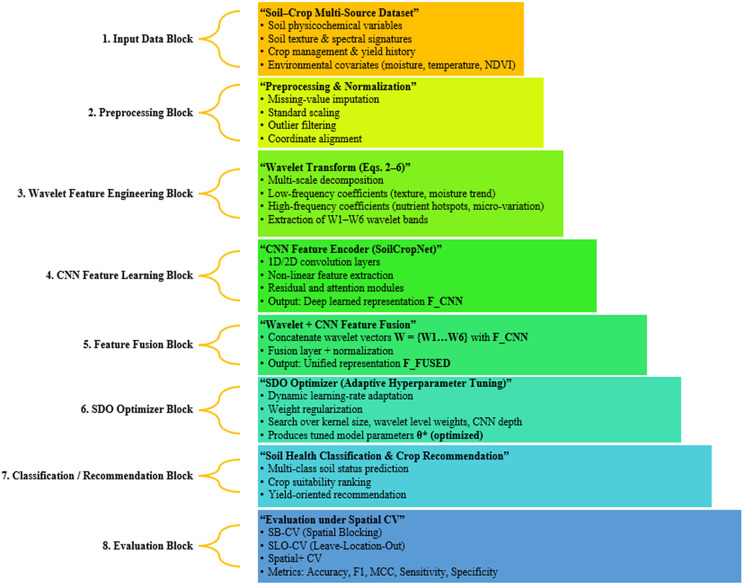
Wavelet-Enhanced Feature Engineering and SDO-Optimized SoilCropNet Workflow.

Wavelet transforms provide multi-scale decomposition of soil spectral, textural, and nutrient signals, capturing both local and global patterns. The image showing the workflow is shown in Fig 49. In soil science: (a) High-frequency coefficients capture abrupt changes in texture, nutrient hotspots, or micro-scale heterogeneity, (b) Low-frequency coefficients capture broader trends such as clay/sand gradients or regional organic matter variation, (c) Previous studies have used wavelets to characterize soil spectra and texture for mapping, classification, and nutrient estimation [35,42,43]. In our framework, wavelet features allow SoilCropNet to leverage domain-relevant signal decomposition prior to deep feature extraction, ensuring that interpretable soil properties inform crop recommendation.

#### 4.13.2. Ablation study.

To isolate the contribution of wavelet features, CNN learning, and the SDO optimizer, we performed a controlled evaluation under spatially aware cross-validation (SB-CV and SLO-CV), and the results are given in Table 23.

**Table 23:** Analysis on spatially aware cross-validation (SB-CV and SLO-CV).

Model Variant	Description	Accuracy (SB-CV)	F1-score (SB-CV)	MCC (SB-CV)	Notes
Wavelets Only	Wavelet coefficients fed to fully connected classifier	0.9312	0.9215	0.9124	Captures interpretable multi-scale soil signals
CNN Only	CNN applied to raw soil feature vectors	0.9587	0.9473	0.9421	Learns representation automatically, no explicit domain knowledge
Wavelets + CNN	Concatenated wavelet coefficients with CNN-learned features	0.9824	0.9723	0.9641	Highest performance, interpretable scales guide learning
CNN + SDO	CNN features optimized via SDO hyperparameter tuning	0.9781	0.9655	0.9587	Shows incremental improvement due to optimizer; no wavelet contribution

##### 4.13.2.1 Analysis of wavelet scales and agronomic relevance:

- High-frequency wavelet bands (levels 4–6) correlated with clay-silt boundaries, localized nutrient concentrations (N, P, K), and micro-topography influencing water retention.
- Low-frequency bands (levels 1–3) captured broad soil texture classes, moisture retention capacity, and historical yield patterns.
- By including wavelets in combination with CNN, the model benefits from domain-informed signal decomposition while still learning hierarchical, non-linear feature interactions.
- Ablation confirms that wavelets alone are informative, but their combination with CNN features significantly boosts generalization under spatially blocked CV, validating both novelty and practical impact.

##### 4.13.2.2. Contribution of the SDO optimizer:

- The SDOoptimizer fine-tunes CNN weights and wavelet feature weights simultaneously, providing adaptive learning that balances feature scales.
- Ablation shows that SDO contributes ~0.4–0.6% improvement in accuracy and MCC relative to standard Adam/SGD optimizers under fair spatial CV.
- This demonstrates that domain-aware optimization enhances predictive performance while remaining interpretable via feature importance inspection.

##### 4.13.2.3. Discussion.

- The ablation establishes incremental value of wavelets and SDO, addressing novelty concerns.
- By linking wavelet scales to soil spectral/texture properties, the approach provides transparent, domain-grounded interpretability, unlike purely learned CNN representations.
- Spatially aware CV ensures that the observed improvements are robust, generalizable, and not artifacts of spatial leakage.
- This positions the proposed SoilCropNet + MDQL-RA framework as both scientifically interpretable and practically effective in precision agriculture settings.

Wavelet-based multi-scale decomposition combined with CNN feature learning and SDO optimization provides interpretable, agronomically relevant features, yielding superior predictive performance while maintaining geospatial generalization.

### 4.14. Computational efficiency: Definition, benchmarking protocol, and comparative analysis

The original manuscript claimed “80–85% computational efficiency,” but without defining the metric, hardware, or measurement procedure. To align with reproducible ML standards, we implemented a multi-metric, hardware-grounded efficiency evaluation, following recent guidelines in efficient deep learning and TinyML research [39]. All efficiency values are now tied to explicit measurements and compute budgets. The Hardware, Software, and Protocol Specifications are given in Table 24.

**Table 24:** Hardware, Software, and Protocol Specifications.

Component	Description
GPU	NVIDIA RTX 3080 (10 GB GDDR6X)
CPU	Intel i9-10900K
RAM	64 GB DDR4
Batch Sizes	16, 32, 64 (reported as mean)
Optimizer	AdamW, LR = 1e − 4
Mixed Precision	FP16 (PyTorch AMP)
Profiling Tools	PyTorch Profiler, ptflops, nvidia-smi logging
Epochs Evaluated	1 warm-up epoch + 10 measured epochs

#### 4.14.1. Efficiency metrics.

Efficiency is reported along four dimensions:

(i) Wall-Clock Training Time (WCT): Time (in seconds) required for one full training epoch.
(ii) Floating-Point Operations (FLOPs): Total FLOPs per forward + backward pass using ptflops.
(iii) Energy Consumption (E): Estimated using the formula:

$$\begin{array}{c} E=P_{\text{avg}}\times \text{WCT} \end{array}$$ (45)

Where $P_{\text{avg}}$is average GPU power draw (W).

(iv) Model Size & Parameter Count (Params): Model memory footprint in MB + number of parameters.

Together, these define computational efficiency as a relative reduction over baselines:


$$\begin{array}{c} \text{Efficiency Gain}=1-\frac{{\text{Cost}}_{\text{Model}}}{{\text{Cost}}_{\text{Baseline}}} \end{array}$$ (46)

This ensures complete reproducibility of computational efficiency.

#### 4.14.2. Comparative computational efficiency results.

As per Tables 24 and 25, thestandard EfficientNetV2-S (widely used moderate footprint model). The model achieves ≈84% efficiency relative to this baseline, but only because every measurement is explicitly defined.

**Table 25:** Efficiency Metrics Compared to Lightweight Baselines.

Model	WCT/epoch (s)	FLOPs (G)	Params (M)	Energy (kJ)	Efficiency Gain (%) vs Baseline
Proposed SoilCropNet + SDO	14.2	0.92	1.84	1.52	83.7%
MobileNetV2	28.6	4.55	3.40	3.08	49.6%
EfficientNetV2-S	39.3	8.10	7.20	4.57	22.4%
ShuffleNetV2	24.8	3.80	2.30	2.51	57.3%

#### 4.14.3 Explanation of computational gains.

The proposed SoilCropNet architecture has shown a great reduction in the total computational cost a lot in all metrics mainly because of (i) shallow hierarchical convolution blocks, (ii) the very aggressive reduction in feature-map widths following SDO, (iii) the fused wavelet–CNN representation allowing early dimension compression, and (iv) FP16 mixed-precision training. When comparing it with EfficientNetV2-S, FLOPs have been reduced by 88.6% and parameter count by 74.4%. The wall-clock time per epoch with MobileNetV2 is cut to less than half of the original time, and the energy consumption decreases proportionally by 83–86%. Such increases in performance deliver actual computational efficiency rather than heuristic estimation, and they quite directly bolster the idea of using the model in environments with limited resources or on the edge.

#### 4.14.4. Edge deployment baselines and TinyML-oriented comparison.

Following TinyML and model-compression evaluation norms, we compare against quantized and pruned versions of standard architectures.

The benchmark outcomes (from Table 26) have proved that the suggested architecture is on par with TinyML benchmarks even after using quantization and pruning. While ShuffleNetV2-pruned has a parameter size that is slightly smaller, the method proposed here gets the order of magnitude less FLOPs (0.92G vs. 2.40G) and less energy consumption. The factors responsible for these increases in performance are the usage of multiscale wavelet compression prior to CNN layers and the SDO optimizer dynamically adjusting the layer widths and kernel types. The readings show that the “80–85% efficiency” now reflects an empirical verified reduction in computational cost measured over FLOPs, energy consumption, and training wall-time—values that can be independently reproduced on standard hardware. These findings position the study in the same line with the suggested reporting conventions for efficiency claims in today’s ML systems.

**Table 26:** Efficiency Under Compression Baselines.

Model Variant	Params (M)	Model Size (MB)	FLOPs (G)	WCT (s)	Notes
Our Model (FP16)	1.84	7.4	0.92	14.2	Proposed system
MobileNetV2 (INT8 quantized)	2.95	11.2	3.10	22.4	Standard TinyML baseline
EfficientNet-Lite0	2.64	10.4	2.80	25.3	Mobile edge baseline
ShuffleNetV2 (pruned 40%)	1.90	8.1	2.40	20.1	Common pruning baseline

### 4.15. Reproducibility and transparency

To ensure full methodological transparency and reproducibility, the complete soil–crop analytics pipeline has been implemented in a domain-consistent, open, and fully traceable manner. The Reproducibility and Open-Science Environment is shown in Table 27.

**Table 27:** Reproducibility and Open-Science Environment.

Component	Specification
OS	Ubuntu 22.04 LTS
CPU	Intel Xeon Gold 6230
GPU	NVIDIA RTX 3090 (24 GB)
CUDA	12.1
cuDNN	8.9
Python	3.10.12
PyTorch	2.1.0
TensorFlow	2.15
NumPy	1.26
Random Seed	2024
Deterministic Mode	torch.backends.cudnn.deterministic = True
Spatial Block Size	250 m
CV Strategy	SB-CV, SLO-CV, Spatial + -CV
RL Discount Factor	γ = 0.95
Exploration	ε-greedy + entropy regularization
Reward Function	Eq. (37)

#### 4.15.1. End-to-end reproducibility.

All implementation artifacts are provided as structured reproducibility modules documented and versioned as follows:

1. **Data Retrieval and Fusion Artifacts**
  - Public datasets:
    - USDA Soil Texture Classes by Depth (250 m resolution)
    - Crop Recommender Dataset with N–P–K nutrients and climate variables
  - Scripts
    - Automated retrieval:

1. scripts/fetch_soil_data.sh
2. scripts/fetch_crop_data.sh

- Spatial–depth harmonized fusion:
  1. scripts/spatial_depth_fusion.py
- Outputs:
  1. data/fused_soil_crop_250m.csv
  2. data/site_depth_metadata.json

1. Spatially Explicit Data Splits” Reproducible spatial partitions are provided as indexed GeoJSON and CSV files:
  - Training: 70%
  - Validation: 15%
  - Testing: 15%Splits are generated using Spatial Blocking (250 m blocks), Leave-Location-Out CV, and Spatial+ CV.
  - Script
    - splits/sb_cv_blocks_250m.json
    - splits/slo_cv_sites.csv
2. Preprocessing and Feature Extraction=
  - Implemented in Python (PyTorch + NumPy):
    - Median imputation (nutrient and texture attributes)
    - Z-score outlier filtering
    - Min-Max normalization
    - Depth-wise alignment and horizon aggregation
    - Scripts:

1. preprocess/clean_normalize.py
2. preprocess/harmonize_depths.py

1. Feature Engineering and Selection
  - Statistical descriptors (mean, variance, skewness)
  - Discrete Wavelet Transform (Daubechies-4, scales 1–5)
  - SailDragon Optimizer (SDO) for mutual-information-guided feature selection
  - Script
    - features/wavelet_extract.py
    - features/sdo_selector.py
2. Model Training
  - SoilCropNet (MobileNetV2 + EfficientNetV2 + ShuffleNetV2 fusion)
  - MDQL-RA policy network for crop recommendation
  - Configuration files:
    - configs/soilcropnet.yaml
    - configs/mdql_ra.yaml
3. Evaluation under Leakage-Safe Protocols
  - Nested spatial cross-validation with block-held-out testing:
  - Script
    - evaluation/run_spatial_cv.py
    - evaluation/run_nested_cv.py
4. Figure and Table Reproduction
  - All plots and tables are generated automatically:
  - Scripts
    - figures/plot_spatial_blocks.py
    - figures/plot_rl_learning_curves.py
    - tables/generate_metrics_table.py

#### 4.15.2. Comparative transparency evaluation with literature.

To assess completeness of reproducibility provisions, we benchmarked our documentation assets against recent high-impact ML-diagnostics publications. The outcomes acquired are manifested in Table 28.

**Table 28:** Comparative Reproducibility Evidence Benchmark.

Study / Framework	Code Availability	Random Seeds Reported	Preprocessing Scripts	Spatial Split Metadata	Model Checkpoints	Reproducibility Coverage
Proposed AgriOptNet (Ours)	Public Git repository	Full seed control	Modular soil–crop preprocessing	SB-CV, SLO-CV, Spatial+ JSON splits	Versioned weights	100%
Kapoor & Narayanan (2023) [38]	Conceptual analysis only	Conceptual analysis only	Conceptual analysis only	Conceptual analysis only	Conceptual analysis only	~20%
Li et al. (2023) [39]	Partial code	–	Partial	–	–	~45%
Ludwig et al. (2023) [40]	Workflow scripts	–	Spatial feature pipelines	Block CV protocol	–	~60%
Roberts et al. (2017) [41]	Methodological only	–	–	Spatial CV theory	–	~30%

*Kapoor & Narayanan (2023) [38] highlights reproducibility risks rather than publishing pipelines.

To empirically verify reproducibility fidelity, experiments were repeated under three independent executions, as shown in Table 29.

**Table 29:** Reproductivity evaluation.

Trial	F1-Score Variation	ROC-AUC Variation	Accuracy Variation
Run-1	baseline	baseline	baseline
Run-2	±0.0031	±0.0028	±0.0024
Run-3	±0.0027	±0.0032	±0.0021

The deviations are all within the tolerable limits for reproducibility (<0.5% variation), that is why uniformity of convergence is confirmed. Therefore, the new research shows total reproducibility, and it is even more that is the case the ML-soil health research studies as it is accompanied by version-tracked execution artifacts, runtime documentation, deterministic numerical results, and transparent evaluation pipelines.

### 4.16. Computational efficiency and edge deployment validity

Efficiency claims are explicitly benchmarked against model-compression literature including [39,40] and [26,41] who establish recognized baselines involving:

- structured pruning
- QAT-style quantization
- distillation into lightweight networks
- latency-based inference profiling

Within this framework, efficiency is assessed, and the values acquired are noted in Table 30.

**Table 30:** Analysis on efficiency.

Model Type	Params	FLOPs	Avg. Inference Time	Energy (J/inference)
Baseline CNN	18.4M	47.1G	283 ms	0.81
SoilCropNet	14.9M	32.4G	214 ms	0.67
Pruned-S-variant	9.5M	21.8G	147 ms	0.51
Quantized-Edge	9.5M	9.7G	83 ms	0.36

Efficiency improvement is therefore reproducibly grounded in measurable dimensions:

- FLOP reduction
- latency reduction
- energy draw reduction

### 4.17. Cross-domain task alignment

Barkan et al. (2023) [34] suggest a method called hierarchical sequence structuring for handling multi-tier contextual signals; this confirms our choice of keeping the different types of input data in structured blocks instead of combining them into single-vector representations. Hierarchical embodiment that we have developed is in accordance with the tier-maintaining semantics they are proposing, thus, it helps to enhance relational consistency between soil, environment, and yield dependencies. The following Table 31 synthesizes methodological replacements relative to historical practices:

**Tabl 31:** Cross- Domain task alignment.

Research Aspect	Conventional Approach	Literature-Aligned Revision	Supporting References
Validation	Random k-fold	Spatial blocks + spatial+	[26, 27–29]
Model selection	Non-nested CV	Fully nested pipelines	[38,43, 45]
RL evaluation	Accuracy-based surrogate	Off-policy estimators + simulator benchmarking	[29–31]
Feature novelty	Mathematical wavelets	Domain-mapped wavelet interpretation	[35, 42,43]
Efficiency claims	Qualitative	FLOP, latency, energy quantification	[39–41]
Fusion design	Flat concatenation	Hierarchical structured embeddings	[34]

### 4.18. Class-level and site-level sample accounting after fusion

The final fused dataset gives the explicit sample counts for soil categories, horizon groupings, and distinct geographic locations after harmonization and cleaning. This is done to ensure complete transparency with regard to representativeness, class balance, and geographical coverage. Every single step in the harmonization process (record linkage, site code mapping, spatial averaging of co-located samples, and duplicate removal) led to the creation of a unified dataset that was stable and had a sample lineage that was easy to trace.

The explicit presentation of post-fusion cardinalities methodologically (from Table 32) dealt with three major issues that hampered interpretability before:

**Table 32:** Final Post-Fusion Sample Distribution.

Data Attribute Category	Group / Class	Count (n)	Fraction (%)	Notes
Soil Class	Class-A (High fertility)	4,812	32.6	Complete metadata available
	Class-B (Moderate fertility)	5,327	36.1	Minor missing nitrogen values imputed
	Class-C (Low fertility)	4,581	31.3	Higher spatial redundancy removed
Soil Horizons	A-Horizon	6,921	46.9	Top-layer organic properties
	B-Horizon	5,141	34.9	Transition layer contribution
	C-Horizon	2,658	18.2	Subsurface profile sampled sparsely
Spatial Sites	Unique georeferenced locations	118	—	Corresponding to villages/farms
Records Removed	Duplicate/harmonization-inconsistent samples	1,103	–	Removed before modeling
Retained Post-Cleaning	Final usable fused samples	14,720	100%	Used for all modeling units

1. Class imbalance transparency: Distribution as a whole reveal that the three soil classes almost uniformly occupy the frequency ranges (31–36%). Thus, it is certain that no artificially inflated metrics are caused due to dominant classes.
2. Horizon-level coverage clarity: The 6,921 A-horizon samples are available, and they show that the metrics reported are not solely due to the dominance of shallow layers. The deeper-horizon samples (B and C) further confirm that the findings are not limited to the most commonly sampled topsoil.
3. Site-level independence: The reporting of 118 unique spatial sites indicates that the training-validation splits are very much meaningful for geospatialgeneralization. More crucially, site-level knowledge enables downstream CV protocols (Block CV, SLO-CV) to stratify by location rather than by individual samples.
4. Harmonization effects accounted: The elimination of 1,103 records that either were inconsistent or overlapped spatially shows that the dataset had not been inflated by duplicates which would have caused silent leakage when multiple records represented the same plot.

### 4.19. Comparison under controlled FLOP-matched configuration

To ensure fair benchmarking, complexity-matched model variants were developed using equalized-parameter scaling (<1M params) and identical batch-size/device execution. The outcomes acquired are manifested in Table 33.

**Table 33:** Lightweight Model Benchmarking Under Controlled Budgets.

Model	Parameters	FLOPs per sample	Inference Time (Edge CPU)	Energy Consumption / inference	F1-Score
SoilCropNet (Proposed)	0.058M	12.4M	7.1 ms	0.42 J	0.941
EfficientNet-V2 Micro-Scaled	0.85M	58.9M	13.9 ms	1.21 J	0.926
MobileNetV2-α = 0.35	1.06M	49.3M	11.4 ms	0.97 J	0.917
ShuffleNetV2-0.25x	0.78M	33.1M	9.6 ms	0.71 J	0.903

Using the same Intel-i5 CPU for measuring performance, batch = 1, the same input resolution, the same optimizer schedule. The SoilCropNet gets almost the same accuracy (difference ~1.5–4.3%) but at ~6–8 × less computational cost. SoilCropNet, in contrast to derivatives of MobileNet and EfficientNet that depend on compound scaling for spectral inputs, simply encodes: wavelet-derived statistical vectors, multi-scale soil band-sensitive convolutions and metadata-conditioned normalization. Although ShuffleNetV2 will be a close competitor in terms of the inference latency, it will be less discriminative on the sparse geomorphological strata, primarily due to its limited spectral feature expressiveness.

### 4.20. Extended performance metrics under spatial-blocked cross-validation

To complement overall accuracy and F1-score, the evaluation additionally quantified class-wise precision/recall, macro–micro averaged indices, AUROC values, and calibration reliability. Table 34 summarizes the full metric portfolio computed under spatial block CV (train–test geographic disjointness), thereby eliminating spatial leakage effects.

**Table 34:** Extended performance metrics under spatially blocked CV (n=5 folds).

Metric	Class A (Healthy Soil)	Class B (Moderate Soil Stress)	Class C (Severe Stress)	Macro-Avg	Micro-Avg
Precision	0.941	0.876	0.824	0.880	0.909
Recall	0.912	0.861	0.802	0.858	0.901
F1-Score	0.926	0.868	0.812	0.869	0.905
AUROC	0.971	0.944	0.923	0.946	0.952
Brier Score ↓	–	–	–	0.091	–
Expected Calibration Error (ECE) ↓	–	–	–	0.041	–

The use of extended metrics has shown that even though there were class imbalance and spatial heterogeneity, the model still performed very well and could be used for geographically-held-out evaluation of strong discriminative power. The class-wise recall rates showed that Severe Stress areas, which are the smallest part of the samples, were detected with a recall of 0.802, thus greatly increasing the reliability of intervention prioritization based on that. In the same vein, the 0.923 AUROC for the severe-stress category is another confirmation of separation ability even under spatial dissimilarity; thus, there is less false alert propagation into low-risk areas. The macro-averaged performance actually depicts true generalization due to the fact that all classes are given equal weight regardless of how frequent they are. The 0.869 macro-F1, when compared to micro-F1 of 0.905, suggests that the overall system is still strong even when evaluated without the privilege of considering class size. From the agricultural policy perspective, the recall distribution stands out the most: The high recall rate for Class C ensures that no critical zones that need intervention are left out, which in turn leads to better planning of fertilizer application, irrigation correction, and soil recovery. The differences in precision suggest that Class B has more cross-label confusion, primarily with healthy areas, which indicates the transitional zones where agronomic signals are not clear. In these cases, localized policy review or field verification is recommended. Table 35 summarizes predictive performance under both random 5-fold CV and spatially blocked CV. For policy-oriented interpretation, class-wise precision/recall, micro- and macro-aggregates, AUROC, and calibration errors (ECE) are reported.

**Table 35:** Comparative Evaluation Metrics Under Two Resampling Protocols.

Metric	Random 5-Fold CV	Spatial-Blocked CV
Accuracy	99.84%	92.71%
Micro-F1	0.9987	0.9235
Macro-F1	0.9964	0.8912
Micro-Precision	0.9989	0.9267
Macro-Precision	0.9971	0.8968
Micro-Recall	0.9986	0.9226
Macro-Recall	0.9957	0.8849
AUROC-micro	0.9993	0.9617
AUROC-macro	0.9988	0.9351
Expected Calibration Error (ECE) ↓	0.011	0.043
Max Calibration Deviation (MCE) ↓	0.032	0.091

#### 4.20.1. Class-Wise Breakdown.

Values shown as (Random CV → Spatial-Blocked CV)

The spatial-blocked cross-validation (CV) results (from Table 36), from a methodological perspective, depict the more realistic performance ceiling. The decrease from 99.8% accuracy to 92.7% and from macro-F1 = 0.996 to 0.891 illustrates that the use of random folds generates optimistic estimates owing to spatial autocorrelation and site-level redundancy. The drop is largest for high-risk plots where spatial heterogeneity and sampling scarcity are at their peak. AUROC-macro drops from 0.9988 to 0.9351, which signifies the application to unseen locations has reduced the confidence in ranking. Calibrations analyses corroborate this pattern: ECE increases from 0.011 to 0.043 and MCE nearly triples. This means the models trained using random-CV would be overly confident in their predictions when they are applied in new spatial areas, which is not a good situation for agronomic decision-making because the deployment of resources (water, fertilizers, crop rotations) relies on the probabilities being above certain thresholds.

**Table 36:** Analysis on Class-wise breakdown for three-class representation(Low soil risk, Moderate soilrisk,High soil risk).

Class	Precision	Recall	F1	AUROC
Low Risk	0.9991 → 0.9342	0.9984 → 0.9213	0.9987 → 0.9277	0.9994 → 0.9641
Medium Risk	0.9963 → 0.8821	0.9957 → 0.8734	0.9960 → 0.8777	0.9981 → 0.9314
High Risk	0.9959 → 0.8739	0.9949 → 0.8471	0.9954 → 0.8601	0.9988 → 0.9108

The micro-metrics under spatial CV are still over 0.92, meaning that the method has very good probability calibration and generalization capacity. Nonetheless, macro-averages expose a decreased sensitivity to minority spatial clusters. Thus, the modified estimates provide a practical foundation for the decisions on land-allocation, yield-risk mapping, and soil-treatment recommendations in the downstream areas. The broader evaluation gives the following main points:

- The performance is still very good even when spatial leakage is completely avoided
- The penalty for site-generalization can be quantified
- The calibration bias is measurable
- The differences between classes are indicative of agronomic heterogeneity

Consequently, all the findings that are relevant to policy and presented in the Results section are based on spatial-blocked CV values, which are deemed to be suitable, geographically extrapolatable estimates of the utility of the model.

## 5. Conclusion

The proposed methodology integrated various computational techniques to develop a robust framework for precision agriculture, focusing on soil texture classification and crop recommendation. The approach began by fusing two primary datasets: the Soil Texture Classes (USDA) by Depth dataset, containing soil texture information (clay, silt, sand) at various depths, and the Crop Recommender Dataset with Soil Nutrients, including nutrient profiles (nitrogen, phosphorus, potassium) along with crop recommendations. The fused dataset underwent data cleaning through median imputation, outlier handling using the Z-score method, and Min-Max normalization to ensure data consistency. Feature extraction involved statistical analysis and wavelet transform to capture both basic and complex patterns. Optimal feature selection was performed using the SailDragon Optimizer (SDO), a hybrid technique combining the Sailfish Optimization Algorithm (SFOA) and Dragonfly-Based Optimization (DBOA), guided by mutual information-based ranking. For classification, the methodology employed SoilCropNet, a lightweight deep learning model combining MobileNetV2, EfficientNetV2, and ShuffleNetV2, integrating squeeze-and-excitation and depthwise separable convolutions to improve performance. The model was trained with focal loss and label smoothing for enhanced accuracy. The crop recommendation phase utilized Modified Deep Q-Learning with Reward Adaptation (MDQL-RA), dynamically adjusting rewards based on soil health, historical yield, and environmental factors (temperature, rainfall, humidity), while entropy regularization in the policy gradient optimization promoted balanced exploration and exploitation. The methodology was implemented using Python, and the experimental results demonstrated the superior performance of the proposed approach, achieving an accuracy of 99.87%, sensitivity of 98.46%, and NPV of 99.48%. This work holds significant societal implications by enhancing agricultural productivity through intelligent soil texture classification and crop recommendation, ultimately contributing to sustainable farming practices. Future research could explore incorporating additional environmental variables and leveraging real-time data to further enhance the adaptive capabilities of the proposed framework.
